# Aneurysm Is Restricted by CD34^+^ Cell‐Formed Fibrous Collars Through the PDGFRb‐PI3K Axis

**DOI:** 10.1002/advs.202408996

**Published:** 2024-12-27

**Authors:** Hong Wu, Xiaoping Yang, Ting Chen, Baoqi Yu, Mengjia Chen, Ting Wang, Liujun Jiang, Bohuan Zhang, Xuhao Zhou, Junning Cheng, Kai Chen, Tao Zhang, Yanhua Hu, Simon Xu, Jiangfang Lian, Hongkun Zhang, Qingzhong Xiao, Honghua Ye, Qingbo Xu

**Affiliations:** ^1^ Department of Cardiology The First Affiliated Hospital Zhejiang University School of Medicine Hangzhou 310003 China; ^2^ Department of Cardiology Ningbo Institute of Innovation for Combined Medicine and Engineering Lihuili Hospital Affiliated to Ningbo University Ningbo University Ningbo Zhejiang 315000 China; ^3^ Department of Physiology and Pathophysiology School of Basic Medical Sciences Capital Medical University Key Laboratory of Remodeling‐Related Cardiovascular Diseases Ministry of Education Beijing Key Laboratory of Metabolic Disorder‐Related Cardiovascular Diseases Beijing 100069 China; ^4^ Department of Vascular Surgery Peking University People's Hospital Beijing 100044 China; ^5^ Department of Surgery Liverpool Heart and Chest Hospital Liverpool L14 3PE UK; ^6^ Department of Vascular Surgery The First Affiliated Hospital Zhejiang University School of Medicine Hangzhou 310003 China; ^7^ Centre for Clinical Pharmacology and Precision Medicine William Harvey Research Institute Faculty of Medicine and Dentistry Queen Mary University of London London EC1M 6BQ UK

**Keywords:** aneurysms, CD34^+^ cells, fibroblasts, genetic cell lineage tracings, PDGFRb

## Abstract

Aortic aneurysm is a life‐threatening disease caused by progressive dilation of the aorta and weakened aortic walls. Its pathogenesis involves an imbalance between connective tissue repair and degradation. CD34^+^ cells comprise a heterogeneous population that exhibits stem cell and progenitor cell properties. However, the role of CD34^+^ cells in abdominal aortic aneurysm (AAA) remains unclear. In this study, downregulated CD34 expression is observed in aneurysmal aortas from both patients and mouse models compared to that in non‐dilated aortas. Furthermore, by combining *Cd34*‐CreER^T2^;Rosa26‐tdTomato;(*Apoe^−/−^*) lineage tracing, bone marrow transplantation, and single‐cell sequencing, it is found that during AAA development, non‐bone marrow CD34^+^ cells are activated to transdifferentiate into Periostin^+^ myofibroblasts, thereby contributing to the formation of fibrotic collars. Dual recombinase‐based lineage tracing confirms the presence and involvement of CD34^+^/Periostin^+^ myofibroblasts in fibrotic collar formation during AAA development. Functionally, selective depletion of systemic or non‐bone marrow CD34^+^ cells, as well as CD34^+^/Periostin^+^ myofibroblasts, by diphtheria toxin significantly exacerbates AAA progression and increases disease mortality. Mechanistically, it is identified that the PDGF‐PDGFRb‐PI3K axis is indispensable for Periostin^+^ myofibroblast generation from non‐bone marrow CD34^+^ cells in AAA, offering a new therapeutic target for patients with AAA at a high risk of rupture.

## Graphical abstract

The study unveils a previously unknown role of CD34^+^ cells in the pathogenesis of AAAs, shedding light on their contribution to tissue repair and aneurysmal fibrous collar formation. Non‐bone marrow CD34^+^ cells undergo transdifferentiation into Periostin^+^ fibroblasts, a process with important implications in protecting AAA from rupture. The involvement of the PDGF‐PDGFRb‐PI3K axis in CD34^+^ cell activation and transdifferentiation into Periostin^+^ myofibroblasts adds a novel mechanistic insight into AAA pathogenesis.

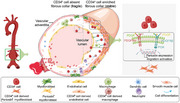

## 1 Introduction

Abdominal aortic aneurysm (AAA) is among the most severe vascular diseases and is characterized by progressive dilation of the abdominal aorta and localized weakness of the arterial wall, potentially leading to rupture.^[^ ^1^ ^]^ A study by DeRoo et al. revealed that the annual rupture risk increases considerably with AAA diameter (1–11% at 5.0–5.9 cm, 10–22% at 6.0–6.9 cm, and 30% at 7.0 cm).^[^ ^2^ ^]^ Numerous studies on the pathogenesis of aortic aneurysms have focused primarily on smooth muscle cell (SMC) apoptosis and phenotypic transformation, matrix metalloproteinase (MMP) dysregulation, endothelial cell (EC) dysfunction, exacerbated oxidative stress, and increased macrophage and T‐cell infiltration into vessel walls.^[^ ^3^ ^]^ Additionally, studies have confirmed that genetic mutations in proteins involved in extracellular matrix (ECM) synthesis and adhesion confer a high risk of aortic aneurysms and acute aortic dissections, such as *FBN1* mutations in Marfan syndrome and *COL3A1* mutations in vascular Ehlers–Danlos syndrome.^[^ ^4^ ^]^ Another study suggested a potential causal effect of abnormal collagen fibrils and pathological ECM remodeling in aortic aneurysms.^[^ ^5^ ^]^ Nevertheless, despite being the primary ECM‐synthesizing and secreting cells, the precise role of vascular adventitial fibroblasts in ECM remodeling specific to AAA remains unclear.

Fibroblasts are heterogeneous mesenchymal cells with diverse critical functions in tissue homeostasis and disease, including ECM synthesis, wound healing, epithelial differentiation, and inflammation regulation.^[^ ^6^ ^]^ Fibroblasts at different anatomical sites exhibit distinct characteristics and transcriptional patterns.^[^ ^7^ ^]^ Vascular adventitial fibroblasts facilitate ECM production to maintain the structural integrity of the vascular wall during the pathogenesis of aortic aneurysms.^[^ ^8^ ^]^ In response to injury and/or biomechanical stress, quiescent vascular adventitial fibroblasts are activated and transdifferentiate into ECM‐secreting fibroblasts or myofibroblasts, thereby promoting adventitial and biomechanical remodeling.^[^ ^9^ ^]^ Notably, the proportion of fibroblasts and myofibroblasts increases in the aneurysmal aorta, suggesting the role of increased myofibroblasts in aneurysms.^[^ ^10^ ^]^ Therefore, further exploration of the functional implications of myofibroblasts in AAA development and progression is warranted.

Several studies have reported that adventitial stem/progenitor cells expressing CD34, c‐Kit/stem cell factor receptor, or stem cell antigen‐1 (Sca‐1) differentiate into SMCs^[^ ^11^ ^]^ and ECs^[^ ^12^ ^]^ in vivo under various pathological conditions. We previously identified CD34^+^ cells as a heterogeneous population of fibroblasts, ECs, and inflammatory cells.^[^ ^13^ ^]^ Lineage tracing combined with single‐cell RNA sequencing studies has shown that CD34^+^ cells of non‐bone marrow (BM) origin generate fibroblasts during cardiac^[^ ^14^ ^]^ and kidney fibrosis.^[^ ^15^ ^]^ Three morphologically distinct aortic CD34^+^ cell populations were observed within the aneurysmal aortic wall.^[^ ^16^ ^]^ However, the exact identity and cellular heterogeneity of CD34^+^ cells remain unclear. Therefore, in this study, we investigated the putative role and fate of CD34^+^ cells in AAA. Herein, we observed downregulated expression of CD34 in both human and mouse aortic aneurysms and demonstrated that CD34^+^ cells give rise to Periostin^+^ myofibroblasts in AAA induced by both angiotensin II (Ang II) infusion and calcium chloride (CaCl_2_) adventitial application. Using bone marrow transplantation (BMT), diphtheria toxin receptor (DTR), and a dual recombinase‐based lineage tracing mouse model, we further revealed that non‐BM CD34^+^ cells, particularly CD34^+^/Periostin^+^ myofibroblasts, contribute to adventitial fibrotic collar formation, thereby protecting AAA from rupture. Mechanistically, we showed that the PDGFRb‐PI3K axis is required for platelet‐derived growth factor‐BB (PDGFBB)‐induced CD34^+^ cell activation and transdifferentiation into myofibroblasts in AAA.

## 2 Results

### 2.1 CD34 Expression is Decreased in Human and Mouse Aortic Aneurysms

**Figure 1:**
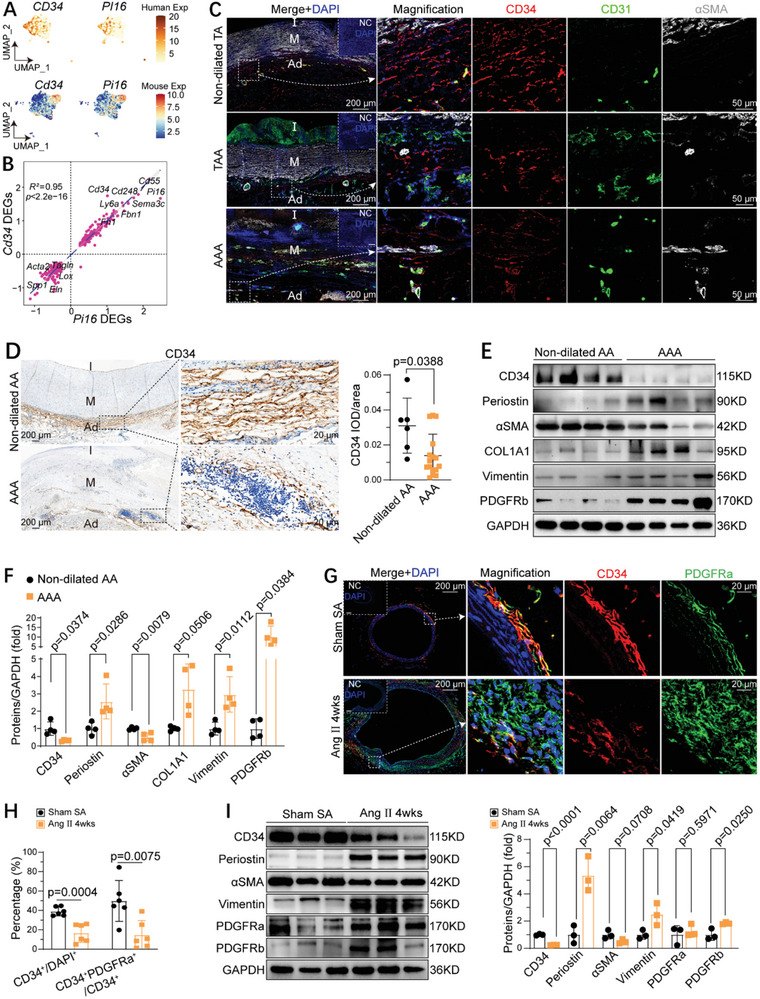
CD34 expression decreased in human and mouse aortic aneurysms. A) Feature plot showing the expression and distribution of *Cd34*/*CD34* and fibroblast progenitor marker genes Pi16/*PI16* in fibroblasts obtained from publicly available 10x single‐cell RNA sequencing datasets of human (GSE155468 and GSE166676) and mouse (GSE221789) normal and aneurysmal aortas, respectively. B) Scatterplot comparing fold changes of progenitor marker gene expression from *Cd34* ^high^ versus *Cd34* ^low^ cells (*y*‐axis: *Cd34* DEGs) against fold changes from *Pi16* ^high^ versus *Pi16* ^low^ cells (*x*‐axis: *Pi16* DEGs). DEGs were defined as (FDR < 0.05, fold change > 0.25, or < −0.25). *P* value and *R* ^2^ were calculated by R package ggpubr with Pearson correlation. C) Immunofluorescence (IF) staining for CD34 (red), CD31(green), and αSMA (grey) expression in human tissues of AAA and TAA as well as non‐dilated thoracic aorta (Non‐dilated TA), with magnification of the boxed region. D) Representative immunohistochemical (IHC) images illustrating CD34 expression pattern in human AAA and non‐dilated abdominal aorta (Non‐dilated AA), accompanied by magnified views of the boxed regions. The dot plot (right) displayed the average optical density of CD34‐positive staining in aortic adventitia across different groups (*n =* 6 and 16 individuals in the Non‐dilated AA and AAA group, respectively). E) Western blotting of CD34 and fibrosis‐related proteins in aortas from patients with AAA and Non‐dilated AA). *n =* 4 individualsper group. F) Quantification of the fold change of indicated proteins between human non‐dilated AA and AAA (*n =* 4 individuals per group). G) IF staining for CD34 and PDGFRa (platelet‐derived growth factor receptor A) on suprarenal aortic sections from *Apoe* ^−/−^ mice subjected to 4 weeks of Ang II infusion (Ang II 4 weeks) and sham‐treated mice (Sham SA), with the magnification of the boxed region. H) Quantification of the percentage of CD34^+^ cells in DAPI^+^ cells and CD34^+^PDGFRa^+^ cells in CD34^+^ cells in the indicated group. I) Western blotting of CD34 and fibrosis‐related proteins in aortas from *Apoe* ^−/−^ mice infused with Ang II (Ang II 4 weeks) or saline (Sham SA), respectively (*n =* 3 mice per group). Right, quantification of the indicated protein levels normalized to GAPDH. For C and G, immunofluorescence‐negative controls (NC) are shown in the corner. For D, F, H, and I, data represent mean ± SD. Data were first tested for normality using D'Agostino–Pearson (*n* > 8) or Shapiro–Wilk test (*n =* 3–8). Normal distribution data between two groups were performed by unpaired two‐tailed t‐tests (with homogeneity of variances tested) (H and I) or Welch's test (F). Nonparametric Mann‐Whitney tests were used for abnormal distributions (D). Ang II: angiotensin II; AAA: abdominal aortic aneurysm; TAA: thoracic aortic aneurysm; SA: suprarenal aorta; I: intima; M: media; Ad: adventitia; wks: weeks.

We examined CD34 expression profiles of aortic fibroblasts by extracting all fibroblasts from previously published single‐cell RNA sequencing (scRNA‐seq) datasets. These datasets included normal and aneurysmal aortas from both humans^[^ ^17^ ^]^ and mice, comprising Ang II‐induced suprarenal aortic aneurysms^[^ ^10^ ^]^ and CaCl_2_‐incubated infrarenal aortic aneurysms^[^ ^18^ ^]^ (Figure S1, Supporting Information). Feature plots showed high *Cd34* expression in a subcluster of aortic adventitial fibroblasts in both human and mouse AAA (**Figure** **1**A; Figure S1B, Supporting Information). The expression profile of *Cd34* was similar to that of *Pi16*, a recognized fibroblast progenitor marker gene.^[^ ^7a^ ^]^ Comparison of *Pi16* differentially expressed genes (DEGs; *Pi16* ^high^ vs *Pi16* ^low^) with *Cd34* DEGs (*Cd34* ^high^ vs *Cd34* ^low^) revealed a strong correlation between these two sets of DEGs. Importantly, the expression levels of other stem/progenitor marker genes, such as *Ly6a* ^[^ ^11a^ ^]^ and *Cd248* ^[^ ^19^ ^]^ were also highly associated with the expression levels of *Cd34* and *Pi16* in these fibroblast clusters (Figure 1B), suggesting that CD34^+^ cells may function as fibroblast progenitors in AAA.

To determine the distribution and expression patterns of CD34 in aortic tissues, we collected samples of human thoracic aortic aneurysm (TAA) and AAA, corresponding non‐dilated aortic tissues, and Ang II‐ and CaCl_2_‐induced mouse aneurysm samples. Histopathological analysis revealed that both human and mouse aneurysmal aortas showed marked SMC apoptosis, elastic lamina degradation, adventitial thickening, and fibrosis (Figure S2, Supporting Information). Immunofluorescence (IF) staining showed abundant CD34^+^ cells in the adventitia of human non‐dilated aortas, which were reduced in both human TAA and AAA aortas (Figure 1C). This observation was further confirmed by immunohistochemical (IHC) staining (Figure 1D; Figure S3A, Supporting Information). Western blotting revealed decreased levels of CD34 but increased levels of fibrosis‐related proteins, including Periostin, COL1A1, and vimentin, in human aneurysmal aortas (Figure 1E,F; Figure S3B,C, Supporting Information). A similar phenomenon was observed in Ang II‐ and CaCl_2_‐induced AAA. In particular, we observed abundant CD34^+^ cells in the abdominal aortic endothelium and adventitia of normal mice, but only a small number of CD34^+^ cells in Ang II or CaCl_2‐_induced AAA (Figure 1G,H; Figure S3D–F, Supporting Information). Western blotting revealed decreased CD34 expression and increased levels of fibrosis‐related proteins and platelet‐derived growth factor (PDGF) receptors in Ang II‐induced AAA (Figure 1I). Interestingly, the majority of CD34^+^ cells were positive for PDGFRa (Figure 1G,H) and PDGFRb (Figure S3E,F, Supporting Information) in the adventitia and the EC marker CD31 in the endothelium, but negative for the immune cell marker CD45 at baseline (Figure S3G,H, Supporting Information). Collectively, our data showed dynamic changes in CD34 expression and adventitial fibrosis in both human and mouse aortic aneurysms, implicating CD34^+^ cells in their pathogenesis.

### 2.2 CD34^+^ Cells Contribute to Myofibroblasts in AAA

**Figure 2:**
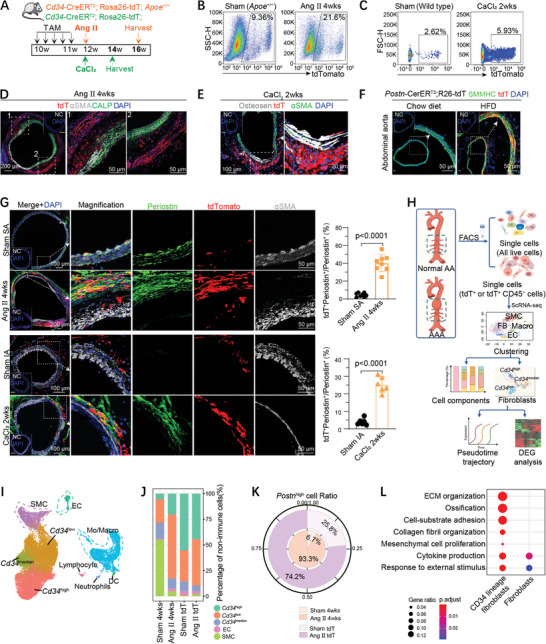
Lineage tracing and scRNA‐seq analysis revealed the contribution of CD34^+^ cells into myofibroblasts. A) Schematic diagram showing the experimental strategy of two murine AAA models induced by Ang II infusion (orange) and CaCl_2_ perivascular application (green), respectively. TAM, tamoxifen; W, weeks. B) Flow cytometric quantification comparing the percentage of tdTomato (tdT)^+^ cells in single nucleated live abdominal aortic cells isolated from *Cd34*‐CreER^T2^;R26‐tdT;*Apoe* ^−/−^ mice infused with Ang II (Ang II 4 weeks) or saline (Sham (*Apoe^−/−^*)) for 4 weeks (*n =* 4 mice per group). R26: Rosa26. C) Flow cytometric quantification comparing the percentage of tdT^+^ cells in single nucleated live abdominal aortic cells isolated from *Cd34*‐CreER^T2^;R26‐tdT mice on C57BL/6 background (wild type), 2 weeks after CaCl_2_ (0.5 m, CaCl_2_ 2 weeks) or PBS (Sham (Wild type)) treatment (*n =* 4 mice per group). D) IF staining for tdT and smooth muscle cell markers (CALP and αSMA) on aneurysmal suprarenal aorta section of *Cd34*‐CreER^T2^;R26‐tdT;*Apoe* ^−/−^ mice subjected to 4 weeks of Ang II infusion after TAM treatment. The box regions are magnified on the right with merged channels. CALP, Calponin. E) IF staining for tdT, αSMA, and calcium deposition marker (Osteosen) on aneurysmal infrarenal aorta section of *Cd34*‐CreER^T2^;R26‐tdT mice subjected to CaCl_2_ (0.5 m) incubation after TAM treatment. The box regions are magnified on the right with merged channels. F) IF staining for tdT and smooth muscle heavy chain (SMMHC) on aortic sections collected from *Postn*‐CreER^T2^;R26‐tdT mice fed with chow diet and high cholesterol diet (HFD) after TAM treatment, with magnification of the boxed region. G) IF staining for tdT, Periostin, and αSMA on Ang II‐induced aneurysmal (Ang II 4 weeks) and sham‐treated suprarenal aorta (Sham SA), and CaCl_2_‐induced aneurysmal (CaCl_2_ 2 weeks) and corresponding sham‐treated infrarenal aorta (Sham IA), collected from *Cd34*‐CreER^T2^;R26‐tdT;*Apoe* ^−/−^ (*n =* 8 mice per group) and *Cd34*‐CreER^T2^;R26‐tdT (*n =* 6 mice per group) mice, respectively, with magnification of the boxed regions and fluorescent quantification of the percentages of Periostin^+^tdT^+^ cells in Periostin^+^ cells on the right. H) Schematic representation of the experimental strategy and scRNA‐seq analysis. Whole abdominal aortas were digested for 10x scRNA‐seq. I) Visualization of unsupervised clustering in a Uniform Manifold Approximation and Projection (UMAP) plot of integrated 17151 single nucleated live cells extracted from our previously published scRNA‐seq datasets (GSE221789, whole abdominal aortic cells isolated from *Apoe* ^−/−^ mice infused with saline or Ang II for 4 weeks) and 17178 tdTomato^+^CD45^−^ cells isolated from abdominal aortas of *Cd34*‐CreER^T2^;R26‐tdT;*Apoe* ^−/−^ mice treated with sham and Ang II infusion for 4 weeks, respectively. J) Bar chart showing the proportion of major cell types across all the samples. Non‐immune aortic cells were isolated from *Apoe* ^−/−^ mice treated with sham (Sham 4 weeks) and Ang II (Ang II 4 weeks), and tdTomato^+^CD45^−^ cells were isolated from abdominal aortas of *Cd34*‐CreER^T2^;R26‐tdT;*Apoe* ^−/−^ mice infused with saline (Sham tdT) and Ang II (Ang II tdT) for 4 weeks, respectively. K) Pie chart displaying the ratios of *Postn* ^high^ cells across four groups. We defined fibroblasts with an expression level of rna_*Postn*>2 as *Postn* ^high^ cells. L) Bubble chart showing representative GO terms enriched in up‐regulated DEGs (differentially expressed genes) of CD34 lineage (CD34^+^ cell‐derived) fibroblasts (Ang II tdT vs Sham tdT) and total fibroblasts (Ang II 4 weeks vs Sham 4 weeks). Data were first tested and passed the normality test (D'Agostino‐Pearson or Shapiro–Wilk test) in G, and unpaired two‐tailed t‐tests (with homogeneity of variances tested) were performed. P values of each comparison were specified in the graph, *p <*0.05 was considered to be statistically significant. For D‐G, immunofluorescence‐negative controls (NC) are shown in the corner.

To elucidate the nature and cellular heterogeneity of abdominal aortic CD34^+^ cells during AAA progression, we used *Cd34*‐CreER^T2^; Rosa26‐tdT; (*Apoe* ^−/−^) mice to trace CD34^+^ cells and their progeny (**Figure** **2**A; Figure S4A, Supporting Information). As described in our previous report,^[^ ^13^ ^]^ 10‐week‐old mice were administered tamoxifen to induce tdTomato (tdT) labeling of CD34^+^ cells. Multiple experimental techniques, including whole‐body in vivo imaging, 3D reconstruction of the whole aorta, flow cytometric analyses, and IF staining, confirmed that the tdT signal accurately represented CD34^+^ cells (Figure S4B–D, Supporting Information). Moreover, IF staining and flow cytometry analysis revealed cellular heterogeneity of tdT^+^ cells in the abdominal aorta under physiological conditions, comprising abundant adventitial fibroblasts (37.1% PDGFRa^+^ fibroblasts), a subset of ECs (1.8% CD31^+^ ECs), perilipin A^+^ adipocytes (PLIN^+^), and a small population of immune cells (0.8% CD45^+^ cells) (Figure S4E–H, Supporting Information). Furthermore, a substantial proportion of PDGFRa^+^ fibroblasts (43.9%; Figure S4E, Supporting Information) and CD31^+^ ECs (24.8%; Figure S4F, Supporting Information) were positive for tdT^+^ cells, suggesting a significant contribution of CD34^+^ cells to these cell types in the abdominal aorta at baseline.

Two mouse AAA models were used to examine the role of CD34^+^ cells in AAA development: 1) adventitial application of calcium chloride (CaCl_2_) to the infrarenal aorta of *Cd34*‐CreER^T2^; Rosa26‐tdT mice and 2) subcutaneous implantation of an Ang II osmotic pump in *Cd34*‐CreER^T2^; Rosa26‐tdT; *Apoe* ^−/−^ mice to induce AAA, typically in the suprarenal aorta (Figure 2A). Male mice with an *Apoe^−/−^* background were used to increase the incidence of Ang II‐induced AAA, whereas both male and female wild‐type (WT) mice with a C57BL/6 background were used for CaCl_2_‐induced aneurysm. Flow cytometry analysis revealed that both Ang II infusion and CaCl_2_ adventitial application significantly increased the proportion of tdT^+^ cells in the abdominal aorta (Figure 2B,C). Consistent with a previous report,^[^ ^20^ ^]^ the proportion of CD34 lineage cells (tdT^+^) in the abdominal aorta of *Apoe* ^−/−^ mice (Figure 2B) was much higher than that in WT mice (Figure 2C) under physiological conditions. Moreover, IF staining revealed a substantial number of tdT^+^ cells accumulating in the thickened adventitia of both the suprarenal and infrarenal aneurysmal aortas, with few tdT^+^ cells in these aortas co‐expressing SMC markers (SM22, αSMA, Calponin, and Myh11; Figure 2D,E; Figure S5A, Supporting Information), suggesting a limited contribution of CD34^+^ cells to SMCs in AAA.

*Cd34*‐CreER^T2^;R26‐tdT;(*Apoe* ^−/−^) and *Postn‐Cre*ER^T2^;R26‐tdT mice were used to study the potential contribution of CD34^+^ cells to myofibroblasts in abdominal aorta homeostasis. IF staining of myofibroblast markers, including Periostin and αSMA,^[^ ^21^ ^]^ revealed that Periostin^+^ cells were scarce in normal healthy abdominal aorta. However, the number of Periostin ^+^ cells increased in vascular smooth muscle cells when the mice were fed a high‐cholesterol diet (HFD) or in the *Apoe^−/−^* background (Figure 2F,G, Figure S6A–C, Supporting Information). Interestingly, genetic lineage tracing studies using *Cd34*‐CreER^T2^, R26‐tdT, *Apoe* ^−/−^ mice have also revealed a limited contribution of CD34^+^ cells to Periostin^+^ cells in sham‐treated suprarenal or infrarenal aorta. However, some SMMHC^+^ cells were derived from Periostin^+^ cells in the abdominal aorta with an Apoe^−/−^ background before Ang II induction (Figure 2F, Figure S6A‐C, Supporting Information). In contrast, a large number of tdT^+^ (CD34^+^) cells accumulated in the adventitia and co‐expressed common fibroblast markers (PDGFRa, DDR2, Vimentin) (Figure S5B–D, Supporting Information) and myofibroblast markers (Periostin) (Figure 2G) upon Ang II or CaCl_2_ stimulation, demonstrating that CD34^+^ cells were activated to transdifferentiate into myofibroblasts during AAA progression. Quantitative analysis showed an increased contribution of CD34^+^ cells to myofibroblasts in both Ang II‐induced suprarenal aortic aneurysms and CaCl_2_‐induced infrarenal aortic aneurysms. In particular, we observed that ≈40.08 ± 9.42% of Periostin^+^ myofibroblasts in the suprarenal aortic aneurysm and 25.33 ± 4.41% in the infrarenal aortic aneurysm were derived from CD34^+^ cells (Figure 2G). Simultaneously, ≈74.92 ± 9.25% PDGFRa^+^ fibroblasts in the Ang II model and 60.08 ± 9.30% in the CaCl_2_ model originated from CD34^+^ cells (Figure S5C,D, Supporting Information). Interestingly, low expression of αSMA was occasionally observed in tdT^+^ cells in aortic samples with severe AAA (Figure S5C, Supporting Information), suggesting that these adventitial myofibroblasts acquire some contractile properties to enhance the biomechanical force and maintain vascular integrity when the SMC layer is severely damaged in advanced AAA.^[^ ^22^ ^]^ Additionally, we found that CD34 lineage cells contributed significantly to the inflammatory response, but had limited involvement in luminal and microvascular endothelial repair during the Ang II‐induced AAA process, comprising ≈32.21 ± 5.49% of CD45^+^ inflammatory cells in the Ang II model and 21.29 ± 5.62% in the CaCl_2_ model (Figure S7, Supporting Information), as well as 15.42 ± 3.62% of CD31^+^ endothelial cells in the Ang II model and 20.45 ± 4.26% in the CaCl_2_ model (Figure S8, Supporting Information). Collectively, these data confirmed the significant contribution of CD34^+^ cells to Periostin^+^ myofibroblasts in AAA.

To understand the precise identity and cellular heterogeneity of CD34 lineage cells (tdT^+^) at the single‐cell level, we conducted 10x scRNA‐seq analysis of single‐nucleated live tdT^+^/CD45^−^ cells isolated from normal (Sham tdT) and aneurysmal (Ang II tdT) abdominal aortas of *Cd34*‐CreER^T2^;R26‐tdT;*Apoe* ^−/−^ mice. Specifically, tdT^+^CD45^−^ cells in the Ang II tdT group were targeted for scRNA‐seq to capture more non‐immune CD34 lineage cells (Figure 2H; Figure S9A, Supporting Information). Following quality control, UMAP clustering, and preliminary cell‐type identification of tdT^+^ cells (Figure S9B,C, Supporting Information), they were integrated with whole‐cell datasets (GSE221789) of normal (Sham 4 weeks) and aneurysmal (Ang II 4 weeks) abdominal aortas to understand the cellular landscapes and communication between CD34 lineage cells and other aortic cells in AAA. The integrated datasets were re‐clustered and defined (Figure S9D,E, Supporting Information), revealing nine distinct cell types, including *Cd34* ^high^ fibroblasts, *Cd34* ^media^ fibroblasts, *Cd34* ^low^ fibroblasts, ECs, SMCs, Mo/Macro, neutrophils, DCs, and lymphocytes, in the normal and aneurysmal abdominal aortas (Figure 2I, Figure S9F–H, Supporting Information). CD34 lineage cells typically exhibited a similar cellular landscape with a whole aortic cell atlas except for the absence of SMCs (Figure S9I, Supporting Information). Consistent with immunostaining and flow cytometry analysis, cellular component analysis of non‐immune cells showed that fibroblasts constituted >90% of the tdT^+^ cells, with a decreased percentage of *Cd34* ^high^ fibroblasts and an increased proportion of *Cd34* ^low^ fibroblasts in the Ang II tdT group compared to those in the Sham tdT group (Figure 2J). Moreover, the Ang II (Ang II 4 weeks) group exhibited a significant decrease in SMC percentage, but a substantial increase in the proportion of fibroblasts compared with the Sham‐treated group (Figure 2J). Analysis of the fibroblast activation state, defined by high *Postn* expression, indicated a significantly high ratio of *Postn* ^high^ fibroblasts in both AAA groups (Ang II tdT and Ang II 4 weeks; Figure 2K), suggesting a key role for Periostin^+^ myofibroblasts in AAA development. Finally, Gene Ontology (GO) enrichment analysis indicated that under angiotensin II stimulation, CD34^+^ cell‐derived (CD34 lineage) fibroblasts exhibited enhanced extracellular matrix formation, calcification, proliferation, and migration capabilities compared to total fibroblasts (Figure 2L). Therefore, lineage tracing combined with scRNA‐seq analysis demonstrated that a substantial number of CD34^+^ cells contributed to Periostin^+^ myofibroblasts and CD45^+^ inflammatory cells in AAA, whereas a smaller subset of CD34^+^ cells generated ECs with minimal contribution to SMCs from CD34^+^ cells in murine AAAs.

### 2.3 Non‐BM‐Derived CD34^+^ Cells Function as Fibroblast Progenitor Cells

**Figure 3:**
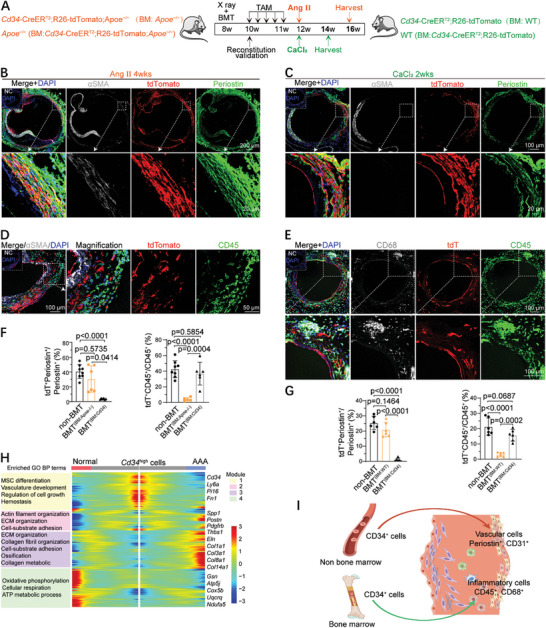
Non‐BM‐derived CD34^+^ cells function as fibroblast progenitor cells. A) Sketch of the experimental design for bone marrow transplantation (BMT), TAM injection, and Ang II infusion (orange) or CaCl_2_ incubation (green). B) IF staining for tdTomato, Periostin, and αSMA on aneurysmal suprarenal aortic sections of BMT*^Apoe^* ^−/−^→*^Cd34^* (BM: *Apoe* ^−/−^) chimeric mice subjected to 4 weeks of Ang II infusion. The box regions are magnified on the bottom with split channels. *Apoe* ^−/−^, *Apoe* knockout mice on C57BL/6 background; Cd34, *Cd34*‐CreER^T2^;R26‐tdT;*Apoe* ^−/−^ mice. C) IF staining for tdTomat, Poeriostin, and αSMA on aneurysmal infrarenal aorta sections of chimeric BMT^WT^→*^Cd34^* (BM: WT) mice stimulated by 0.5 m CaCl_2_. WT, wild‐type mice on C57BL/6 background; Cd34, *Cd34*‐CreER^T2^; R26‐tdT mice. D) Immunostaining for tdTomato and CD45 on aneurysmal suprarenal aortic sections of BMT*^Apoe^* ^−/−^→*^Cd34^* (BM: *Apoe* ^−/−^) chimeric mice. E) Immunostaining for tdTomato (tdT), CD68, and CD45 on aneurysmal infrarenal aortic sections of BMT^WT^→*^Cd34^* (BM: WT) chimeric mice stimulated by 0.5 m CaCl_2_. F) Quantification of the percentages of tdT^+^/Periostin^+^ cells in Periostin^+^ cells (left) or tdT^+^/CD45^+^ cells in CD45^+^ cells (right) from aneurysmal suprarenal aortic sections from non‐BMT mice and 2 types of chimeric mice (BMT^BM:^ *^Apoe^* ^−/−^, BMT^BM: CD34^) infused with Ang II for 4 weeks (*n =* 6–8 mice per group). G) Quantification of the percentages of tdT^+^/Periostin^+^ cells in Periostin^+^ cells (left) or tdT^+^/CD45^+^ cells in CD45^+^ cells (right) from aneurysmal infrarenal aortic sections from non‐BMT mice and 2 types of chimeric mice (BMT^BM: WT^, BMT^BM: CD34^). *n =* 6 mice per group. H) Heatmap of the top 100 significantly changed genes identified by the BEAM function from monocle in the branch point 1 as shown in Figure S11F (Supporting Information), and enriched GO biological process (BP) terms based on 4 gene co‐expression modules (left). I) Cartoon image showing the transdifferentiation fate of CD34^+^ cells of different origins in the context of AAA. For B‐E, NC are shown in the upper corner. For F and G, data represent mean ± SD and were first analyzed for normality test by Shapiro–Wilk test, and then assessed by ordinary 1‐way analysis of variance with Tukey test (equal SDs) or by Brown‐Forsythe and Welch analysis of variance tests with Tamhane's T2 test (unequal SDs).

To determine whether these CD34^+^ cells originated from BM or non‐BM sources, four types of chimeric mice were generated, as shown in **Figure** **3**A, in which BM cells from WT or *Apoe* ^−/−^ mice were transferred to irradiated CD34 lineage tracing mice (BMT^WT or^ *^Apoe^* ^−/−^→*^Cd34^*), and vice versa (BMT*^Cd34^* →^WT or^ *^Apoe^* ^−/−^). Flow cytometry confirmed successful BM reconstitution in the transplanted mice (Figure S10A, Supporting Information). In BMT*^Apoe^* ^−/−^→*^Cd34^* chimeric mice infused with Ang II, tdT^+^ cells were predominantly localized in the adventitia of the aneurysmal suprarenal aorta with a lamellar distribution (Figure 3B), and the majority of Periostin^+^ cells (30.62% ± 17.98%) were positive for tdTomato (Figure 3B,F). A similar phenomenon was observed in BMT^WT→^ *^Cd34^* chimeric mice treated with CaCl_2_ (Figure 3C,G). A small number of tdT^+^ cells were αSMA^+^ (Figure 3B), and very few CD45^+^ immune cells co‐expressed tdT (Figure 3D–G), suggesting that non‐BM CD34^+^ cells primarily contributed to myofibroblasts in Ang II‐ or CaCl_2_‐induced AAA, with no or limited contribution to CD45^+^ inflammatory cells. Furthermore, ≈20% of CD31^+^ ECs displayed tdT positivity (Figure S10B–D, Supporting Information). Conversely, in BMT*^Cd34^* ^→WT or^ *^Apoe^* ^−/−^ chimeric mice, a significant proportion of tdT^+^ cells exhibited CD45 expression, but rarely co‐expressed non‐immune cell markers, including CD31, SM22, Periostin, and αSMA, in the aneurysmal aortic wall (Figure S11A–C, Supporting Information). Quantitative analysis revealed a comparable level of tdT^+^ Periostin^+^ myofibroblasts between non‐BMT and BMT^(BM:^ *^Apoe^* ^−/−)^ mice, and a similar percentage of tdT^+^CD45^+^ immune cells between non‐BMT and BMT^(BM:^ *^Cd34^* ^)^ mice. Notably, a significant decrease in tdT^+^ Periostin^+^ myofibroblast levels and tdT^+^CD45^+^ immune cells was observed in BMT^(^ *^Cd34^* ^→WT or^ *^Apoe^* ^−/−)^ and BMT^(WT or^ *^Apoe^* ^−/−^→*^Cd34)^* mice, respectively (Figure 3F,G). Thus, BM‐derived CD34^+^ cells predominantly give rise to infiltrating CD45^+^ immune cells rather than vascular wall cells, including myofibroblasts.

These findings suggest that non‐BM CD34^+^ cells are activated and transdifferentiated into myofibroblasts during AAA progression. Next, pseudo‐time analysis with the Monocle R package revealed distinct trajectories after point 1 for fibroblasts from the normal (Sham 4 weeks and Sham tdT) and AAA groups (Ang II tdT and Ang II 4 weeks), where *Cd34* ^high^ cells initiated the trajectory and transitioned to *Cd34* ^low^ cells (Figure S11E–G, Supporting Information), suggesting potential transdifferentiation from CD34^+^ cells to myofibroblasts during AAA progression. Furthermore, gene expression profiling of pre‐ and post‐branch point 1 revealed four gene co‐expression modules (Figure 3H). Module 1, enriched with stem/progenitor genes, such as *Pi16, Ly6a*, and *Fn1*, was downregulated during the differentiation of *Cd34* ^high^ cells into myofibroblasts, with enriched GO biological processes related to mesenchymal stem differentiation and vascular development. Modules 2 and 3 primarily consisted of profibrogenic genes, such as *Postn, Pdgfrb, Spp1, Thbs1, Eln*, and *Col1a1*, which were upregulated during the transition from *Cd34* ^high^ cells to myofibroblasts and were consistently enriched for ECM and collagen organization, as well as biological functions related to cell adhesion and migration. Module 4 contained genes that were upregulated under oxidative stress and were primarily expressed in *Cd34* ^median^ cells (Figure 3H). Collectively, these results suggest that non‐BM CD34^+^ cells function as potential fibroblast progenitors that can be activated and transdifferentiated into myofibroblasts in AAA (Figure 3I).

### 2.4 CD34^+^ Cells Generated Periostin^+^ Myofibroblasts Forming Fibrous Collar to Protect AAA from Rupture

**Figure 4:**
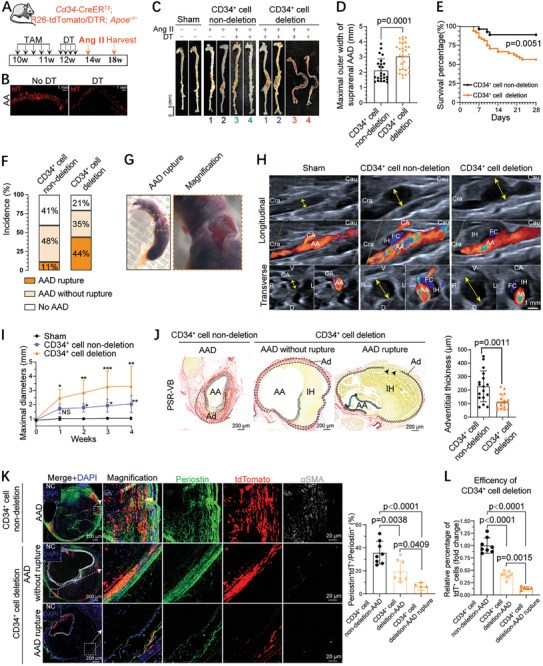
CD34^+^ cells generated Periostin^+^ myofibroblasts forming fibrous collars to protect AAAs from rupturing. A) Schematic diagram showing the experimental design for systemic CD34^+^ depletion in *Cd34*‐CreER^T2^;R26‐tdTomato/DTR;*Apoe* ^−/−^ mice. TAM, tamoxifen; W, weeks; Ang II, angiotensin II; DTR, diphtheria toxin receptor; DT, diphtheria toxin. B) 3D reconstruction of tdTomato (tdT)^+^ cell in abdominal aortas from mice with or without DT treatment. C) Representative gross images of the whole aortas were harvested from each mouse within the sham‐treated, CD34^+^ cell deletion group and non‐deletion group. Black numbers pointed to *Cd34*‐CreER^T2^;R26‐tdTomato/DTR;*Apoe* ^−/−^ mice treated with PBS. Green numbers pointed to *Cd34*‐CreER^T2^;R26‐tdT;*Apoe* ^−/−^ mice treated with DT. Blue numbers pointed to DT targeted *Cd34*‐CreER^T2^;R26‐tdT/DTR;*Apoe* ^−/−^ mice with suprarenal AAA without rupture, and red numbers pointed to the rupture one. D) Quantification of the maximal outer width of suprarenal AAA in CD34^+^ cell deletion group and non‐deletion group. All gross images of the whole aorta used for statistics are shown in Figure S12C–E (Supporting Information). E) Survival curve showing the percentages of survival mice in CD34^+^ cell deletion group and non‐deletion group after Ang II induction for 4 weeks. Data were tested by log‐rank (Mantel‐Cox) test. F) The incidence of Ang II‐induced AAA with or without rupture in the indicated mice during Ang II infusion. G) Image of a ruptured suprarenal AAA pinpointing the suprarenal rupture site. The box regions are magnified on the right. H) Transverse and longitudinal B‐Mode and Color Doppler ultrasound taken four weeks post‐Ang II induction, illustrating aortic wall enlargement and intramural hematoma (IH) formation in the indicated group. Blood flows toward the transducer in red, away from the transducer in blue. Cau, Caudal; Cra, Cranial; CA, celiac artery; FC, false channel; IH, intramural hematoma. I) Quantification of suprarenal abdominal aortic diameters by ultrasound images, with measurements taken weekly following angiotensin II induction in the indicated group (*n =* 6–8 mice per group). Gross images of the whole aortas for quantification are shown in Figure S12E (Supporting Information). J) Representative images of combined Picro‐Sirius red and Victoria Blue staining (PSR‐VB) showing aneurysmal suprarenal aortic sections of CD34^+^ cell deletion group with or without rupture, and non‐deletion group after Ang II induction for 4 weeks. PSR‐VB stain shows destruction of the elastic lamellae and remodeled fibrotic adventitia. Arrowheads indicate the rupture site of aortic adventitia absence of a fibrous collar. Right, quantification of adventitial thickness (*n =* 17–18 mice per group). Adventitial thickness was assessed and quantified by the thickness of the red fibrotic area in the adventitial layer of the aneurysmal region marked by the dotted line. AA: abdominal aorta; Ad: adventitia. K) IF staining for Periostin, tdTomato, and αSMA on suprarenal AAA sections of CD34^+^ cell deletion group and non‐deletion group after Ang II induction for 4 weeks. The box regions are magnified on the right with split channels. Quantification of the percentage of Periostin^+^tdT^+^ cells in Periostin^+^ cells. NC are shown in the upper corner. L) Quantification of the remaining tdT^+^ cells in AAA sections, illustrating the efficiency of CD34^+^ cell deletion in the indicated group. For D, I, J, K, and L, data were presented as mean ± SD and were first tested by Shapiro–Wilk test for normality. The Mann‐Whitney test was performed for D. The two‐way ANOVA test (Sidak's multiple comparison test) was used for I. Then unpaired t‐test with Welch's correction was performed for J. Ordinary one‐way analysis of variance with Tukey test (equal SDs) was accessed for K and L.

To functionally determine the role of CD34^+^ cells in AAA progression, Cd34‐CreER^T2^;R26‐tdT/DTR;*Apoe* ^−/−^ mice were generated and subjected to the experimental procedures illustrated in **Figure** **4**A, to selectively ablate the CD34^+^ population during Ang II‐induced AAA. 3D reconstruction and flow cytometry analysis demonstrated an obvious reduction in the number of tdT^+^ cells in the abdominal aorta, peripheral blood mononuclear cells, and BM (Figure 4B; Figure S12A,B, Supporting Information). To determine the potential cardiotoxic effects of diphtheria toxin (DT) and its impact on mouse survival during Ang II induction, *Cd34*‐CreER^T2^;R26‐tdT;*Apoe* ^−/−^ mice treated with DT and *Cd34*‐CreER^T2^;DTR/tdTomato; *Apoe* ^−/−^ mice treated with phosphate‐buffered saline (PBS) were used as separate controls for DT‐mediated cell ablation (Figure 4C; Figure S12C–E, Supporting Information). No significant differences were observed between the two control groups in terms of animal mortality or AAA development (Figure S12C–E, Supporting Information). Consequently, these experimental animals were combined into a single control (CD34^+^ cell non‐deletion) and CD34^+^ cell deletion groups for further analyses. Remarkably, the ablation of CD34^+^ cells aggravated AAA development, as evidenced by an increase in the maximal outer width of the suprarenal AAA and a significant decrease in the survival rate (Figure 4C–E). Furthermore, CD34^+^ cell‐deleted mice exhibited much higher AAA rupture and incidence rates, as autopsies of the deceased mice revealed substantial intra‐abdominal blood loss (Figure 4F; Figure S12F, Supporting Information). Careful dissection of the whole aortic tree determined the exact rupture site in the adventitia of the suprarenal AAA (Figure 4G). Importantly, longitudinal and transverse ultrasound images taken four weeks after Ang II induction revealed more pronounced aortic dilatation in CD34^+^ cell‐deleted mice, and color Doppler ultrasound revealed aggravated false channel and intramural hematoma (Figure 4H). The suprarenal aortic diameters in the CD34^+^ cell deletion group were considerably larger than those in the non‐deletion group after just one week of Ang II induction, exhibiting slow growth over the next three weeks (Figure 4I). Combined Picro‐Sirius red and Victoria blue (PSR‐VB) staining confirmed the destruction of the elastic membrane and remodeling of the fibrotic adventitia, revealing a thinner adventitial layer and lower degree of adventitial fibrosis in the CD34^+^ cell deletion group (Figure 4J). Notably, we observed that numerous tdT^+^ Periostin^+^ myofibroblasts were present in the adventitial layer of the aneurysm, forming a thick fibrous collar around the aneurysmal aorta in CD34^+^ cell non‐deletion mice. However, fewer tdT^+^ cells and tdT^+^ Periostin^+^ myofibroblasts were observed in the fibrous collar regions of the aneurysmal aorta in CD34^+^ cell deletion mice (Figure 4K). Histopathological analysis of the suprarenal aorta tissue from AAA rupture mice revealed almost complete disappearance of the aortic adventitial fibrous collar at the rupture site, with low levels of tdT^+^/Periostin^+^ myofibroblast investment (Figure 4J,K). Moreover, quantification of tdT^+^ cells confirmed that AAA rupture mice exhibited significantly higher CD34^+^ cell elimination efficiency than AAA‐unruptured mice (Figure 4L). Collectively, these findings suggest that the increased mortality in CD34^+^ cell‐depleted mice can be attributed to a weakened fibrous collar in the aortic adventitia. This weakening may potentially decrease the biomechanical strength and forces within the aortic wall, thereby increasing the likelihood of aneurysm rupture and hemorrhage. CD34^+^ cells actively contribute to the formation of a fibrous collar around the aneurysmal aorta by generating Periostin^+^ myofibroblasts, which may enhance the biomechanical strength to protect the aneurysm from rupture.

To further elucidate the specific role of CD34^+^ cells of different origins, chimeric mice, allowing for selective ablation of BM or non‐BM CD34^+^ cells, were used in the two mouse aneurysm models (Figure S13A,S14A, Supporting Information). We found that non‐BM CD34^+^ cell‐eliminated mice exhibited a higher incidence of Ang II‐induced AAA, more pronounced aortic dilatation, thinner adventitial layer, and fewer tdT^+^/Periostin^+^ myofibroblasts in the aneurysmal aorta than BMT‐CTRL mice (Figure S13, Supporting Information). However, after BM transplantation (BMT), the mice showed a reduced incidence of aneurysms in the Ang II‐induced AAA model,^[^ ^23^ ^]^ possibly explaining the lack of statistical difference in terms of mortality between the BMT‐DT and BMT‐CTRL groups (Figure S13D, Supporting Information). In contrast, the selective elimination of BM CD34^+^ cells had a minimal effect on the aortic expansion and adventitial thickness of Ang II‐induced suprarenal aortic aneurysms and CaCl_2_‐induced infrarenal aortic aneurysms (Figure S14, Supporting Information). This could be attributed to inflammatory cells from non‐CD34 lineages compensating for the loss of CD34^+^ cells, which was further supported by our co‐culture experiments involving BM cells and adventitial fibroblasts (Figure S15, Supporting Information). In summary, the deletion of systemic or non‐BM CD34^+^ cells, but not BM CD34^+^ cells, exacerbates AAA progression and increases animal mortality, supporting the beneficial effect of these CD34^+^ cells in restricting and stabilizing AAA.

**Figure 5:**
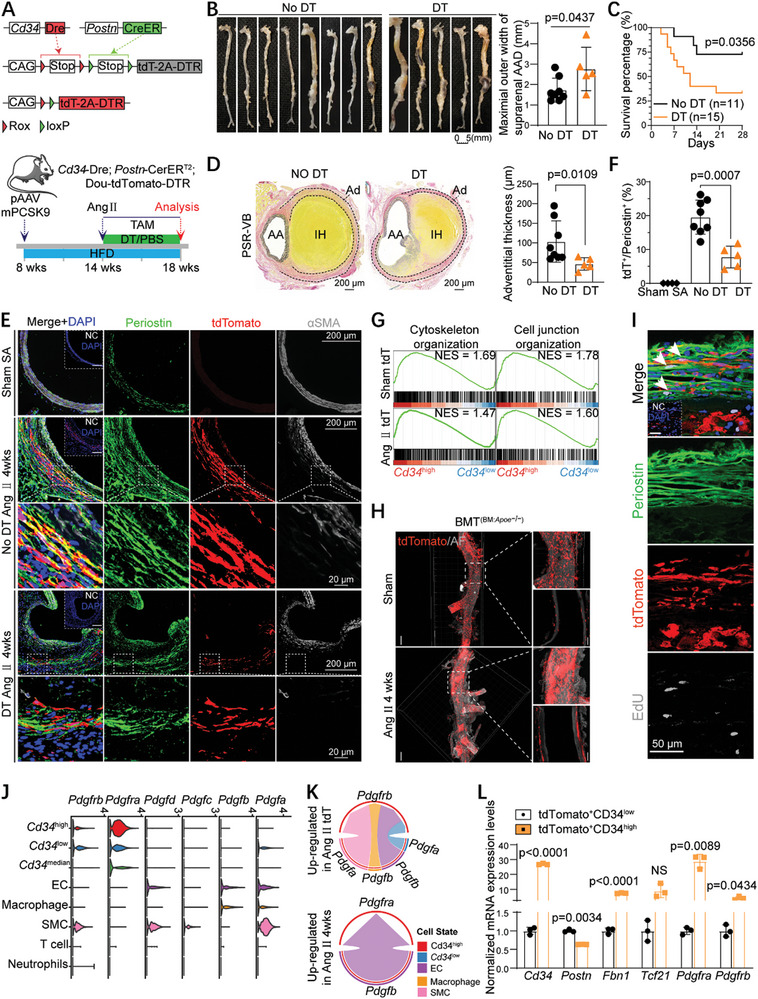
CD34^+^ cell activation through PDGF signaling. A) Schematic diagram showing the experimental design for genetic tracing and selective deletion of CD34^+^ cell‐derived Periostin^+^ myofibroblasts using dual recombinase‐based lineage tracing, as well as hypercholesterolemia and AAA induction in *Cd34*‐Dre;*Postn*‐CreER^T2^;Dou‐tdT‐DTR mice. HFD, high cholesterol diet; pAAV, plasmid adeno‐associated virus; mPCSK9, mouse proprotein convertase subtilisin/kexin type 9. B) Gross images of the whole aortas harvested from each mouse of CD34^+^Periostin^+^ cell deletion group (DT) and non‐deletion group (No DT) after Ang II induction for 4 weeks. Quantification (right) of the maximal outer width of suprarenal AAA displayed in the indicated group. C) Survival curve showing the percentages of survival mice in the indicated group after Ang II induction for 4 weeks. Data were tested by log‐rank (Mantel–Cox) test. D) PSR‐VB staining showing suprarenal aortic sections from indicated groups. Right, quantification of adventitial thickness. The thickness of the red fibrotic area in the adventitial layer of the aneurysmal region is marked by the dotted line. E) IF staining for tdTomato, Periostin, and αSMA in the normal suprarenal aorta (Sham SA) as well as aneurysmal suprarenal aortas of *Cd34*‐Dre;*Postn*‐CreER^T2^;Dou‐tdT‐DTR mice with or without DT injection. The boxed regions are magnified with split channels. F) Quantification of tdToamto (tdT)^+^ cells in Periostin^+^ cells in indicated groups. G) Gene Set Enrichment Analysis (GSEA) showing enrichment for cell migration‐related functions in *Cd34* ^high^ and *Cd34* ^low^ cells of *Cd34*‐CreER^T2^;R26‐tdT;*Apoe* ^−/−^ mice infused with saline (Sham tdT) and Ang II (Ang II tdT) for 4 weeks. NES: normalized enrichment score. H) 3D reconstruction of IF staining for tdTomato (red) and autofluorescence (grey, SMC layer) in abdominal aortas of chimeric BMT*^Apoe^* ^−/−^→*^Cd34^* mice (BMT^BM:^ *^Apoe−/−^*) infused with saline (Sham) or Ang II for 4 weeks, with magnification and vertical sections of the box region. Scale bars: 0.7 mm and 200 µm in magnification images. I) IF staining for proliferation marker EdU, tdTomato, and Periostin on aneurysmal suprarenal aortic sections from *Cd34*‐CreER^T2^;R26‐tdT;*Apoe* ^−/−^ mice with EdU injection. Arrowheads indicate tdT^+^EdU^+^ myofibroblasts. J) Violin plots showing the relative expression levels of PDGF ligands and PDGF receptors among all cell types. K) Chord diagrams showing the up‐regulated PDGF signaling received by *Cd34* ^high^ cells from the indicated cell groups in Ang II tdT and Ang II 4 weeks groups compared with corresponding controls. L) RT‐qPCR analysis of fibrosis‐related genes in flow cytometry sorted tdTomato^+^ *Cd34* ^low^ and tdTomato^+^ *Cd34* ^high^ subpopulations. Cells were isolated from adventitia of *Postn*‐CreER^T2^;R26‐tdT mice after TAM and Ang II induction. The flow cytometry gating strategy is shown in Figure S16H (Supporting Information). For E and I, NC are shown in the corner. For B, F, and L, data were presented as mean ± SD and were first tested by Shapiro–Wilk test for normality, and then an unpaired t‐test was performed. A nonparametric Mann–Whitney test was performed for D.

Next, an intersectional genetic strategy using dual recombinase‐based lineage tracing^[^ ^24^ ^]^ was employed to fate‐map the subpopulation of CD34^+^ cells (CD34^+^Periostin^+^ cells) in Ang II‐induced AAA and to investigate their potential contribution to AAA incidence and severity. *Cd34*‐Dre and *Postn*‐CreER^T2^ mice were crossbred with R26‐RSR‐LSL‐tdTomato‐DTR reporter (R26‐rox‐stop‐rox‐loxp‐stop‐loxp‐tdTomato‐DTR) mice to generate *Cd34*‐Dre;*Postn*‐CreER^T2^;Dou‐tdT‐DTR double‐lineage tracing and depleted mice (**Figure** **5**A). Eight‐week‐old male mice were administered a single intravenous pAAV/D377Y‐mPCSK9^[^ ^25^ ^]^ injection at a dosage of 1.0 × 10^11^ vector genomes, followed by HFD feeding to induce hypercholesterolemia (Figure S16A, Supporting Information) and increase the incidence of aortic aneurysm. Considering that Periostin is rarely expressed in adventitial fibroblasts under physiological conditions, 14‐week‐old mice were subcutaneously implanted with an Ang II osmotic pump after six weeks of HFD feeding, and tamoxifen was immediately administered after Ang II infusion to induce Cre‐loxp and Dre‐rox recombination. Moreover, DT injection allowed the specific deletion of CD34^+^ Periostin^+^ myofibroblasts in these mice (Figure 5A). The results indicated that the deletion of CD34^+^Periostin^+^ myofibroblasts significantly exacerbated Ang II‐induced AAA and reduced the survival rate and thickness of adventitial fibrous collars (Figure 5B–D). Compared to the DT group, a larger number of tdT^+^ cells co‐expressing periostin was detected in the adventitia of the aneurysmal suprarenal aorta in the No‐DT group. Notably, these CD34^+^Periostin^+^ myofibroblasts were predominantly distributed in regions where SMCs were absent (Figure 5E,F). No tdT^+^ cells were observed in the adventitia of the abdominal aorta from mice without Ang II infusion, and very few tdT^+^ cells were co‐expressed with αSMA (Figure 5E). Semi‐quantitative analysis revealed that CD34^+^Periostin^+^ myofibroblasts accounted for 19.55% ± 5.03% of Periostin^+^ myofibroblasts (Figure 5F). Because dual recombinase‐mediated lineage tracing depends on both Cre‐loxp and Dre‐rox recombination, we expected a relatively lower level of dual recombination efficiency than that of single recombination. However, we observed a relatively small number of tdT^+^ cells labeled with *Cd34*‐Dre and *Postn*‐CreER^T2^ (Figure 5F) compared to those labeled with *Cd34*‐CreER^T2^ tracer and Periostin immunostaining (Figure 2G). Therefore, we demonstrated that adventitial CD34^+^ cells can be activated and transdifferentiated into Periostin^+^ myofibroblasts during AAA progression, which has a beneficial effect on restricting and stabilizing AAA, thereby protecting animals from death.

### 2.5 CD34^+^ Cells Activation through PDGF Signaling

An in‐depth analysis using scRNA‐seq data from the Sham tdT and Ang II tdT groups revealed the potential mechanisms underlying CD34^+^ cell activation and transdifferentiation. Gene Set Enrichment Analysis indicated that *Cd34* ^high^ cells exhibited a strong cell migration ability with enriched pathways for cytoskeleton organization, cell junction organization, cell spreading, and cell differentiation (Figure 5G; Figure S16B, Supporting Information), whereas *Cd34* ^low^ cells exhibited pathways associated with cell activation and ossification (Figure S16B, Supporting Information). 3D imaging of whole aortas from BMT^(BM^ *^: Apoe−/−^* ^)^ chimeric mice revealed increased CD34^+^ cell migration to vulnerable sites, where they clustered and self‐proliferated (Figure 5H). Additionally, IF staining for tdTomato and the cell proliferation marker Ki67 confirmed the proliferation of CD34^+^ cells in the abdominal aneurysmal aortas (Figure S16C, Supporting Information), which was further supported by a 5‐ethynyl‐2′‐deoxyuridine (EdU) incorporation assay (Figure S16D, Supporting Information). EdU‐incorporated myofibroblasts (tdT^+^ Periostin^+^) originating from CD34^+^ cells were also detected (Figure 5I). Thus, upon Ang II stimulation, some CD34^+^ cells, potentially CD34^+^ fibroblast progenitors, can be activated and migrate to vulnerable sites where they proliferate and transdifferentiate into myofibroblasts, thereby protecting AAA from rupture.

Subsequently, we used the Cellchat R package to analyze cellular communication and identify the signaling pathways that activate CD34^+^ cells, observing abundant PDGF signaling between fibroblasts and other cell types (Figure 5J; Figure S16E, Supporting Information). Specifically, PDGF receptors were predominant in fibroblasts, with *Pdgfra* in the *Cd34* ^high^ subpopulation and *Pdgfrb* in both *Cd34* ^high^ and *Cd34* ^low^ cells, as well as in SMCs (Figure 5J). IF staining and 3D reconstruction confirmed the co‐expression of tdT and PDGFRa, particularly at the dilated sites of the abdominal aneurysmal aortas (Figure S16F, Supporting Information). Furthermore, PDGF ligand‐receptor pair analysis revealed that *Cd34* ^high^ could receive all PDGF signals from SMCs, ECs, and macrophages, with enhanced interactions in AAA (Figure S16G, Supporting Information). Notably, we observed significant upregulation in the expression levels of *Pdgfrb* and *Pdgfra* in Ang II tdT and Ang II 4 weeks *Cd34* ^high^ cells (Figure 5K). Additionally, RT‐qPCR demonstrated increased expression of fibrotic genes, including *Pdgfrb* and *Pdgfra*, in tdT^+^CD34^high^ cells compared to tdT^+^CD34^low^ cells isolated from *Postn*‐CreER^T2^;tdT‐R26 mice with TAM and Ang II induction (Figure 5L; Figure S16H, Supporting Information). Collectively, our results indicate that PDGF ligands secreted by other aortic cells, including SMCs, ECs, and macrophages, trigger PDGF signaling pathways by binding platelet‐derived growth factor receptors (PDGFRs) on CD34^+^ cells to activate CD34^+^ cells, thereby promoting CD34^+^ cell migration and transdifferentiation into myofibroblasts during AAA pathology.

### 2.6 Specific Deletion PDGFRb Rather Than PDGFRa in CD34^+^ Cells Significantly Increased Mortality of AAA

**Figure 6:**
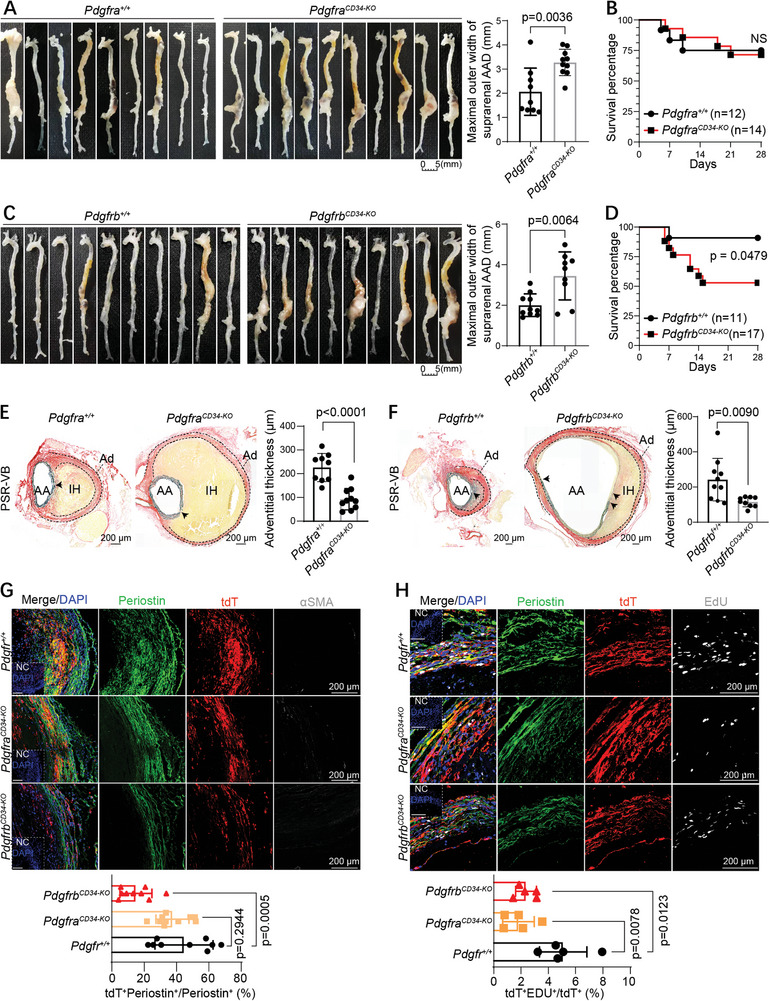
Specific deletion of PDGFRb rather than PDGFRa in CD34^+^ cells significantly increased the mortality of AAA. A) Gross images of the whole aortas harvested from each mouse infused with Ang II for 4 weeks after TAM induction in *Cd34*‐CreER^T2^;R26‐tdT;*Pdgfra^+/+^*;*Apoe* ^−/−^ (*Pdgfra^+/+^*) and *Cd34*‐CreER^T2^;*Pdgfra^fl/fl^*;R26‐tdT;*Apoe* ^−/−^ (*Pdgfra* ^CD34‐KO^) groups. Quantification (right) of the maximal outer width of suprarenal AAA of each mouse from indicated groups. B) Survival curve showing the percentages of survival mice after Ang II induction for 4 weeks in the indicated group. C) Gross images of the whole aortas harvested from each mouse infused with Ang II for 4 weeks after TAM induction in *Cd34*‐CreER^T2^;R26‐tdT;*Pdgfrb^+/+^*;*Apoe* ^−/−^ (*Pdgfrb^+/+^*) and *Cd34*‐CreER^T2^;*Pdgfrb* ^fl/fl^;R26‐tdT;*Apoe* ^−/−^ (*Pdgfrb* ^CD34‐KO^) groups. Quantification (right) of the maximal outer width of suprarenal AAA of each mouse from indicated groups. D) Survival curve showing the percentages of survival mice after Ang II induction for 4 weeks in the indicated group. E) Representative images showing PSR‐VB staining of suprarenal AAA sections of *Pdgfra* ^CD34‐KO^ and *Pdgfra^+/+^* mice after Ang II induction for 4 weeks. Adventitial thickness (right) was assessed and quantified by the red fibrotic area in the adventitial of the AAA region marked the dotted line (*n =* 9–10 mice per group). F) Representative PSR‐VB staining images of suprarenal AAA sections of *Pdgfrb* ^CD34‐KO^ and *Pdgfrb^+/+^* mice after Ang II induction for 4 weeks. Adventitial thickness (right) was assessed and quantified (*n =* 9–10 mice per group). Arrowheads in E and F indicate disruption of elastic membrane. G) IF staining for Periostin, tdTomato (tdT), and αSMA on aneurysmal aortic sections of *Pdgfra* ^CD34‐KO^, *Pdgfrb* ^CD34‐KO^, and *Pdgfr^+/+^* (*Cd34*‐CreER^T2^;R26‐tdT;*Apoe* ^−/−^) mice. Quantification (bottom) of the percentage of tdT^+^Periostin^+^ cells in Periostin^+^ cells for the indicated group. H) IF staining for Periostin, tdTomato (tdT), and EdU on aneurysmal aortic sections of *Pdgfra* ^CD34‐KO^, *Pdgfrb* ^CD34‐KO^ and *Pdgfr^+/+^* mice. Quantification (bottom) of the percentage of tdT^+^EdU^+^ cells in tdT^+^ cells for the indicated group. For G and H, NC are shown in the corner. For A, C, E, F, G, and H, quantitative data represent mean ± SD; Data were first tested and passed the normality test (Shapiro–Wilk test), followed by unpaired two‐tailed t‐tests (with homogeneity of variances tested, A, C, E, F) or ordinary 1‐way analysis of variance with Tukey test (G, H). For B and D, data were tested by log‐rank (Mantel‐Cox) test. P values of each comparison were specified in the graph, *p <*0.05 was considered to be statistically significant.

To further validate the function of PDGFRs during AAA progression, we crossed Cd34‐CreER^T2^;R26‐tdTomato;*Apoe* ^−/−^ with *Pdgfra* ^fl/fl^ or *Pdgfrb* ^fl/fl^ mice to generate Cd34‐CreER^T2^;*Pdgfr* ^fl/fl^;R26‐tdTomato;*Apoe* ^−/−^ mice to knockout PDGFRa or PDGFRb specifically in CD34^+^ cells (Figure S17A, Supporting Information) and used the *Cd34*‐CreER^T2^;R26‐tdTomato;*Apoe* ^−/−^ mice as control. Western blotting confirmed the specific PDGFR deletion in CD34^+^ cells isolated from the respective mice (Figure S17B,C, Supporting Information). CD34^+^ cell‐specific knockout of PDGFRa exacerbated AAA development but did not elevate animal mortality, as evidenced by the increased maximum diameters of aortas in the *Pdgfra* ^−/−^ group compared with controls, and a comparable survival rate was observed between the two groups (**Figure** **6**A,B). Notably, PDGFRb deletion in CD34^+^ cells not only promoted AAA progression but also significantly increased the mortality rate of aneurysms (Figure 6C,D). PSR‐VB staining showed a significant decrease in the adventitial thickness of abdominal aneurysmal aortas in both *Pdgfra* ^fl/fl^ and *Pdgfrb* ^fl/fl^ mice compared to that in the controls (Figure 6E,F). We also observed a significant reduction in tdT^+^ Periostin^+^ myofibroblasts in the adventitial fibrous collar regions in *Pdgfrb* ^fl/fl^ mice, but not in *Pdgfra* ^fl/fl^ mice (Figure 6G). Additionally, tdT^+^αSMA^+^ myofibroblasts were scarcely detected in the adventitial fibrous collar regions in all mice (Figure 6G). Finally, IF staining for EdU, tdT, and Periostin demonstrated that a specific knockout of either PDGFRa or PDGFRb in CD34^+^ cells significantly decreased CD34^+^ cell proliferation in the pathological status of AAA (Figure 6H). Collectively, although both PDGFRs in CD34^+^ cells play important roles in CD34^+^ cell proliferation and AAA progression, PDGFRb, rather than PDGFRa, is required for CD34^+^ cell transdifferentiation into Periostin^+^ myofibroblasts, thereby protecting aneurysms from rupture.

### 2.7 PDGF‐PDGFRb‐PI3K Axis is Responsible for CD34^+^ Cell Activation and Transdifferentiation to Periostin^+^ Myofibroblasts

**Figure 7:**
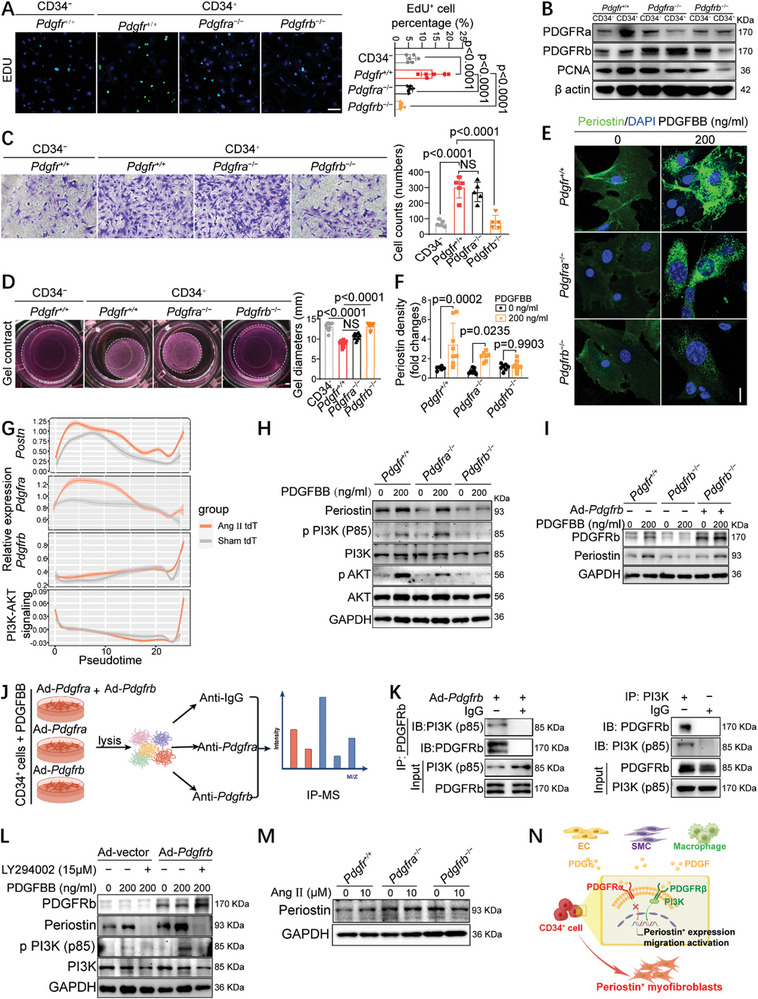
PDGF‐PDGFRb‐PI3K signaling axis is required for CD34^+^ cells transdifferentiation into myofibroblasts. A) EdU assay showing cellular proliferation. Aortic adventitial CD34^−^ and CD34^+^ cells were isolated from *Cd34*‐CreER^T2^;R26‐tdT;*Apoe* ^−/−^ mice with (*Pdgfra^−/−^* and *Pdgfrb^−/−^*) or without (*Pdgfr^+/+^*) deletion of PDGFRs in CD34^+^ cells, followed by PDGFBB (200 ng mL^−1^) stimulation, scale bar:100 µm. Dotplot (right) displaying the percentages of EdU^+^ cells in primary adventitial cells across different groups (*n =* 6 independent experiments of 5 fields each per group). B) Western blot showing the expression of cell proliferation marker PCNA and the specific knockout efficiency of PDGFRa and PDGFRb in the indicated groups. β actin served as a loading control. C) Representative transwell image showing cellular migration capacity of indicated cells in response to PDGFBB (200 ng mL^−1^). Scale bar: 100 µm. Quantification (right) of cell counts in indicated groups (*n =* 5 independent experiments of 5 fields per group). D) Representative photographs of collagen gels taken at 12 h in indicated cells after PDGFBB stimulation (*n =* 10 independent experiments). Scale bar: 1mm. Quantification of gel diameters in indicated groups (right). E) Representative IF staining images for Periostin in indicated cells upon PDGFBB (200 ng mL^−1^) or PBS stimulation for 12 h. Scale bar: 25 µm. F) Quantification of Periostin density (fold change) in the indicated group (*n =* 7–9 wells per group). G) Curve diagrams displaying the relative expression levels of indicated genes or signaling pathways in indicated CD34 lineage cells (Sham tdT and Ang II tdT group) along pseudotime differentiation trajectory. H) Western blot analysis showing protein expressions in indicated cells after PDGFBB stimulation for 12 h. I) Immunoblotting analysis of Periostin expressions in indicated cells with (Ad‐*Pdgfrb*) or without PDGFRb overexpression for 48 h, followed by PDGFBB (200ng mL^−1^) or PBS treatment for 12 h. J) Schematic diagram showing the coimmunoprecipitation (Co‐IP) and mass spectrometry analysis to identify the specific interacting targets of PDGFRa and PDGFRb. K) Left, Co‐IP analysis of the interaction of PDGFRb and PI3K in CD34^+^ cells infected with adenovirus expressing *Pdgfrb* (Ad‐*Pdgfrb*) and treated with PDGFBB (200ng mL^−1^) for 12 h. The protein complex was pulled down using anti‐IgG or anti‐PDGFRb antibody, and detected by antibodies against PI3K (p85) and PDGFRb, respectively. Right, reciprocal co‐IP analysis with anti‐PI3K antibody (for IP) to detect endogenous interaction between PI3K and PDGFRb. L) Western blot analysis of Periostin, total and phosphorylated PI3K in *Pdgfrb* ^−/−^ cells infected with Ad‐vector and Ad‐*Pdgfrb* and treated with PDGFBB (200ng mL^−1^) and/or LY294002 (15 µm) as indicated for 12 h. M) Immunoblotting analysis of Periostin in indicated cells upon Ang II (10 µm) or PBS stimulation for 12 h. N) Schematic diagram summarized the molecular mechanism of CD34^+^ cells activation and transdifferentiation into myofibroblasts. For A, C, and D, quantitative data represent mean ± SD; Data were first tested and passed the normality test (Shapiro–Wilk test), and then ordinary one‐way ANOVA analysis (Tukey's multiple comparisons test) were performed. For F, data were tested by a two‐way ANOVA test (Sidak's multiple comparison test). For B, H, I, L, and M, quantifications of the indicated protein levels normalized to the loading control were shown in Figure S18 (Supporting Information) (*n =* 3 independent experiments). *P <*0.05 was considered to be statistically significant.

To further investigate the molecular mechanism of CD34^+^ cell activation in vitro, we used PDGFBB, a ligand that can activate both PDGFRa and PDGFRb,^[^ ^26^ ^]^ to stimulate CD34^+^ cells. CD34^+^ cells were isolated from the aortas of *Cd34*‐CreER^T2^,R26‐tdTomato,*Apoe* ^−/−^; *Cd34*‐CreER^T2^, *Pdgfra* ^fl/fl^, R26‐tdTomato, *Apoe* ^−/−^; and *Cd34*‐CreER^T2^;*Pdgfrb* ^fl/fl^;R26‐tdTomato;*Apoe* ^−/−^ mice after TAM induction, followed by various functional studies. The EdU assay showed that CD34^+^ cells exhibited a stronger proliferative ability than CD34^−^ cells, requiring both PDGFRa and PDGFRb for PDGFBB‐induced proliferation (**Figure** **7**A). Consistently, western blotting revealed higher expression of the cellular proliferative marker proliferating cell nuclear antigen (PCNA) in *Pdgfr* ^+/+^CD34^+^ cells than in *Pdgfr* ^+/+^CD34^−^ cells, with a significant decrease observed in both *Pdgfra* ^−/−^ CD34^+^ and *Pdgfrb* ^−/−^ CD34^+^ cells (Figure 7B; Figure S18A, Supporting Information). The Transwell experiment demonstrated higher migration ability in CD34^+^ cells, which was attenuated by *Pdgfrb* knockout rather than *Pdgfra* knockout (Figure 7C). A collagen gel contraction assay was conducted to examine the effect of PDGFRs on the ECM remodeling capability of fibroblasts, a key function of myofibroblasts.^[^ ^27^ ^]^ An increased level of ECM contraction induced by PDGFBB was observed in CD34^+^ fibroblasts, which was mitigated by the deletion of *Pdgfrb* rather than *Pdgfra* (Figure 7D). IF staining revealed that PDGFBB stimulation substantially increased Periostin expression in *Pdgfr* ^+/+^ and *Pdgfra* ^−/−^ cells, but not in *Pdgfrb* ^−/−^ fibroblasts (Figure 7E,F), suggesting a critical role for PDGFRb in PDGFBB‐induced Periostin expression. Moreover, no consistent synergistic effect on the proliferation, migration, and transdifferentiation of CD34^+^ cells was observed following the simultaneous knockdown of *Pdgfra* and *Pdgfrb* by small interfering RNA (siRNA) (Figure S19, Supporting Information). Collectively, these data demonstrated that PDGFRb plays a more pivotal role than PDGFRa in CD34^+^ cell activation and transdifferentiation into Periostin^+^ myofibroblasts.

To further identify the downstream signaling pathways of PDGFRb, we analyzed CD34 lineages and observed dynamic changes in fibrosis‐related genes and several other signaling pathways during the pseudo‐time transitions. Specifically, *Postn*, *Pdgfra*, and *Pdgfrb* were consistently upregulated in the AAA group compared to those in the Sham‐treated group (Figure 7G). Notably, *Postn* expression was predominantly upregulated in CD34^+^ cells at both the early and late stages of differentiation trajectories, whereas *Pdgfra* and *Pdgfrb* expression substantially increased at the prophase and end of differentiation trajectories (Figure 7G), suggesting a role for PDGFRa and PDGFRb in the early and late stages of the CD34^+^ cell transdifferentiation process, respectively. Among all the screened pathways, the changing trend of the PI3K‐AKT signaling pathway was almost identical to that of *Pdgfrb* in the context of AAA (Figure 7G; Figure S20A, Supporting Information). Therefore, we performed western blotting to verify the elevated Periostin levels and activation of the PI3K‐AKT pathway in control CD34^+^ cells after PDGFBB administration (Figure 7H; Figure S18B, Supporting Information). Notably, the knockout of PDGFRb, rather than PDGFRa, in CD34^+^ cells, abolished the upregulation of Periostin, p‐PI3K, and p‐AKT induced by PDGFBB (Figure 7H and S18B, Supporting Information), suggesting a critical role for PDGFRb in these processes. We then constructed an adenovirus expressing *Pdgfra* (Ad‐*Pdgfra*), *Pdgfrb* (Ad‐*Pdgfrb*), and an Ad‐vector to infect adventitial CD34^+^ cells, achieving high overexpression levels with an infection titer of 10^8^PFU mL^−1^ (Figure S20B, Supporting Information). Overexpression of PDGFRb in *Pdgfrb* ^−/−^ cells rescued the Periostin expression induced by PDGFBB (Figure 7I; Figure S18C, Supporting Information). Furthermore, immunoprecipitation mass spectrometry (Figure 7J) identified PI3K as a downstream target of PDGFRb, but not PDGFRa (Supplementary Excel 2). Exogenous and reciprocal endogenous co‐immunoprecipitation experiments demonstrated the interaction between PDGFRb and PI3K in CD34^+^ cells treated with PDGFBB (Figure 7K), whereas no interaction was observed between PDGFRa and PI3K (Figure S20C, Supporting Information), which may account for the functional disparity between PDGFRa and PDGFRb, as previously described. Additionally, Periostin expression was almost entirely inhibited by the PI3K pathway inhibitor LY294002 in *Pdgfrb* ^−/−^ CD34^+^ cells with or without PDGFRb overexpression (Figure 7L; Figure S18D, Supporting Information), confirming the crucial role of the PI3K signaling pathway in regulating Periostin expression in CD34^+^ cells. Additionally, we observed that Ang II marginally elevated Periostin expression in CD34^+^ cells, and that neither PDGFRb‐ nor PDGFRa‐deletion affected Ang II‐induced Periostin expression in these cells (Figure 7M; Figure S18E, Supporting Information). Therefore, the PDGF‐PDGFRb‐PI3K axis is responsible for the activation and transdifferentiation of CD34^+^ cells into Periostin^+^ myofibroblasts (Figure 7N).

## 3 Discussion

Using scRNA‐seq and lineage tracing techniques, we previously reported that CD34^+^ cells constitute a heterogeneous population, primarily composed of a large number of adventitial fibroblasts, a small fraction of endothelial progenitors, and immune cells.^[^ ^13^, ^14^ ^]^ However, the potential role of CD34^+^ cells in adventitial remodeling in AAA remains unclear. In this study, we found that non‐BM‐derived CD34^+^ cells function as fibroblast progenitor cells to generate Periostin^+^ myofibroblasts, which participate in the formation of the adventitial fibrous collar of the aneurysmal aorta, thereby restricting aneurysm development. The significant downregulation of CD34 expression in both human and mouse aneurysmal tissues, in combination with the increased expression of fibrosis‐ and myofibroblast‐related markers, including PDGFRb and Periostin, suggested that CD34^+^ cells transition into an activated fibroblast state distinct from that of common fibroblasts. Corroborating this progenitor role, scRNA trajectory analysis reveals that *Cd34* ^high^ cells are in an undifferentiated primitive state, with marked enrichment in mesenchymal stem cell differentiation pathways. This profile highlights the stem‐like properties of adventitial CD34^+^ cells and their capacity to differentiate in response to aneurysm signals. Functionally, our combined BMT and DTR mouse model demonstrates that the depletion of either systemic or non‐BM CD34^+^ cells exacerbates aneurysm progression, elucidating their essential protective role. Notably, the specific elimination of CD34^+^ cell‐derived Periostin^+^ myofibroblasts in dual recombinase‐based lineage tracing (*Cd34*‐Dre;*Postn*‐CreER^T2^;Dou‐tdT‐DTR) mice further confirms the functional contribution of CD34^+^ cell‐derived Periostin^+^ myofibroblasts to adventitial fibrous collar formation within aortic aneurysms, and their beneficial effects in preventing abdominal aortic rupture and increasing animal survival. Mechanistically, PDGFRb is required for CD34^+^ cell activation and transdifferentiation into Periostin^+^ myofibroblasts in AAA. Therefore, our study highlights CD34^+^ cells as potential therapeutic targets for decelerating AAA progression and enhancing aneurysmal aortic stability.

Rupture and hemorrhage of an aortic aneurysm constitute a complex process influenced by intricate interactions among multiple factors. Over time, a progressive imbalance between connective tissue repair and degradation drives aneurysm growth,^[^ ^9^ ^]^ ultimately leading to aortic wall rupture when the structural integrity can no longer withstand the applied stresses.^[^ ^28^, ^29^ ^]^ Studies have demonstrated that ECM degradation and abnormal collagen fibril formation play crucial roles in AAA occurrence and progression.^[^ ^30^ ^]^ Fibroblasts adopt a transient and contractile myofibroblast (αSMA^+^ myofibroblasts) or ECM‐producing (Periostin^+^ myofibroblasts) phenotype in adventitial remodeling in response to damage.^[^ ^8^, ^9^ ^]^ Thus, phenotypic switching of fibroblasts into myofibroblasts and deposition of collagen and other ECM molecules are essential for maintaining the compromised structural integrity of the AAA wall.^[^ ^5^, ^29^ ^]^ In particular, it has been reported that peri‐adventitial transplantation of gingival fibroblasts onto elastase‐injured abdominal aortas^[^ ^31^ ^]^ or intravenous injection of BM‐derived fibrocytes into mice infused with Ang II^[^ ^32^ ^]^ effectively impedes the progression and rupture of AAA, suggesting the therapeutic potential of fibroblast/fibrocyte transplantation in AAA. Consistent with these previous findings, our study demonstrated that non‐BM‐derived CD34^+^ cells can create an activated fibrotic microenvironment to promote the repair of damaged/disrupted adventitial connective tissues induced by AAA pathological conditions and may enhance the biomechanical strength of aneurysmal aortas, thereby limiting AAA progression and protecting them from rupture.

The contribution of total and non‐BM CD34^+^ cells to endothelial repair in AAA appears to be limited, as indicated by the small and comparable percentage of CD34^+^ cell‐derived ECs in the aortic walls under both pathological and physiological conditions. However, our previous study reported that a small proportion of non‐BM‐derived CD34^+^ cells functioned as endothelial progenitor cells and participated in endothelial regeneration in a wire injury‐induced endothelial denudation mouse femoral artery model.^[^ ^13^ ^]^ This discrepancy may be due to the fact that endothelial dysfunction is an early pathologic event in AAA formation, leading to both oxidative stress and inflammatory cell infiltration into the degenerating aortic wall.^[^ ^2^, ^33^ ^]^ In contrast, wire injury‐induced endothelial denudation results in more severe endothelial damage and complete EC loss in the injured area, necessitating rapid re‐endothelialization from various cellular sources, including CD34^+^ endothelial progenitor cells. Thus, we hypothesized that the contribution of CD34^+^ cells to endothelial regeneration may be dependent on the severity of endothelial damage. Additionally, our data showed that selectively deleting CD34^+^ cells of BM origin did not affect adventitial fibrous collar thickness or AAA severity. This can be attributed to several factors. First, a significant number of non‐CD34^+^ cell‐derived inflammatory cells were observed, potentially compensating for the loss of CD34^+^ cell‐derived inflammatory cells during AAA pathogenesis. Second, DT injection effectively reduced, but did not completely eliminate, all CD34^+^ cells from the experimental mice, with the remaining CD34^+^ cells possibly generating inflammatory cells and contributing to AAA development. Third, CD34^+^ cells targeted for depletion are those induced by TAM within a specific time window, and newborn CD34^+^ cells may be generated after TAM withdrawal at a later stage.

The PDGF signaling pathway is crucial for the self‐renewal and proliferation of fibroblasts. PDGF ligands bind to PDGFRa and PDGFRb tyrosine kinase receptors, which activate multiple downstream signaling cascades, including phosphatidylinositol 3 kinase/protein kinase B pathway and mitogen‐activated protein kinase/extracellular signal‐regulated kinase pathway. A previous study showed that overexpression or inhibition of PDGF significantly affects fibrosis in the BM, lung, kidney, liver, and heart of mice.^[^ ^34^ ^]^ PDGF promotes tissue fibrosis by regulating cell division, proliferation, or chemotactic properties of tissue fibroblasts.^[^ ^35^ ^]^ Nintedanib, a clinically approved antifibrotic agent, is a tyrosine kinase inhibitor that blocks PDGFR activity and inhibits the differentiation of fibroblasts into myofibroblasts and their proliferation in patients with idiopathic pulmonary fibrosis.^[^ ^36^ ^]^ Clinical studies have reported a close link between *Pdgfrb* variants and aneurysms^[^ ^37^ ^]^ and a strong association of nintedanib treatment with an increased risk of aortic aneurysm and dissection,^[^ ^38^ ^]^ thereby providing clinical relevance to pharmacological interventions or targeting fibroblasts in patients with AAA. Cellular communication analysis using scRNA‐seq datasets revealed intensive PDGF‐PDGFR interactions between CD34^+^ cells and other aortic cells during AAA development, with upregulated PDGFRb expression in CD34^+^ fibroblasts. PDGFR (PDGFRa or PDGFRb)‐specific deletion in CD34^+^ cells in vivo revealed that CD34^+^ cells give rise to Periostin^+^ myofibroblasts through PDGFRb and not PDGFRa, whereas both receptors are required for CD34^+^ cell proliferation in AAA. In vitro mechanistic studies demonstrated that PDGFRb interacts with PI3K to mediate Periostin expression and that the PDGFRb‐PI3K axis is indispensable for PDGFBB‐induced Periostin^+^ myofibroblast generation from CD34^+^ cells. Notably, Ang II appeared to play a minor role in Periostin expression in CD34^+^ cells. Therefore, we conclude that the activation and transdifferentiation of CD34^+^ cells into myofibroblasts in AAA is likely controlled by PDGFRb signaling induced by PDGF ligands secreted by other aortic cells, including ECs, SMCs, and macrophages, rather than by the direct effect of Ang II on CD34^+^ cells.

In terms of limitations, the specific function of CD34^+^ progenitors in patients with AAA requires further validation, despite our demonstration of their significant reduction in both human TAA and AAA. The translational application of CD34^+^ cell‐targeted therapies should emphasize balancing beneficial fibrotic reinforcement with the risk of adverse adventitial remodeling. Moreover, although the Ang II‐induced AAA model is ideal for studying fibrotic collars generated from adventitial myofibroblasts, the extraluminal application of CaCl_2_ may not perfectly mimic the pathophysiological conditions of aortic dilatation. CaCl₂ incubation causes chemical damage to the aortic adventitia, and rupture of infrarenal abdominal aortic aneurysms caused by CaCl_2_ is infrequent. The contribution of fibrous collars formed by myofibroblasts to the maintenance of AAA wall strength or the enhancement of aortic stability deserves further investigation.

## 4 Conclusion

In this study, we investigated the contribution of CD34^+^ cells to AAA development and progression using advanced techniques, including scRNA‐seq, dual recombinase‐mediated genetic lineage tracing, IF staining with 3D aortic reconstruction, and various gene‐modified mouse strains. These findings demonstrate that CD34^+^ cells are non‐BM‐derived fibroblast progenitors that transdifferentiate into myofibroblasts in AAA, forming a fibrous collar in the adventitial layer of the aneurysmal aorta to maintain the compromised structural integrity of the aneurysmal aortic wall. Furthermore, this study provides new regulatory insights into myofibroblast generation from CD34^+^ cells and demonstrates that the PDGFBB‐PDGFRb‐PI3K‐Akt axis is indispensable for this differentiation process. Therefore, this study suggests that targeting CD34^+^ cell‐derived myofibroblasts may be a promising therapeutic approach aimed at stabilizing aneurysms and reducing the rupture risk.

## 5 Experimental Section

A detailed description of the materials and methods used in this study is provided in the Supporting Information.

### Collection of Human Samples for Histological Analysis

Human tissue samples were collected in accordance with the protocol approved by the Research Ethics Committee at the First Affiliated Hospital of Zhejiang University School of Medicine. Local ethics approval and written informed patient consent for human aortic tissue collection were obtained before sample collection. (institutional review board approval No. 2021/330 and No. 2022/295). Non‐dilated ascending aortic samples were collected from heart transplant recipients and lung donors, non‐dilated abdominal aortic samples from the donors during kidney transplantation, and diseased thoracic aortic samples from sporadic thoracic aortic aneurysms as well as abdominal aortic aneurysms samples from patients underwent surgery. Aneurysmal aortic specimens were characterized by loss of SMCs and degradation of elastin identified by H&E and EVG staining. Clinical baseline characteristics of the study groups were summarized in supporting information (Table S1, Supporting Information), with detailed information provided in Supplementary Excel 1.

### Mice Generation and Breeding

Animal procedures were in accordance with the Guide for Care and Use of Laboratory Animals published by the US National Institute of Health (8th edition, 2011) and approval for the study was obtained from the Institutional Animal Care and Use Committee of Zhejiang University School of Medicine (No.2021/105).

The *Cd34*‐CreER^T2^ mice were generated by conventional embryonic stem cell gene targeting methods^[^ ^13^ ^]^ in Shanghai Model Organisms Center, Inc. *Postn*‐CreER^T2^ knock‐in mouse model, and *Cd34*‐Dre knock‐in mouse model were developed by Shanghai Model Organisms Center, Inc. Specifically, the CreER^T2^ targeting construct was designed to insert a P2A peptide and a CreER^T2^ fusion gene into the stop codon site of *Postn* gene. This construct was inserted into the targeted gene via CRISPR/Cas9 system in a C57BL/6J mouse background. The donor vector with Cas9 targeted guide RNA (sgRNA) and Cas9 mRNA was transcribed and purified in vitro, then microinjected into C57BL/6J fertilized eggs. F0 generation mice positive for homologous recombination were identified by long PCR spanning 5′ or 3′ homologous arm. The PCR products were further confirmed by sequencing. *Postn*‐CreER^T2^ heterozygous mice were obtained by crossing F0 mice with C57BL/6J mice. *Cd34* targeting vector, Kozak‐Dre‐wpre‐PA, was designed and constructed that be inserted into between the 5′ UTR and first exon of *Cd34* targeted allele through homologous recombination. Then F0 mice were bred with C57BL/6J mice to obtain *CD34*‐Dre heterozygous mice. The Rosa26‐tdTomato (JAX: 007909), Rosa26‐DTR (JAX: 007900), Dou‐tdT‐DTR (Rosa26‐CAG‐LSL‐RSR‐tdTomato‐2A‐DTR, Cat.NO.NM‐KI‐190086) and *Apoe* ^−/−^ (Jax: 002052) mice lines were purchased from Shanghai Model Organisms Center. *Cd34*‐CreER^T2^;R26‐tdTomato;*Apoe^−/−^* mice were obtained by crossing *Cd34*‐CreER^T2^ with Rosa26‐tdTomato mice and *Apoe* ^−/−^ mice. *Cd34*‐CreER^T2^;R26‐DTR/tdTomato;*Apoe* ^−/−^ mice were obtained by crossing *Cd34*‐CreER^T2^ mice with Rosa26‐tdTomato mice, Rosa26‐DTR mice and *Apoe* ^−/−^ mice. *Postn*‐CreER^T2^;R26‐ tdTomato mice were obtained by crossing *Postn*‐CreER^T2^ mice and Rosa26‐tdTomato mice. *Cd34*‐Dre;*Postn*‐Cre ER^T2^;Dou‐tdT‐DTR mice were obtained by crossing *Postn*‐CreER^T2^ mice, *Cd34*‐Dre mice, and Dou‐tdT‐DTR mice.

CD34^+^ cells‐specific conditional knockout *Pdgfra* or *Pdgfrb* genes were generated as follows. *Pdgfra^flox/flox^* (T007274) mice line were purchased from GemPharmatech CO., Ltd, and *Pdgfrb^flox/flox^* mice line was generated by the Shanghai Model Organisms Center. In brief, to obtain Pdgfra alleles appropriate for Cre‐mediated conditional inactivation of *Pdgfra*, a targeting vector was designed and constructed in which two loxP sites flanking exons 3 and 5 of the *PDGFRa* gene could be inserted through homologous recombination. Then *Pdgfra* ^flox/flox^ mice were bred with *Cd34*‐CreER^T2^;R26‐tdT;*Apoe^−/−^* transgenic mice to generate the *Cd34*‐CreER^T2^;R26‐tdT*;Pdgfra^flox/flox^*;*Apoe^−/−^*. Similarly, a targeting vector was designed and constructed that could insert two loxP sites through homologous recombination flanking exons 4 and 7 of the *Pdgfrb* gene in mouse embryonic stem cells. Then *Pdgfrb^flox/flox^* mice were bred with *Cd34*‐CreER^T2^;R26‐tdT;*Apoe^−/−^* transgenic mice to generate the *Cd34*‐CreER^T2^;R26‐tdT*;Pdgfrb^flox/flox^*;*Apoe^−/−^*.

### rAAV8‐D377Y‐mPCSK9 Overexpression in *Cd34*‐Dre;*Postn*‐Cre ER^T2^;Dou‐tdT‐DTR Mice

The adeno‐associated virus serotype 8 (AAV8) vector encoding gain‐of‐function mutant mouse PCSK9 (proprotein convertase subtilisin/kexin type 9) (rAAV8‐D377Y‐mPCSK9) and empty vector were constructed by OBiO Technology^[^ ^25^ ^]^ (Shanghai, China). The virus (1 × 10^12^ vg mL^−1^) were administered to 8‐week‐old *Cd34*‐Dre;*Postn*‐Cre ER^T2^;Dou‐tdT‐DTR mice (0.2 mL per animal) through tail vein injection, and animals were challenged with high‐fat diet containing 40 kcal% fat, 1.25% cholesterol and 0.5% sodium cholate (D12109C, Research Diets) for 6 weeks. Then Ang II‐induced abdominal aortic aneurysm (AAA) model was established. The primer sequences used for plasmid construction are in Table S2, (Supporting Information).

### Abdominal Aortic Aneurysm Animal Models

All mice were raised at 24 ± 2 °C and 40 ± 5% humidity under a 12 h light/dark cycle with access to diet and water ad libitum. The Ang II‐induced AAA model was implemented as previously described. Briefly, a mini osmotic pump (Alzet model 1004, 28‐day delivery; Durect Corporation, USA) loaded with Ang II (1000 ng kg^−1^ min^−1^, A9525; Sigma, MO, USA) or saline was subcutaneously infused into 12 to 14‐week‐old male mice for 4 weeks. Then mice were anesthetized, and the whole aortas were harvested at 28 days after implantation surgery. The aorta was considered to be aneurysmal if the abdominal aorta diameter or maximal outer width of the suprarenal aorta increased by 50% or more. Any mouse that died prior to the study endpoint was subjected to autopsy, and aneurysm rupture was characterized by the presence of egress with blood clots outside the adventitia of the dilated aortic wall.

The calcium chloride (CaCl_2_) adventitial application model was carried out as previously described.^[^ ^39^ ^]^ 12‐week‐old male and female mice were both used for the CaCl_2_‐induced aneurysm model and were randomly allocated to different experimental groups. CaCl2‐incubated infrarenal abdominal aortic aneurysms were characterized by VSMC apoptosis at the incubation site, with relatively smaller changes in aorta diameter. Briefly, the infrarenal region of the abdominal aorta was separated through a midline incision under general anesthesia. 0.5mol L^−1^ CaCl_2_ soaked filter paper of appropriate size was applied peri‐vascularly for 10 min. Replace this filter paper with another PBS‐impregnated filter paper for 5 min. Two weeks after modeling, the mice were euthanized and tissues were perfusion‐fixed with a mixture of 4% paraformaldehyde at physiologic perfusion pressure. The harvested abdominal aorta was further fixed in 4% paraformaldehyde and a follow‐up histological analysis was performed.

Based on the NIH guidelines for animal euthanasia, mice were euthanized with carbon dioxide (CO_2_) four weeks after having the Ang II osmotic pump implanted, with minimal stress to them. Briefly, a cage containing 3–5 mice was placed in a separate 20‐litre volume chamber. Compressed 99.99% CO_2_ gas in a cylinder was connected to and introduced into the chamber with a flow rate of 10 liters per minute. The mice were all unconscious in 3 min, lacking spontaneous breathing. After another minute's CO_2_ flow, the mice were checked again to confirm with no respiration, their eye color faded, and no pupillary response to light. The mice were then removed from the cage, the chests were cut open and the hearts were perfused through left ventricular puncture with PBS. Aortas were then harvested for single‐cell RNA sequencing, flow cytometric analyses, and immunostaining (another 15 min 4% PFA perfusion after PBS perfusion), and the mice were then confirmed dead by removing the hearts.

### Genetic Labeling or Depleting CD34^+^ Cells

Labeling or depleting CD34^+^ cells was described previously.^[^ ^13^ ^]^ Briefly, to label CD34^+^ cells in *Cd34*‐CreER^T2^;R26‐tdTomato mice, Tamoxifen (Sigma, T5648, 0.15mg g^−1^ body weight) was administered by gavage every 3 days with a total of 4 pulses. To delete CD34^+^ cells, diphtheria toxin (20 ng g^−1^ body weight by 4 intraperitoneal injections every 2 days with a total of 5 pulses) in *Cd34*‐CreER^T2^;R26‐DTR/tdTomato mice before AAA animal model was performed. Diphtheria toxin (Sigma, D0564) was dissolved in sterile PBS to a storage dilution of 2 µg mL^−1^.

### Ultrasound Image Acquisition

Ang II‐induced AAA progression was longitudinally and transversely monitored at different time points in vivo using high‐frequency ultrasounds. Ultrasound imaging was performed with mice placed supine on a heated table under isoflurane anesthesia and depilated with hair removal cream. High‐resolution ultrasound imaging system (VINNO, 650LAB) with 10–23 MHz frequency real‐time microvisualization scan‐head (X10‐23L) and 15 × 15 mm field of view was used first in B‐mode to obtain a 2D‐transverse image to localize the suprarenal abdominal aorta, while abdominal blood flow was visualized by Color Doppler and Pulsed Doppler measurements. Blood flow toward the transducer was shown in red, while away from the transducer in blue. The retrograde (cranially directed) flow was observed in the false channel. The ultrasound operator manually assessed the delineation of the vessel and remodeled the wall. Measurements were taken before treatment initiation to determine the baseline diameters and were repeated several times during the experiment.

### Immunostaining, Histological Analysis, and 3D Reconstruction of Aortas

In order to stain cryo‐sections of mouse aortas with immunofluorescence (IF), aortas were harvested, washed with PBS, and fixed with 4% paraformaldehyde for 37 h at 4 °C, then aortas were dehydrated in 30% sucrose solution at 4 °C overnight until fully penetrated. Tissues were embedded in optimum cutting temperature (O.C.T., Sakura, 4583), frozen at −80 °C for storage, or cut into 5‐µm sections. The cryosections were air‐dried for ≈30 min at room temperature and then blocked for 1 h, followed by primary antibody staining overnight at 4 °C and then incubated with Alexa Fluor‐conjugated secondary antibodies (Invitrogen, 1:500) for 1 h. As counterstain, the DAPI counterstain reagent (Servicebio, G1012) was used to stain nuclei. Finally, the slides were mounted in the anti‐fade mounting medium (Servicebio, G1401). To eliminate the specificity of each signal, isotype IgG control for primary antibodies (Invitrogen, Cat No. 31933, 02–6102, 31903, 31245) for each host species were used as negative controls, together with secondary antibody‐only controls to validate the specificity of antibodies.

IF staining of cultured cells requires washing with PBS followed by 15 min of fixation in 4% PFA. After permeabilizing in PBS with 0.5% Triton X‐100 for 15 min, samples were blocked in 5% donkey serum for 1 h, followed by staining according to the same protocol as described above. A Leica TCSSP8 DIVE confocal microscope and a Zeiss LSM 900 Airyscan 2 were used to acquire cryo‐section and cell staining images, further analyzed by relevant software. Regions were selected randomly to avoid biasing. The Image Pro Plus software 6.0 software (Media Cybernetics, Inc.) was used for image analyses.

For tissue clearing and 3D reconstruction, tissue was harvested and fixed overnight at 4 °C, followed by 3 × 1 h's wash in PBS. The tissue was next immersed in CUBIC‐L solution [10% (wt/wt) N‐butyldiethanolamine (TCIchemicals, B0725) and 10% (wt/wt) Triton X‐100 in ddH_2_O] at 37 °C for 2 days. After another 3×1 h's wash in PBS, the tissue was immersed in the primary antibody solution and incubated at 4 °C for 2 days, and another 3×1 h's wash in PBS was followed. Tissue was then incubated with the secondary antibodies at room temperature for 1 day, and washed 3 times by PBS. After that, the tissue was immersed in CUBIC‐R^+^ solution [45% (wt/wt) antipyrine (TCIchemicals, D1876), 30% (wt/wt) nicotinamide (TCIchemicals, N0078), 0.5% (vol/vol) N‐butyldiethanolamine in ddH2O] for transparency. Images were obtained using a Leica TCSSP8 DIVE confocal microscope and further reconstructed using the Imaris 9.0.1 (Bitplane, Switzerland) software.

The procedures for staining hematoxylin and eosin (H&E), Masson's trichrome, Picro‐Sirius red (PSR), Victoria Blue (VB), and EVG were carried out according to the instructions of the manufacturer. PSR and VB stain (PSR‐VB stain) were combined to specifically highlight elastic fibers and collagen in the same section. The Image Pro Plus 6.0 software (Media Cybernetics, Inc.) was used for image analyses. The investigators were blinded to different groups when performing histology staining and analyzing the data.

### Single‐Cell RNA Sequencing of Aortic Cells with 10× Chromium and Data Analysis

The abdominal aortas of male 16–18‐week‐old *Cd34*‐CreER^T2^;R26‐tdTomato; *Apoe* ^−/−^ mice were harvested from sham‐treated and Ang II‐inducted groups (8 mice per group). After full digestion, the cell pellet was suspended in PBS and stained with CD45‐FITC (BD Pharmingen, 553079, 1:100) for 30 min, and then stained with LIVE/DEAD Fixable Near‐IR Dead Cell Stain Kit (1:1000) and Hoechst 33342 (Invitrogen, H3570, 1:1000) for 20 min on ice. After PBS washing, cells were resuspended in PBS and single nucleated live tdTomato^+^ or tdTomato^+^CD45^−^ cells (Hoechst^+^ & Dead Cell Stain & tdTomato^+^) were sorted into PBS with 0.04% BSA using a BD FACS ARIA II Flow Cytometer (BD Biosciences). A ChromiumTM Single Cell Reagent Kit v3 Chemistry (10x Genomics) was used along with a standard protocol. The library was generated and sequenced on a Novaseq6000 PE150 platform (Illumina) with a paired‐end 150 bp sequencing strategy. The 10x ChromiumTM procedure, library generation, and sequencing were performed by Novogene Co., Ltd (Beijing, China). Moreover, the raw data of whole aortic cell datasets (Sham 4 weeks and Ang II 4 weeks) reported in our previous article were available in Gene Expression Omnibus (GSE221789). The scRNA‐seq datasets for human aortic aneurysms (GSE155468 and GSE166676) were collected from public repositories.

Single‐cell RNA‐sequencing raw data were processed using Cell Ranger (version 6.0). Aligned reads and gene‐barcode matrices were then generated from FASTQ files including Read 1, Read 2, and i7 index. Cellranger mkfastq demultiplexed raw data and produced FASTQ files, which were further processed by “Cellranger count” to align reads to the mouse reference to count the number of barcodes and UMI and to generate feature‐barcode matrices. At a sequencing depth of 30 mb per sample, 3008 genes were detected on average per cell.

The Seurat package (version 4.0.1) was used to perform cell filtration, data normalization, dataset integration, dimension reduction, cell clustering, and cluster visualization using the default parameters unless otherwise specified.^[^ ^40^ ^]^ Briefly, cells expressing <400 or >7500 genes were filtered out to exclude noncell or cell aggregates, and cells with >5% mitochondrial gene percentage were also filtered out to exclude cells at a compromised state. Then doublet cells were filtered with the DoubletFinder R package.^[^ ^41^ ^]^ After alignment and quality control, a total of 34 329 cells (Sham 4 weeks:5024 cells; Ang II 4 weeks: 12127 cells; Sham tdT: 6541 cells; Ang II tdT 10637 cells) were aggregation and included in the subsequent analysis. “ScaleData” was used to scale the top 2000 highly variable genes after log‐normalization. Principle component analysis was then performed on selected highly variable genes, and the first 30 principal components with a resolution of 0.5 were used for cell clustering and uniform manifold approximation and projection (UMAP) visualization.

### Mouse Vascular Adventitial CD34^+^ Cell Isolation, Cell Culture and Differentiation

Mouse aortic stem/progenitor cells were isolated as previously described.^[^ ^42^ ^]^ Briefly, the entire aorta was harvested and cut open. The adventitial layers were carefully dissected from the medial and intimal layers. The isolated aortic adventitia was then sliced into small pieces, seeded in a 0.04% gelatin (Sigma, G1393)‐coated T25 flask and maintained in complete cell culture medium, which consists of DMEM (Sigma, D6429), 10% Fetal Bovine Serum FBS (HyClone, SH30396.03), 100 U mL^−1^ penicillin‐streptomycin (Gibco, 15140122), 2% chick embryo extract (MP Biomedical), 100 nm retinoic acid (Sigma–Aldrich), 50 nm 2‐mercaptoethanol (Sigma–Aldrich), 2% B27 (Invitrogen), 1% N2 (Invitrogen) and 20 ng mL^−1^ bFGF (R&D Systems). Cells derived from the outgrowth of aortic adventitia were then passaged for cell enrichment. Primary aortic cells were maintained in a complete cell culture medium and passaged at a ratio of 1:3 every 3 days. The cell culture medium was changed every other day. When expanded to the fifth generation, CD34^+^ cells were sorted into DMEM with 20% FBS using a BD FACS ARIA II Flow Cytometer (BD Biosciences). The purified CD34^+^ cells were expanded and cultured for subsequent experiments.

DMEM containing 10% FBS and 1% P/S was used to maintain CD34^+^ cells in differentiation experiments. CD34^+^ cells were serum‐starved by culturing for 24 h in DMEM without serum and then treated with 200ng mL^−1^ PDGFBB (MCE, HY‐P7087) for 12 h to induce CD34^+^ cell activation. Moreover, CD34^+^ cells were also infected with Ad‐*Pdgfrb* (10^8^ pfu mL^−1^) for 48 h and treated with 12 h 15 µm LY294002 (inhibitor of PI3K) combined with 200ng mL^−1^ PDGFBB (MCE, HY‐P7087) prior to any further analysis.

### Western Blot

Human aortic samples and mice abdominal aortic samples were collected and homogenized in RIPA lysis buffer containing protease inhibitor cocktail (78425, Thermo Scientific, 1:100); Protein concentration was determined by BCA protein assay kit (23225, Thermo Scientific). Protein extracts were boiled at 95 °C for 10 min in 1X loading buffer, then subjected to SDS‐PAGE and transferred to PVDF membranes. Followed by blocking for 1 h at room temperature in TBST with 5% no‐fat milk, the membranes were incubated with primary antibodies at 4 °C overnight. Following three washes in TBST buffer, the membranes were incubated with goat‐anti rabbit IgG (Beyotime) conjugated with horseradish peroxidase (HRP), goat‐anti mouse IgG (Beyotime) secondary antibodies for 1 h at room temperature. The membranes were then washed three times in TBST buffer, and chemiluminescent signals were detected with Pierce ECL western blotting substrates and analyzed by Image Lab (v6.0). The primary antibodies used are listed in the supporting information.

### Statistical Analysis

GraphPad Prism 9.0 was used for statistical analysis and image creation. Numbers refer to independent experiments or mice as indicated in figure legends. Data are presented as mean ± SD. For human and animal data, normality was tested by the D'Agostino–Pearson (*n* > 8) or Shapiro–Wilk (*n =* 3 to 8) test. Normal distribution data between two groups were tested by unpaired two‐tailed t‐test, with Welch correction for unequal variance; nonparametric Mann‐Whitney tests were used for abnormal distributions. For >2 groups, normal distribution data were tested by ordinary 1‐way analysis of variance with the Tukey test (equal SDs) or by Brown‐Forsythe and Welch analysis of variance tests with Tamhane's T2 test (unequal SDs). Survival curves were analyzed by log‐rank (Mantel‐Cox) test. For cell culture data, 4 groups of data were first tested by the Shapiro–Wilk test and then assessed by ordinary one‐way ANOVA analysis (Tukey's multiple comparisons test). 6 groups of data were tested by two‐way ANOVA test. *p <*0.05 was considered to be statistically significant.

### Data Availability Statement

Data and R scripts related to the findings of this study are available upon request. The scRNA‐seq data of this study are available in the Gene Expression Omnibus (GSE260887). A previously published mouse scRNA‐seq dataset (GSE221789)^[^ ^10^ ^]^ from our group, the normal mouse and CaCl_2_‐induced aneurysmal aorta datasets (GSE164678),^[^ ^18^ ^]^ and two scRNA‐seq datasets for human aortic aneurysms^[^ ^17^ ^]^ (GSE155468 and GSE166676) are available in public repositories.

## Conflict of Interest

The authors declare no conflict of interest.

## Author Contributions

H.W., X.Y., and T.C. contributed equally to this work. Q.X., H.Y., Q.X., H.Z., J.L., and H.W. conceptualized and designed the research. H.W., X.Y., T.C., M.C., T.W., L.J., X.Z., K.C., Y.H., S.X., and B.Z. performed most bench work and data analyses. H.W. wrote the original draft. T.C., B.Y., L.J., J.C., T.Z., Q.X., and Q.X. reviewed and edited the manuscript. All authors read and approved the manuscript.

## Supporting information



Supporting Information

Supporting Table 1

Supporting Table 2

## Data Availability

The data that support the findings of this study are available in the supplementary material of this article.;
