# Supplementary material for: Aneurysm Is Restricted by CD34+ Cell‐Formed Fibrous Collars Through the PDGFRb‐PI3K Axis
Source: Adv Sci (Weinh). 2024 Dec 27;12(7):2408996. doi: 10.1002/advs.202408996 (PMC11831520; doi:10.1002/advs.202408996)
Supplement: Supplementary file 1 — Supporting Information [file ADVS-12-2408996-s003.pdf]

ADVANCED SCIENCE
Open Access

Supporting Information

for *Adv. Sci.*, DOI 10.1002/advs.202408996

Aneurysm Is Restricted by CD34$^+$ Cell-Formed Fibrous Collars Through the PDGFRb-PI3K Axis

Hong Wu, Xiaoping Yang, Ting Chen, Baoqi Yu, Mengjia Chen, Ting Wang, Liujun Jiang, Bohuan Zhang, Xuhao Zhou, Junning Cheng, Kai Chen, Tao Zhang, Yanhua Hu, Simon Xu, Jiangfang Lian, Hongkun Zhang, Qingzhong Xiao, Honghua Ye* and Qingbo Xu**

Supporting Information

Title: Aneurysm Is Restricted by CD34^{+} Cell-Formed Fibrous Collars through the PDGFRb-PI3K Axis

Hong Wu[1], Xiaoping Yang[2], Ting Chen[1], Baoqi Yu[3], Mengjia Chen[1], Ting Wang[1], Liujun Jiang[1], Bohuan Zhang[1], Xuhao Zhou[1], Junning Cheng[1], Kai Chen[1], Tao Zhang[4], Yanhua Hu[1], Simon Xu[5], Jiangfang Lian[2], Hongkun Zhang[6], Qingzhong Xiao[7], Honghua Ye[2]* and Qingbo Xu[1]**

* **Correspondence author**

H. Wu, T. Chen, M. Chen, T. Wang, L. Jiang, B. Zhang, X. Zhou, J. Cheng, K. Chen, Y. Hu, Q. Xu
Department of Cardiology
The First Affiliated Hospital
Zhejiang University School of Medicine
Hangzhou, 310003, China
E-mail: qingbo_xu@zju.edu.cn

X. Yang, J. Lian, H. Ye
Department of Cardiology
Ningbo Institute of Innovation for Combined Medicine and Engineering,
Lihuili Hospital Affiliated to Ningbo University
Ningbo University
Ningbo, Zhejiang, China
E-mail: lindayenbzjch@163.com

B. Yu
Department of Physiology and Pathophysiology
School of Basic Medical Sciences
Capital Medical University; Key Laboratory of Remodeling-Related Cardiovascular Diseases, Ministry of Education;
Beijing Key Laboratory of Metabolic Disorder-Related Cardiovascular Diseases, Beijing, China

T. Zhang
Department of Vascular Surgery
Peking University People's Hospital
Beijing, China

S. Xu
Department of Surgery
Liverpool Heart and Chest Hospital
Liverpool, United Kingdom

H. Zhang
Department of Vascular Surgery

The First Affiliated Hospital
Zhejiang University School of Medicine
Hangzhou, China

Q. Xiao
Centre for Clinical Pharmacology and Precision Medicine
William Harvey Research Institute
Faculty of Medicine and Dentistry
Queen Mary University of London
London, United Kingdom
E-mail: q.xiao@qmul.ac.uk

Keywords: aneurysm, $CD34^+$ cell, fibroblast, PDGFRb, genetic cell lineage tracing

SUPPLEMENTAL MATERIAL

Materials and Methods

Collection of Human Samples for Histological Analysis

Human tissue samples were collected in accordance with the protocol approved by the Research Ethics Committee at the First Affiliated Hospital of Zhejiang University School of Medicine. We collected non-dilated ascending aortic samples from heart transplant recipients and lung donors, non-dilated abdominal aortic samples from the donors during kidney transplantation, and diseased thoracic aortic samples from sporadic thoracic aortic aneurysms as well as abdominal aortic aneurysms samples from patients underwent surgery. Aneurysmal aortic specimens were characterized by loss of SMCs and degradation of elastin identified by H&E and EVG staining. Clinical baseline characteristics of the study groups were summarized in **Table S1 and detailed information in Supplementary Excel 1**. Exclusion criteria included: major organ diseases such as liver failure, dialysis, cancer, chemotherapy, or pregnancy, or lack of consent to participate in the study. All studies were performed with approval from the Research Ethics Committees of the First Affiliated Hospital of Zhejiang University School of Medicine (institutional review board approval No. 2021/330 and No. 2022/295). All patients gave their written, informed consent to sample collection. All experiments were conducted according to the principles expressed in the Declaration of Helsinki. For histopathological analysis (n=12 individuals/non-dilated TA group, n=21 individuals/TAA group, n=6 individuals/ non-dilated AA group, n= 16 individuals/AAA group), human aortic specimens were fixed with 4% paraformaldehyde (PFA, Servicebio, G1101) and proceeded to further paraffin-embedded sections; for western blot, human samples were randomly selected from the indicated group (n=4 patients).

Immunohistochemistry Staining

Human aortic samples were embedded in paraffin and cut into 5-μm-thick sections using a Leica RM2235 manual rotary microtome. Tissue sections were deparaffinized and hydrated in gradient xylene and ethanol. Sections were then incubated in 3% H_2O_2 for 10 mins to quench endogenous peroxidase activity. Antigen retrieval was performed by 98°C water bath for 25 mins. We prepared an antigen retrieval solution of 10mM Tris, 1mM EDTA, and 0.05% Tween-20 (pH 9.0). After cooling tissue down to room temperature (RT), tissue was blocked and permeabilized with 5% BSA + 0.1% Triton X-100 (Sigma, T8787) for 1 hour at RT, 5% BSA + 0.1% PFA was used to prevent cross-reactivity, and stained with primary antibodies (CD34, Abcam, ab8158, 1:100) overnight, then incubated with HRP-conjugated secondary antibodies (Beyotime Biotechnology, A0192, 1:50) for 1 h at 37°C. Sections were then incubated in horseradish peroxidase substrate solution (3,3N-Diaminobenzidine Tertrahydrochloride, DAB Horseradish Peroxidase Color Development Kit, ZSGB-bio, ZLI-9017, 1:20). The time for color development was carefully observed under a microscope. After coloration, sections were counterstained with hematoxylin for 5 mins, then dehydrated through increasing concentrations of ethanol and xylene, and finally mounted with neutral resin. Rat IgG isotype control (Invitrogen, 31933) was used as negative control and a secondary antibody only control was also used in immunofluorescence staining. A PANNORAMIC 250 Flash slide scanner (3DHISTECH) was used to acquire images. The Image pro plus 6.0 software (Media Cybernetics, Inc.) was used for image analyses. CD34-positive area (stained as brown) and integrate optical density (IOD)/area were pointed and calculated. The average optical density (IOD/CD34-positive area) were calculated as the data point. The investigators were blinded to different groups when performing immunostaining and analyzing the data.

Mice Generation and Breeding

Animal procedures were in accordance with the Guide for Care and Use of Laboratory Animals published by US National Institute of Health (8th edition, 2011) and approval for the study was obtained from the Institutional Animal Care and Use Committee of Zhejiang University School of Medicine (No.2021/105).

The *Cd34*-CreER^{T2} mice was generated by conventional embryonic stem cell gene targeting methods[1] in Shanghai Model Organisms Center, Inc. *Postn*-CreER^{T2} knock-in mouse model and *Cd34*-Dre knock-in mouse model were developed by Shanghai Model Organisms Center, Inc. Specifically, the CreER^{T2} targeting construct was designed to insert a P2A peptide and a CreER^{T2} fusion gene into the stop codon site of *Postn* gene. This construct was inserted into the targeted gene via CRISPR/Cas9 system in C57BL/6J mouse background. The donor vector with Cas9 targeted guide RNA (sgRNA) and Cas9 mRNA was transcribed and purified in vitro, then microinjected into C57BL/6J fertilized eggs. F0 generation mice positive for homologous recombination were identified by long PCR spanning 5' or 3' homolgous arm. The PCR products were further confirmed by sequencing. *Postn*-CreER^{T2} heterozygous mice were obtained by crossing F0 mice with C57BL/6J mice. *Cd34* targeting vector, Kozak-Dre-wpre-PA, was designed and constructed that could insert into between the 5' UTR and first exon of *Cd34* targeted allele through homologous recombination. Then F0 mice were bred with C57BL/6J mice to obtain *CD34*-Dre heterozygous mice. The Rosa26-tdTomato (JAX: 007909), Rosa26-DTR (JAX: 007900), Dou-tdT-DTR (Rosa26-CAG-LSL-RSR-tdTomato-2A-DTR, Cat.NO.NM-KI-190086) and *Apoe*$^{-/-}$ (Jax: 002052) mice lines were purchased from Shanghai Model Organisms Center. *Cd34*-CreER^{T2};R26-tdTomato;*Apoe*$^{-/-}$ mice were obtained by crossing *Cd34*-CreER^{T2} with Rosa26-tdTomato mice and *Apoe*$^{-/-}$ mice. *Cd34*-CreER^{T2};R26-DTR/tdTomato;*Apoe*$^{-/-}$ mice were obtained by crossing *Cd34*-CreER^{T2} mice with Rosa26-tdTomato mice, Rosa26-DTR mice and *Apoe*$^{-/-}$ mice. *Postn*-CreER^{T2};R26- tdTomato mice were obtained by crossing *Postn*-CreER^{T2} mice and Rosa26-tdTomato mice. *Cd34*-Dre;*Postn*-Cre ER^{T2};Dou-tdT-DTR mice were obtained by crossing *Postn*-CreER^{T2} mice, *Cd34*-Dre mice and Dou-tdT-DTR mice.

CD34^{+} cells-specific conditional knockout *Pdgfra* or *Pdgfrb* genes were generated as followed. *Pdgfra*$^{flox/flox}$ (T007274) mice line were purchased from GemPharmatech CO., Ltd and *Pdgfrb*$^{flox/flox}$ mice line was generated by Shanghai Model Organisms Center. In brief, to obtain Pdgfra alleles appropriate for Cre-mediated conditional inactivation of *Pdgfra*, we designed and constructed a targeting vector in which two loxP sites flanking exons 3 and 5 of the *PDGFRa* gene could be inserted through homologous recombination. Then *Pdgfra*$^{flox/flox}$ mice were bred with *Cd34*-CreER^{T2};R26-tdT;*Apoe*$^{-/-}$ transgenic mice to generate the *Cd34*-CreER^{T2};R26-tdT;*Pdgfra*$^{flox/flox}$;*Apoe*$^{-/-}$. Similarly, a targeting vector was designed and constructed that could insert two loxP sites through homologous recombination flanking exons 4 and 7 of the *Pdgfrb* gene in mouse embryonic stem cells. Then *Pdgfrb*$^{flox/flox}$ mice were bred with *Cd34*-CreER^{T2};R26-tdT;*Apoe*$^{-/-}$ transgenic mice to generate the *Cd34*-CreER^{T2};R26-tdT;*Pdgfrb*$^{flox/flox}$;*Apoe*$^{-/-}$.

Genomic DNA from mice tail tissue was prepared by proteinase K lysed, isopropanol precipitated and 70% ethanol washed and then prepared for genotyping.

rAAV8-D377Y-mPCSK9 Overexpression in *Cd34*-Dre;*Postn*-Cre ER^{T2};Dou-tdT-DTR Mice

The adeno-associated virus serotype 8 (AAV8) vector encoding gain-of-function mutant mouse PCSK9 (proprotein convertase subtilisin/kexin type 9) (rAAV8-D377Y-mPCSK9) and empty vector were constructed by OBiO Technology[2] (Shanghai, China). The virus (1×10^{12} vg/ml) were administered to 8-week-old *Cd34*-Dre;*Postn*-Cre ER^{T2};Dou-tdT-DTR mice (0.2 ml/animal) through tail vein injection, and animals were challenged with high-fat diet containing 40 kcal% fat, 1.25% cholesterol and 0.5% sodium cholate (D12109C, Research

Diets) for 6 weeks. Then Ang II-induced abdominal aortic aneurysm (AAA) model was established. The primer sequences used for plasmids construction are listed in **Table S2**.

Abdominal Aortic Aneurysm Animal Models

All mice were raised at 24 ± 2°C and 40 ± 5% humidity under a 12-h light/dark cycle with access to diet and water *ad libitum*. The Ang II-induced AAA model was implemented as previously described. Briefly, a mini osmotic pump (Alzet model 1004, 28-day delivery; Durect Corporation, USA) loaded with Ang II (1000 ng/kg/min, A9525; Sigma, MO, USA) or saline was subcutaneously infused into 12 to 14-week-old male mice for 4 weeks. Then mice were anesthetized, and the whole aortas were harvested at 28 days after implantation surgery. The aorta was considered to be aneurysmal if the abdominal aorta diameter or maximal outer width of suprarenal aorta increased by 50% or more. Any mouse that died prior to the study endpoint was subjected to autopsy, and aneurysm rupture was characterized by the presence of egress with blood clots outside the adventitia of the dilated aortic wall.

The calcium chloride ($CaCl_2$) adventitial application model was carried out as previous described.[3] 12-week-old male and female mice were both used for $CaCl_2$-induced aneurysm model and were randomly allocated to different experimental groups. CaCl2-incubated infrarenal abdominal aortic aneurysms were characterized by VSMC apoptosis at the incubation site, with relatively smaller changes in aorta diameter. Briefly, the infrarenal region of the abdominal aorta was separated through a midline incision under general anesthesia. 0.5mol/L $CaCl_2$ soaked filter paper of appropriate size was applied peri-vascularly for 10 minutes. Replace this filter paper with another PBS impregnated filter paper for 5 minutes. Two weeks after modeling, the mice were euthanized and tissues were perfusion-fixed with a mixture of 4% paraformaldehyde at physiologic perfusion pressure. Harvested abdominal aorta was further fixed in 4% paraformaldehyde and follow-up histological analysis was performed.

Based on the NIH guidelines for animal euthanasia, mice were euthanized with carbon dioxide (CO_2) four weeks after having the Ang II osmotic pump implanted, with minimal stress to them. Briefly, a cage containing 3-5 mice was placed in a separate 20-litre volume chamber. Compressed 99.99% CO_2 gas in a cylinder was connected to and introduced into the chamber with a flow rate of 10 litres per minute. The mice were all unconsciousness in 3 minutes, lacking spontaneous breathing. After another minute's CO_2 flow, the mice were checked again to confirm with no respiration, their eye color faded and no pupillary response to light. The mice were then removed from the cage, the chests were cut open and the hearts were perfused through left ventricular puncture with PBS. Aortas were then harvested for single-cell RNA sequencing, flow cytometric analyses and immunostaining (another 15-minute 4% PFA perfusion after PBS perfusion), and the mice were then confirmed dead by removing the hearts.

Genetic Labeling or Depleting $CD34^+$ Cells.

Labeling or depleting $CD34^+$ cells was described previously.[1] Briefly, to label $CD34^+$ cells in *Cd34*-CreER^{T2};R26-tdTomato mice, Tamoxifen (Sigma, T5648, 0.15mg/g body weight) was administered by gavage every 3 days with a total of 4 pulses. To delete $CD34^+$ cells, diphtheria toxin (20 ng/g body weight by 4 intraperitoneal injection every 2 days with a total of 5 pulses) in *Cd34*-CreER^{T2};R26-DTR/tdTomato mice before AAA animal model was performed. Diphtheria toxin (Sigma, D0564) was dissolved in sterile PBS to a storage dilution of 2 μg/ml.

Ultrasound Image Acquisition

Ang II-induced AAA progression was longitudinally and transversely monitored at different time points in vivo using a high-frequency ultrasounds. Ultrasound imaging was performed with mice placed supine on a heated table under isoflurane anesthesia and depilated with hair removal cream. High-resolution ultrasound imaging system (VINNO, 650LAB) with 10-23 MHz frequency real-time microvisualization scan-head (X10-23L) and 15×15 mm field

of view was used first in B-mode to obtain a 2D-transverse image to localize the suprarenal abdominal aorta, while abdominal blood flow was visualized by Color Doppler and Pulsed Doppler measurements. Blood flow towards the transducer was shown in red, while away from the transducer in blue. Retrograde (cranially directed) flow was observed in the false channel. The ultrasound operator manually assessed delineation of the vessel and remodeled wall. Measurements were taken before treatment initiation to determine the baseline diameters and were repeated several times during the experiment

Immunostaining, Histological Analysis, and 3D Reconstruction of aortas

In order to stain cryo-sections of mouse aortas with immunofluorescence (IF), aortas were harvested, washed with PBS, and fixed with 4% paraformaldehyde for 37 hours at 4 °C, then aortas were dehydration in 30% sucrose solution at 4°C overnight until fully penetrated. Tissues were embedded in optimum cutting temperature (O.C.T., Sakura, 4583), frozen at -80 ℃ for storage or cut into 5-μm sections. The cryosections were air-dried for about 30 minutes at room temperature and then blocked for 1 hour, followed by primary antibody staining overnight at 4 °C and then incubated with Alexa Fluor-conjugated secondary antibodies (Invitrogen, 1:500) for 1 h. As counterstain, the DAPI counterstain reagent (Servicebio, G1012) was used to stain nuclei. Finally, the slides were mounted in the anti-fade mounting medium (Servicebio, G1401). Primary antibodies were used as listed: tdTomato (Rockland, 600-401-379, 1:500), tdTomato (Sicgen, Ab8181-200, 1:50), CD34 (Abcam, ab81829, 1:100), CD31 (R&D, AF3628, 1:50), COL1A1 (Abcam, ab34710, 1:150), DDR2 (R&D, MAB25381, 1:200), SMA-FITC (Sigma, F3777, 1:300), SM-MHC (Abcam, ab53219, 1:200), SM22 alpha (Abcam, ab14106, 1:200), CD45 (R&D, AF114, 1:50), CD68 (Abcam, ab125212, 1:100), PDGFRa (R&D, AF1062, 1:50), PDGFRb (R&D, AF1042, 1:50), Perilipin A (Abcam, ab61682, 1:300), Periostin (R&D, AF2955, 1:200), Vimentin (Abcam, Ab8978, 1:200). Secondary antibodies were used as listed: donkey anti-rabbit IgG Alexa Fluor 555 (Invitrogen, A-31572, 1:500), donkey anti-goat IgG Alexa Fluor 555 (Invitrogen, A-32816, 1:500), donkey anti-rat IgG Alexa Fluor 488 (Invitrogen, A-21208, 1:500), donkey anti-mouse IgG Alexa Fluor 488 (Invitrogen, A-21202, 1:500), donkey anti-goat IgG Alexa Fluor 488 (Invitrogen, A-11055, 1:500), donkey anti-rabbit IgG Alexa Fluor 647(Invitrogen, A-32795, 1:500), donkey anti-goat IgG Alexa Fluor 647 (Invitrogen, A-21447, 1:500). To eliminate the specificity of each signal, isotype IgG control for primary antibodies (Invitrogen, Cat No. 31933, 02-6102, 31903, 31245) for each host species were used as negative controls, together with secondary antibody only controls to validate specificity of antibodies

IF staining of cultured cells requires washing with PBS followed by 15 minutes of fixation in 4% PFA. After permeabilizing in PBS with 0.5% Triton X-100 for 15 min, samples were blocked in 5% donkey serum for 1 hour, followed by staining according to the same protocol as described above. A Leica TCSSP8 DIVE confocal microscope and a Zeiss LSM 900 Airyscan 2 were used to acquire cryo-section and cell staining images, further analyzed by relevant software. Regions were selected randomly to avoid biasing. The Image Pro Plus software 6.0 software (Media Cybernetics, Inc.) was used for image analyses.

For tissue clearing and 3D reconstruction, tissue was harvested and fixed overnight at 4°C, followed by 3 x 1h's wash in PBS. The tissue was next immersed in CUBIC-L solution [10% (wt/wt) N-butyldiethanolamine (TCIchemicals, B0725) and 10% (wt/wt) Triton X-100 in ddH_2O] at 37°C for 2 days. After another 3 x 1h's wash in PBS, the tissue was immersed in the primary antibody solution and incubated at 4°C for 2 days, and another 3 x 1h's wash in PBS was followed. Tissue was then incubated with the secondary antibodies at room temperature for 1 day, and washed 3 times by PBS. After that the tissue was immersed in CUBIC-R^+ solution [45% (wt/wt) antipyrine (TCIchemicals, D1876), 30% (wt/wt) nicotinamide (TCIchemicals, N0078), 0.5% (vol/vol) N-butyldiethanolamine in ddH2O] for transparency. Images were

obtained using a Leica TCSSP8 DIVE confocal microscope and further reconstructed using the Imaris 9.0.1 (Bitplane, Switzerland) software.

The procedures for staining haematoxylin and eosin (H&E), Masson's trichrome, Picro-Sirius red (PSR), Victoria Blue (VB) and EVG were carried out according to the instructions of the manufacturer. PSR and VB stain (PSR-VB stain) were combined to specifically highlight elastic fibers and collagen on the same section. The Image Pro Plus 6.0 software (Media Cybernetics, Inc.) was used for image analyses. The investigators were blinded to different groups when performing histology staining and analyzing the data.

Bone Marrow Transplantation

Briefly, mice were irradiated whole-body (9.0 Gy) at a lethal dose. We then harvested the bone marrow cells from donor mice using 1.5mL RPMI 1640 medium (BI, 01-100-1A). To obtain single cell suspensions, bone marrow cells were passed through 40-μm cell strainers (Falcon, 352331). Six hours later, irradiated recipient mice were administered with 5×10^6 donor bone marrow cells via tail vein injection to form chimeric mice. Subsequently, we observed the mice's condition for 14 days. Surviving mice were then treated with five consecutive pulses of tamoxifen for 1 week to induce tdTomato (tdT) labeling of $CD34^+$ cells, followed by Angiotensin II (Ang II) or $CaCl_2$ modeling a week later.

EdU in vivo Incorporation Assay.

Mice were treated with EdU through i.p. injections at a dosage of 50 mg/kg body weight/d every 5 days with a total of 5 pulses during Ang II induction period. EdU detection was carried out before IF staining using Cell-LightTM Apollo 488 Stain Kit (RiboBio, C10310-3).

Isolation of Single Cells from Abdominal Aorta

Mice were euthanized and perfused with 10 ml PBS and abdominal aortas were harvested in a petri dish containing pre-cooling DMEM (ATCC, 302002) with 10% fetal bovine serum (FBS, Gibco, 10099141) after perivascular adipose tissues and connective tissues were carefully removed. After collecting normal or aneurysmal abdominal aortas, vessels were washed, cut into fine pieces and incubated with 1 mg/ml papain digesting solution [1 mg/ml papain (Sigma, P4762), 0.5 mg/ml 1,4-Dithioerythritol (Sigma, D8255), 0.156 mg/ml Taurine (Sigma, T8691), 0.25 mg/ml bovine serum albumin (Solarbio®, A8020) in PBS]. We digested the cells at 37°C with reciprocal shaking in DMEM with 10% FBS and collected detached cells. Remaining aortic tissues were then incubated with fresh digestion solutions every 10 minutes. To dissolve the tissues completely, detached cells were collected and fresh digestion solutions were added. A 40-mm cell strainer was used to filter cells, followed by a centrifugation at 500g speed for 8 minutes at 4°C after sufficient digestion. Subsequently, cell pellet was suspended in relevant solutions for further experiments.

Flow Cytometric Analyses

Single cells from the aorta, bone marrow and blood were suspended in PBS with 5% FBS. Cells were then analyzed for tdTomato expression. Then, conjugated antibodies were used to stain the cells (1 μg per 10^6 cells) for 30 min at 4 °C. Conjugated-antibodies used include CD34-FITC (BD Pharmingen™, 555821), CD34-Alexa Fluor® 647 (BD Pharmingen™, 560230), Sca1 (BD Pharmingen™, 565355), CD45-PerCP-Cy™5.5 (BD Pharmingen™, 550994), CD31-BB700 (BD Horizon™, 566490), and PDGFRa-APC (eBioscience, 17-1401). After staining, LIVE/DEADTM Fixable Near-IR Dead Cell Stain Kit was used to stain the cells after washed by PBS (Invitrogen, L34975, 1:1000) for 20 mins to exclude dead cells. Cells were washed with PBS and re-suspended in PBS containing 5% FBS for flow cytometric analyses. Analyses were performed on BD LSR Fortessa II flow cytometers (BD Biosciences). FlowJo v10 software (BD Biosciences, USA) was used to analyze the flow cytometric data.

Single-cell RNA Sequencing of Aortic Cells with 10× Chromium and Data Analysis

The abdominal aortas of male 16-18-week-old *Cd34*-CreER^{T2};R26-tdTomato; *Apoe*$^{-/-}$ mice were harvested from sham-treated and Ang II-inducted groups (8 mice per group). After full digestion, the cell pellet was suspended in PBS and stained with CD45-FITC (BD Pharmingen™, 553079, 1:100) for 30 mins, and then stained with LIVE/DEAD ™ Fixable Near-IR Dead Cell Stain Kit (1:1000) and Hoechst 33342 (Invitrogen, H3570, 1:1000) for 20 mins on ice. After PBS washing, cells were resuspended in PBS and single nucleated live tdTomato^{+} or tdTomato^{+}CD45^{-} cells (Hoechst^{+} & Dead Cell Stain & tdTomato^{+}) were sorted into PBS with 0.04% BSA using a BD FACS ARIA II Flow Cytometer (BD Biosciences). A ChromiumTM Single Cell Reagent Kit v3 Chemistry (10x Genomics) was used along with a standard protocol. The library was generated and sequenced on a Novaseq6000 PE150 platform (Illumina) with paired-end 150 bp sequencing strategy. The 10x ChromiumTM procedure, library generation and sequencing were performed by Novogene Co., Ltd (Beijing, China). Moreover, the raw data of whole aortic cell datasets (Sham 4wks and Ang II 4wks) reported in our previous article were available in Gene Expression Omnibus (GSE221789). The scRNA-seq datasets for human aortic aneurysms (GSE155468 and GSE166676) was collected from public repositories.

Single-cell RNA-sequencing raw data were processed using Cell Ranger (version 6.0). Aligned reads and gene-barcode matrices were then generated from FASTQ files including Read 1, Read 2 and i7 index. Cellranger mkfastq demultiplexed raw data and produced FASTQ files, which were further processed by “Cellranger count” to align reads to the mouse reference to count the number of barcode and UMI, and to generate feature-barcode matrices. At a sequencing depth of 30 mb per sample, 3,008 genes were detected on average per cell.

The Seurat package (version 4.0.1) was used to perform cell filtration, data normalization, dataset integration, dimension reduction, cell clustering, and cluster visualization using the default parameters unless otherwise specified.[4] Briefly, cells expressing <400 or >7,500 genes were filtered out to exclude noncell or cell aggregates and cells with >5% mitochondrial gene percentage were also filtered out to exclude cells at a compromised state. Then doublet cells were filtered with DoubletFinder R package.[5] After alignment and quality control, a total of 34,329 cells (Sham 4wks:5024 cells; Ang II 4wks: 12127 cells; Sham tdT: 6541 cells; Ang II tdT 10637 cells) were aggregation and included in the subsequent analysis. "ScaleData" was used to scale the top 2,000 highly variable genes after log-normalization. Principle component analysis was then performed on selected highly variable genes, and the first 30 principal components with a resolution of 0.5 were used for cell clustering and uniform manifold approximation and projection (UMAP) visualization.

Differential Analysis for Clusters and Groups

Differential expression analysis for each cell type between different groups was performed using the Wilcoxon-test as implemented in the ‘‘FindAllMarkers’’ function of the Seurat V4 package. For each cluster, the DEGs (differentially expressed genes) were analyzed using the ‘‘FindAllMarkers’’ function. DEGs (between two groups) were identified using the “FindMarkers”in Seurat according to the following criteria: (1) a log2 fold change >0.25, (2) Adjusted p-value <0.05. (3) >3% of cells in either test group. Adjusted p-value based on bonferroni correction using all features in the dataset.

Gene Enrichment Analyses

Gene ontology analyses of DEGs were performed using the R package clusterProfiler (https://github.com/YuLab-SMU/clusterProfiler),[6] which supports statistical analysis and visualization of functional profiles for genes and gene clusters. With the annotation database org.Mm.eg.db, biological processes (BP) of Gene Ontology (GO) were annotated with the

function EnrichGO . BP expression level means average expression of the gene sets in the BP annotation using the online database(geneontology.org).

Pseudotime Trajectory Analyses

R package monocle (version 2.14) with default settings unless otherwise specified was used to analyze pseudotime trajectory. Genes used for pseudotime ordering were taken from the first 250 (by avg $\log_2$ fold change and adjusted p-value) DEGs identified by function differentialGeneTest with fullModelFormulaStri set as pseudotime. Dimensions were reduced and cells were ordered along the pseudotime trajectory using the DDRTree method. Branch analysis was performed with BEAM function, when presenting the significantly changed ($P<0.01$) genes in the branch point. Significantly changed genes were classified with the 4 significantly changed gene modules and presented as a heatmap. Each gene modules were further utilized for GO enrichment analysis. Curve diagram displaying the relative expression levels of indicated genes or gene sets in CD34 lineage cells (Sham tdT and Ang II tdT group) along pseudotime differentiation trajectory were plotted with R package ggploT2 using geom_smooth function.

Cell-Cell Communication

To investigate the cell-cell communication between subpopulations and the differences in cell-cell communication between different groups, we used CellChat (https://github.com/sqjin/CellChat) R package, which allow the analysis of scRNA-seq data. Researchers can visualize the signaling pathways or ligand-receptor pairs between interest cell groups with "CellChat". Seurat preprocessed data was then subjected to CellChat package (version 1.1.3) to infer, analyze, and visualize cell-cell communication.[7] The hierarchy plot was first used to visualize the network structure between source cells and target cells for vital signaling pathways. The contribution of significant ligand-receptor pairs to the signaling pathway in the communication network was analyzed by calculating the relative ratio of communication strength of single ligand-receptor pair to that of the whole signaling pathway. With the combination of these tools, we were able to evaluate the interaction strength/weights between clusters in our scRNA-seq data and show them vividly in heatmap, circle or dot plots.

Correlation Analysis

To determine similarities between *Cd34*^{high} subset and *Pi16*^{high} subset in Sham SA and Ang II 4wks groups, we defined cell with rna_*Cd34* > 2 or rna_*Pi16*> 3 as *Cd34*^{high} or *Pi16*^{high} for fibroblasts, respectively. DEGs (FDR < 0.05, fold change >0.25 or < −0.25) for either comparison including *Cd34*^{high} versus *Cd34*^{low} and *Pi16*^{high} versus *Pi16*^{low} were used for Spearman correlation analysis. Visualization of the linear correlation analysis was performed by “ggscatter” function of the ggpubr package.

Mouse Vascular Adventitial CD34$^+$ Cell Isolation, Cell Culture and Differentiation

Mouse aortic stem/progenitor cells were isolated as previously described.[8] Briefly, the entire aorta was harvested and cut open. the adventitial layers were carefully dissected from the medial and intimal layers. The isolated aortic adventitia was then sliced into small pieces, seeded in a 0.04% gelatin (Sigma, G1393)-coated T25 flask and maintained in complete cell culture medium, which consists of DMEM (Sigma, D6429), 10% Fetal Bovine Serum FBS (HyClone, SH30396.03), 100 U/mL penicillin-streptomycin (Gibco, 15140122), 2% chick embryo extract (MP Biomedical), 100 nM retinoic acid (Sigma-Aldrich), 50 nM 2-mercaptoethanol (Sigma-Aldrich), 2% B27 (Invitrogen), 1% N2 (Invitrogen) and 20 ng /ml bFGF (R&D Systems). Cells derived from the outgrowth of aortic adventitia were then passaged for cell enrichment. Primary aortic cells were maintained in complete cell culture medium and passaged at a ratio of 1:3 every 3 days. Cell culture medium was changed every

other day. When expanded to the fifth generation, $CD34^+$ cells were sorted into DMEM with 20% FBS using a BD FACS ARIA II Flow Cytometer (BD Biosciences). The purified $CD34^+$ cells were expanded and cultured for subsequent experiments.

DMEM containing 10% FBS and 1% P/S was used to maintain $CD34^+$ cells in differentiation experiments. $CD34^+$ cells were serum-starved by culturing for 24 hours in DMEM without serum, and then treated with 200ng/mL PDGFBB (MCE, HY-P7087) for 12 hours to induce $CD34^+$ cells activation. Moreover, $CD34^+$ cells were also infected with Ad-*Pdgfrb* (10^8 pfu/ml) for 48 hours and treated with 12-hour 15μM LY294002 (inhibitor of PI3K) combined with 200ng/mL PDGFBB (MCE, HY-P7087) prior to any further analysis.

Co-culture system was established using transwell inserts with 0.4 μm pores and 6-well culture plates (Corning, CLS3450). Adventitial $CD34^+$ and $CD34^-$ cells were resuspended in 2.6 ml of 10% FBS-containing DMEM and seeded into the lower chamber at the same density (1×10^6 cells) respectively, and BM derived $CD34^+$ cells and $CD34^-$ cells were sorted and put into the upper chamber at the density of 1×10^6 cells. After 48 hours in co-culture, the upper chambers were removed, and the fibrosis-related protein expression of adventitial $CD34^+$ and $CD34^-$ cells were assessed by western blot.

Synthesis of Small Interfering RNAs (siRNAs) and Adenoviruses.

The siRNAs of *Pdgfra* and *Pdgfrb* were designed and synthesized by GenePharma Co.,Ltd., China. Under appropriate conditions, siRNAs and siCtrl were transfected into cells with Lipofectamine™ RNAiMAX Transfection Reagent (Invitrogen™, 13778075) following the manufacturer's instructions. The siRNA sequences used in this study shown in **Table S2**.

Recombinant adenoviruses expressing mouse PDGFRa (Ad-*pdgfra*), PDGFRb (Ad-*Pdgfrb*) and empty vector (Ad-vector) were constructed by GenePharma Co.,Ltd., China. Primers for sequencing shown in **Table S2**. Adenoviruses (10^8 PFU/ml) were used to infect adventitial cells for 48 hours.

Transwell Migration Assay.

Transwell migration assays were performed using transwell cell culture plates (Corning Costar, 3464) with 8.0-μm pore membrane filters. Cells (1×10^6 cells/100 μl serum-free medium) were seeded in the upper chamber, while 200 ng/ml PDGFBB in 600 μl serum free medium were then added to the lower chamber. After 18-hour incubation, medium from both chambers was discarded. Non-migrating cells remaining on the top of the transwell filters were removed by cotton swabs. Migrating cells on the lower surface of the transwell filter were fixed in 4% PFA for 10 minutes and stained with 1% crystal violet (Sigma, HT90132) for 15 minutes. Images were acquired using an Axioplan 2 imaging microscope. Cells were counted in 5 random fields under the microscope.

EdU Incorporation *in Vitro* and Staining

Cellular proliferation was measured by Cell-Light EdU Apollo488 In Vitro Kit (RiboBio, C10310-3) according to the manufacturer's instructions. Cells were seeded into 96-well plates (1.5×10^5 cells per well) and treated with DMEM containing 10% FBS, 1% P/S and 200 ng/mL PDGFBB. After 12-hour incubation with EdU (10 μM), Then, the cells were fixed in 100 ml of 4% formaldehyde for 15 minutes at room temperature (RT) and washed twice. Cells were then incubated with various concentrations of Triton X-100 and saponin for 15 minutes at RT and washed twice. For the EdU click reaction, the cells were treated with 200 μl of RiboBio's staining solution, incubated for 30 min at RT in the dark, washed and resuspended with PBS sequentially.

Gel Contraction Assay

Gel contraction assay was performed using Rat tail collagen type 1 assay (Good Laboratory Practice Bioscience, GC19587) according to the manufacturer's instructions. Cells were trypsinized and re-suspended with DMEM (382μl) mixed with 100 μl collagen type I (5mg/ml), 6 μl NaOH (0.1mol/L) and 12μl 10x PBS at 4 °C, total volume 500 ul. This 500 μl of cell-collagen mixture was plated into 24-well plates (2×10^5 cells per well) and incubated at 37 °C for 20 mins (for collagen polymerization). After the cell-collagen mixture solidifies, add appropriate volume of medium and transfer to the incubator for culture. After 48 hours, changes in the collagen gel size were observed after the gel was released from the side of the well.

RNA Extraction, Reverse Transcription, Quantitative Polymerase Chain Reaction (qPCR)

Total RNA from mouse aortic samples or cultured cells were extracted by Trizol reagent (Thermo Fisher Scientific, 15596018) and then reverse transcribed to generate cDNA using A RevertAid RT Reverse Transcription Kit (Thermo Fisher Scientific, K1691). All resultant cDNA was diluted to a working concentration of 2 ng/μl and stored at -20 °C. qPCR was performed using a TB green system (RR820A, TaKaRa Bio) on a CFX96 real-time system (BioRad). The primers used for qPCR analysis are listed in **Table S2**.

Immunoprecipitation (IP) Assays

For co-immunoprecipitation (co-IP) assays, Pierce™ Classic Magnetic IP/Co-IP Kit (88804, Thermo Scientific™) was used following the manufacturer's instructions. Briefly, $CD34^+$ adventitial cells were transfected with PDGFRb-overexpression adenovirus. After 48 hours, cells were lysed with IP lysis buffer (pH 7.4, 0.025M Tris, 0.15M NaCl, 0.001M EDTA, 1% NP40, 5% glycerol) containing 1 × protease inhibitor cocktail table (04693132001, Roche) and 1× phosphatase inhibitor PhosStop (4906837001, Roche). Samples were lysed on ice for 10 min and centrifuged at 13000 g for 10 min. The 500 μl supernatants were incubated with 1μg corresponding antibody at 4 °C overnight. Then the supernatants were incubated with 25 μl protein A/G magnetic beads at room temperature for 1 hour. The beads were washed with cold IP buffer for three times and incubated with elution buffer for 10 min. Then, the samples were boiled with 5 × loading buffer prior to Western blotting analysis. Antibodies used in IP assays: PDGFRa (#3164, Cell Signaling Technology), PDGFRb (#3169, Cell Signaling Technology), PI3K (#4292S, Cell Signaling Technology), IgG (#3900, Cell Signaling Technology). For IP-mass spectrometry analysis, the immunoprecipitation complex was separated by SDS-PAGE and then sent to Novogene Co., Ltd (Beijing, China) for analysis.

Western Blot

Human aortic samples and mice abdominal aortic samples were collected and homogenized in RIPA lysis buffer containing protease inhibitor cocktail (78425, Thermo Scientific, 1:100); Protein concentration was determined by BCA protein assay kit (23225, Thermo Scientific). Protein extracts were boiled at 95°C for 10 min in 1X loading buffer, then subjected to SDS-PAGE and transferred to PVDF membranes. Followed by blocking for 1h at room temperature in TBST with 5% no-fat milk, the membranes were incubated with primary antibodies at 4°C overnight. Following three washes in TBST buffer, the membranes were incubated with goat-anti rabbit IgG (Beyotime) conjugated with horseradish peroxidase (HRP), goat-anti mouse IgG (Beyotime) secondary antibodies for 1 hour at room temperature. The membranes were then washed three times in TBST buffer, and chemiluminescent signals were detected with Pierce ECL western blotting substrates and analyzed by Image Lab (v6.0). Relative protein levels were calculated by normalizing to GAPDH. The primary antibodies were used as listed: CD34 (Abcam, ab81829, 1:1000),αSMA (Abcam, ab21027, 1:1000), PDGFRa (R&D, AF1062, 1:1000), PDGFRb (R&D, AF1042, 1:1000), Periostin (R&D,AF2955, 1:1000), Vimentin (Abcam, ab8978, 1:1000), COL1A1 (Abcam, ab34710,

1:1000), p-PI3K (CST, #17366S, 1:500), PI3K (CST, #4292S, 1:700), AKT (CST, #4691S, 1:1000), p-AKT (Abcam, ab81283, 1:1000), PCNA (Abcam, ab29, 1:1000), GAPDH (Abcam, ab9485, 1:10000), HRP-Conjugated β-Actin (Proteintech, HRP-66009, 1:2000).

Statistical Analysis

GraphPad Prism 9.0 was used for statistical analysis and image creation. Numbers refer to independent experiments or mice as indicated in figure legends. Data are presented as mean±SD. For human and animal data, normality was tested by D'Agostino-Pearson (n=8 to 10) or Shapiro-Wilk (n=3 to 8) test. Normal distribution data between 2 groups were tested by unpaired 2-tailed t test, with Welch correction for unequal variance; nonparametric Mann-Whitney tests were used for abnormal distributions. For >2 groups, normal distribution data were tested by ordinary 1-way analysis of variance with Tukey test (equal SDs) or by Brown-Forsythe and Welch analysis of variance tests with Tamhane's T2 test (unequal SDs). Survival curves were analyzed by log-rank (Mantel-Cox) test. For cell culture data, 4 groups of data were first tested by Shapiro-Wilk test and then assessed by ordinary one-way ANOVA analysis (Tukey's multiple comparisons test). 6 groups of data were tested by 2-way ANOVA test. $p<0.05$ was considered to be statistically significant.

Representative images were chosen for high quality and accurate representation of quantitative analysis. Statistical calculations were performed on at least 3 independent experiments. scRNA-seq data were pooled from an average of 8 independent mice per group and integrated with a previously published scRNA-seq dataset from our group.

References:

[1] L. Jiang, T. Chen, S. Sun, R. Wang, J. Deng, L. Lyu, H. Wu, M. Yang, X. Pu, L. Du, Q. Chen, Y. Hu, X. Hu, Y. Zhou, Q. Xu, L. Zhang, *Circulation research* **2021**, *129* (8), e146.

[2] M. M. Bjørklund, A. K. Hollensen, M. K. Hagensen, F. Dagnaes-Hansen, C. Christoffersen, J. G. Mikkelsen, J. F. Bentzon, *Circulation research* **2014**, *114* (11), 1684.

[3] D. Yamanouchi, S. Morgan, C. Stair, S. Seedial, J. Lengfeld, K. C. Kent, B. Liu, *Journal of vascular surgery* **2012**, *56* (2), 455.

[4] T. Stuart, A. Butler, P. Hoffman, C. Hafemeister, E. Papalexi, W. M. Mauck, 3rd, Y. Hao, M. Stoeckius, P. Smibert, R. Satija, *Cell* **2019**, *177* (7), 1888.

[5] C. S. McGinnis, L. M. Murrow, Z. J. Gartner, *Cell Syst* **2019**, *8* (4), 329.

[6] G. Yu, L. G. Wang, Y. Han, Q. Y. He, *Omics* **2012**, *16* (5), 284.

[7] S. Jin, C. F. Guerrero-Juarez, L. Zhang, I. Chang, R. Ramos, C. H. Kuan, P. Myung, M. V. Plikus, Q. Nie, *Nat Commun* **2021**, *12* (1), 1088.

[8] K. Chen, R. Mou, P. Zhu, X. Xu, H. Wang, L. Jiang, Y. Hu, X. Hu, L. Ma, Q. Xiao, Q. Xu, *Circulation* **2023**, *147* (6), 482.

Figures S1–S20

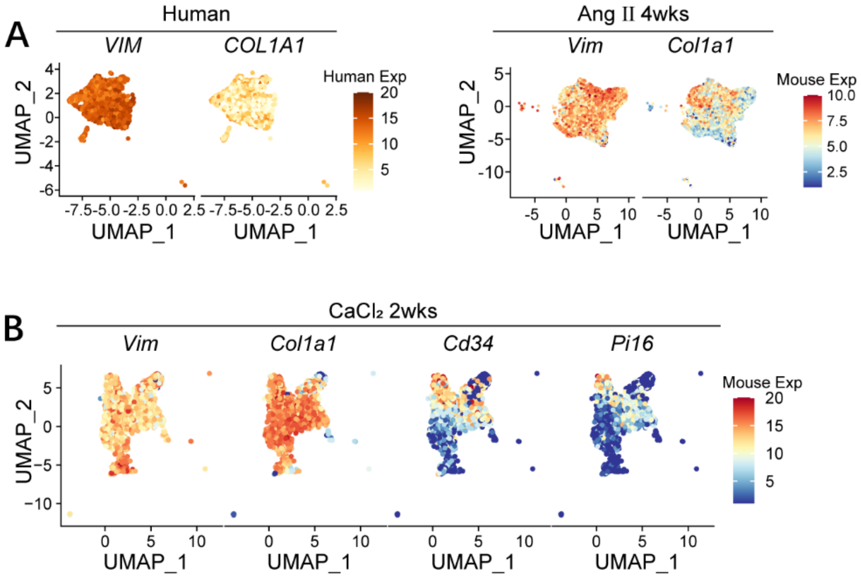

Figure S1 (Related to Figure 1). Gene expression profiles in fibroblasts in human and mouse abdominal aortic aneurysm. (A) Feature-plot showing the expression and distribution of fibroblast marker genes (*Vim/VIM*, *Col1a1/COL1A1*) in fibroblasts obtained from publicly available 10x single-cell RNA sequencing datasets of human (GSE155468 and GSE166676, left) and mouse (GSE221789, right) normal and aneurysmal aortas, respectively. (B) Feature-plot showing the expression and distribution of *Cd34*, *Pi16* and fibroblast marker genes in fibroblasts obtained from publicly available 10x single-cell RNA sequencing datasets of mouse normal and $CaCl_2$-induced aneurysmal aorta (GSE164678). Ang, angiotensin II.

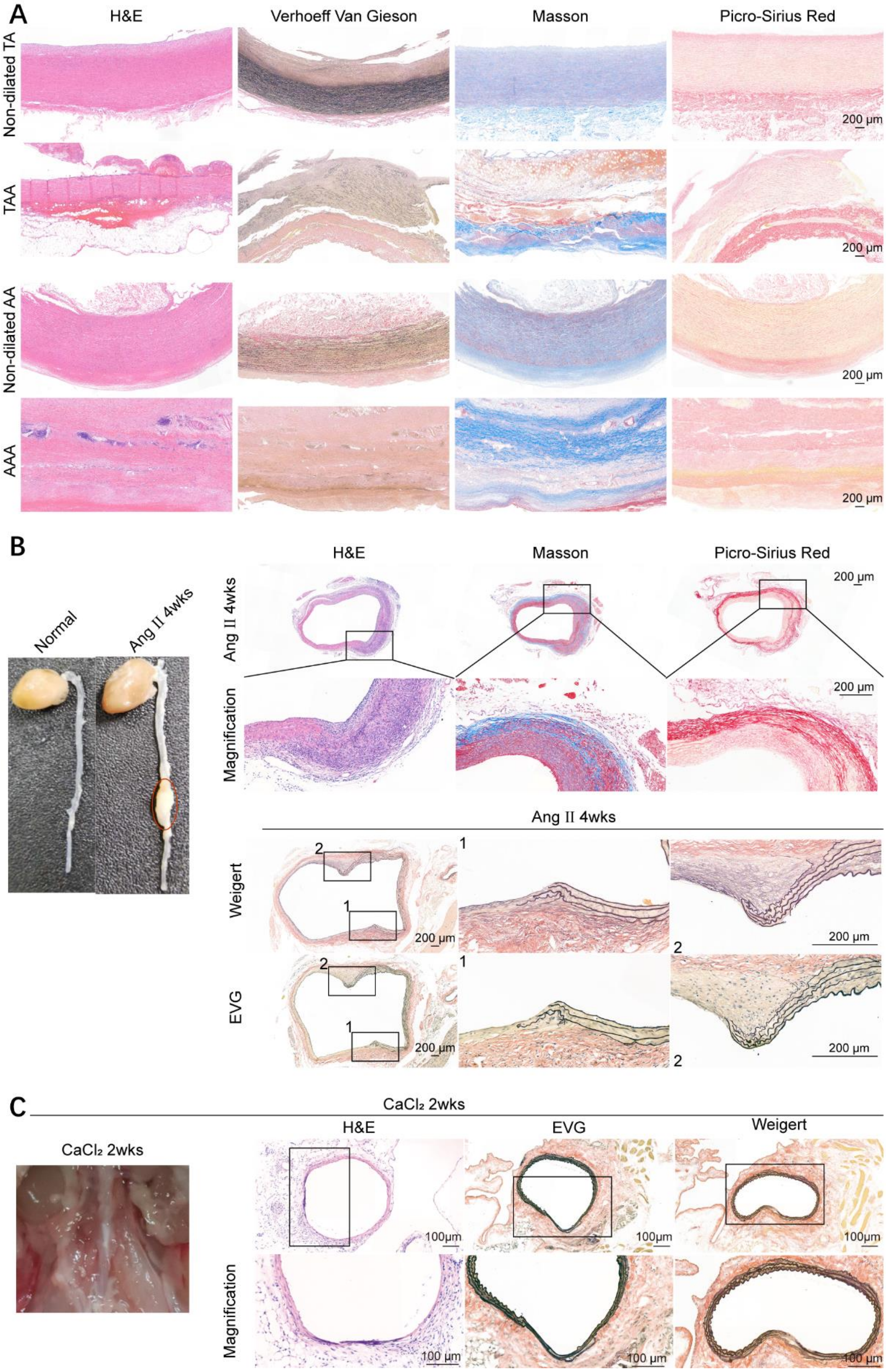

Figure S2 (Related to Figure 1). Histological characteristics of human and mouse abdominal aortic aneurysms. (A) Representative images of H&E, verhoeff (EVG), masson trichrome (Masson), and picrosirius red (PSR) staining of aortic wall from patients with thoracic aortic aneurysm (TAA), abdominal aorta aneurysm (AAA), and corresponding non-dilated aortic (Non-dilated TA and Non-dilated AA). (B) Left, gross images of the whole aortas harvested from mice infused with saline (normal) or Ang II for 4 weeks; Representative images

of H&E, Masson and PSR (right), as well as EVG and weigert (bottom) staining of suprarenal aortic wall of *Apoe*$^{-/-}$ mice treated with Ang II for 4 wks, with magnification of the boxed regions. (C) Left, gross images of the infrarenal aortas 2 weeks post $CaCl_2$ stimulation ($CaCl_2$ 2wks); Right, representative images of H&E, EVG, and weigert staining of infrarenal aortic wall of WT mice treated with $CaCl_2$ for 2 wks, with magnification of the boxed regions on the bottom. WT: wild type.

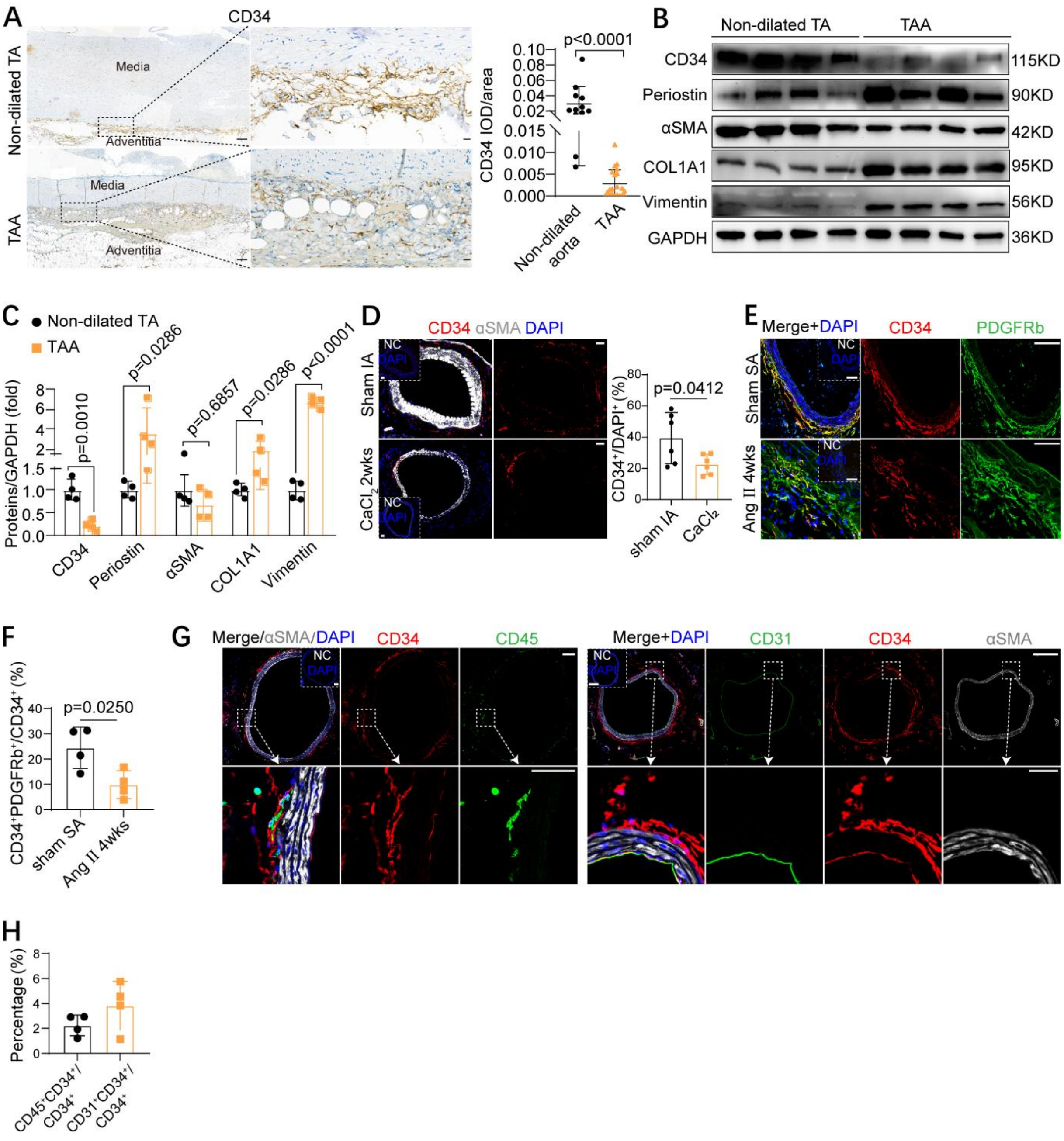

Figure S3 (Related to Figure 1). Expression patterns of CD34 in human thoracic aneurysm and mouse aortic aneurysm. (A) Representative immunohistochemical (IHC) images illustrating CD34 expression pattern in human TAA and non-dilated Thoracic aorta (Non-dilated TA), accompanied by magnified views of the boxed regions. Dot plot (right) displayed the average optical density of CD34-positive staining in aortic adventitia across different groups (n =12 and 21 individuals in Non-dilated TA and TAA group, respectively). Scale bar: 200 μm and 20 μm in the magnification regions. The presented data are expressed as mean±SD. Statistical analysis was conducted using the Mann-Whitney test. (B) Western blotting of CD34 and fibrosis-related proteins in aortas from patients with TAA and non-dilated aorta (Non-

dilated TA). N=4 individuals/group (upper). (C) Quantification of the indicated protein levels, normalized to GAPDH between human non-dilated TA and TAA (n=4 individuals/group). (D) Immunostaining for CD34 and αSMA on infrarenal aortic sections of WT mice subjected to PBS (Sham IA) or $CaCl_2$ incubation, with quantification of the percentage of CD34$^+$ cells in DAPI$^+$ cells (n=6 mice/group). Scale bar: 50 μm. IA: infrarenal aorta. (E) Immunostaining for CD34 and PDGFRb on aneurysmal suprarenal aortic sections of *Apoe*$^{-/-}$ mice treated with Ang II for 4wks. Scale bar: 50 μm. (F) Fluorescent quantification of the percentage of CD34$^+$PDGFRb$^+$ cells in CD34$^+$ cells (n=4 mice/group). (G) Immunostaining for CD34, CD31 or CD45, and αSMA on abdominal aortic sections of *Apoe*$^{-/-}$ mice at baseline, with magnification of the boxed region with split channels on the bottom. Scale bar: 200μm and 50 μm in magnification images. (H) Quantification of the percentage of CD34$^+$CD31$^+$ cells in CD34$^+$ cells, and CD34$^+$CD45$^+$ cells in CD34$^+$ cells (n=4 mice/group) For D, E and G, immunofluorescence-negative controls (NC) are shown in the corner. For C, D, F and H, data are expressed as mean±SD. Normal distribution data were tested by unpaired 2-tailed t test; nonparametric Mann-Whitney tests were used for abnormal distributions.

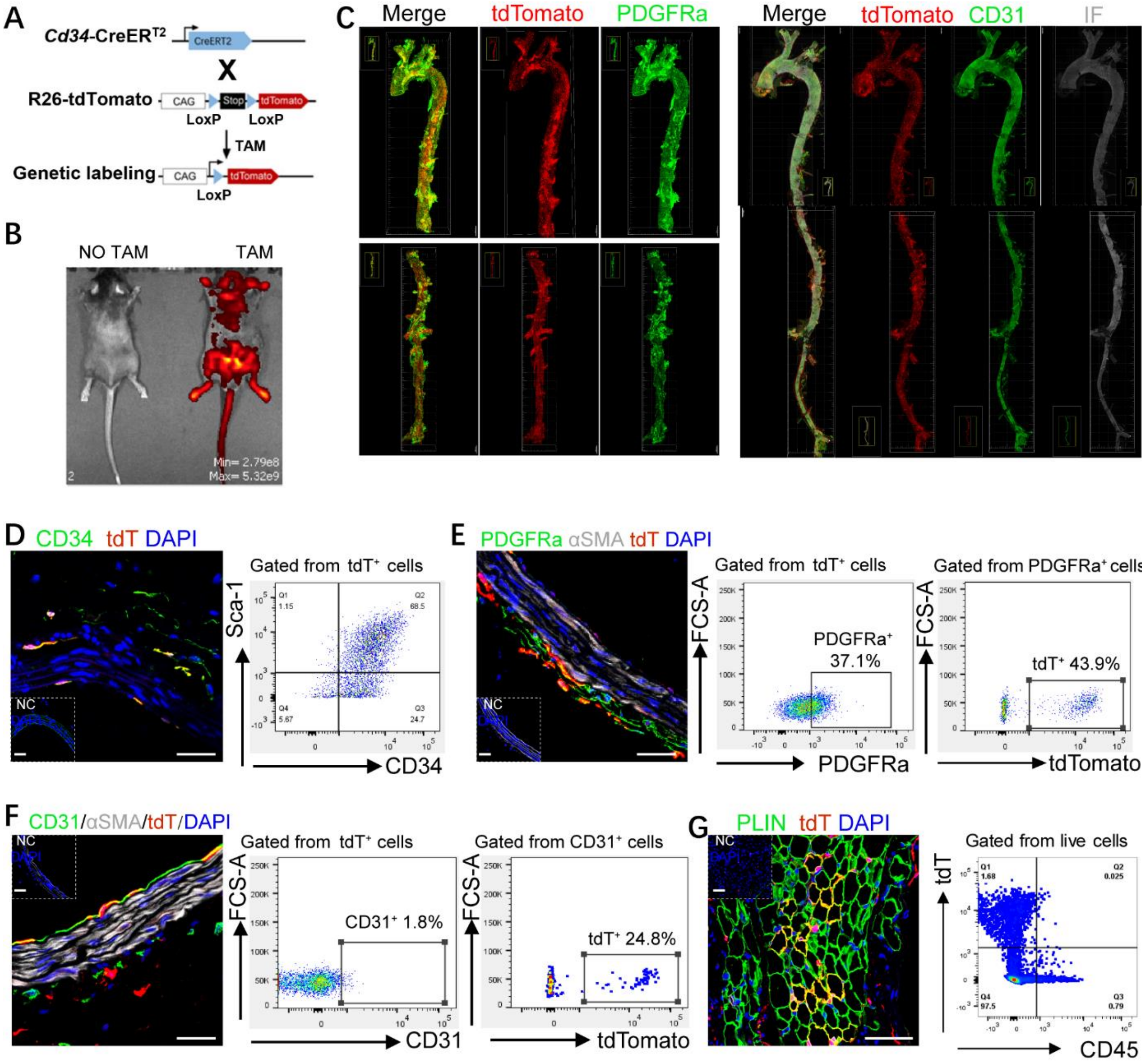

Figure S4 (Related to Figure 2). CD34$^+$ cellular lineage tracing under physiological condition. (A) Schematic image showing the generation of *Cd34*-CreER^{T2};R26-tdT mice, which were further cross-bred with *Apoe*$^{-/-}$ mice to obtain *Cd34*-CreER^{T2}; R26-tdT;*Apoe*$^{-/-}$ mice for Ang II-induced AAA model. (B) In vivo image showing the expression of tdTomato

in *Cd34*-CreER^{T2};R26-tdT;*Apoe*$^{-/-}$ mice with or without tamoxifen (TAM) treatment. (C) 3-dimensional reconstruction of immunostaining for tdTomato and PDGFRa (left) or CD31 (right) and IF (auto-fluorescence) on whole aortas of *Cd34*-CreER^{T2};R26-tdT;*Apoe*$^{-/-}$ mice under physiological condition. (D) Immunostaining for tdTomato (tdT) and CD34 on abdominal aortic sections (left), with flow cytometric quantification of CD34^{+} or Sca-1^{+} cells in tdT^{+} aortic cells isolated from *Cd34*-CreER^{T2};R26-tdT;*Apoe*$^{-/-}$ mice (right). (E) Immunostaining for tdT, αSMA and PDGFRa on abdominal aortas (left), with flow cytometric quantification of PDGFRa^{+} cells in tdT^{+} aortic cells (middle) as well as tdT^{+} cells in PDGFRa^{+} aortic cells (right) isolated from *Cd34*-CreER^{T2};R26-tdT;*Apoe*$^{-/-}$ mice. (F) Immunostaining for tdT, αSMA and CD31 on abdominal aortas (left), with flow cytometric quantification of CD31^{+} cells in tdT^{+} aortic cells (middle) as well as tdT^{+} cells in CD31^{+} aortic cells (right) isolated from *Cd34*-CreER^{T2};R26-tdT;*Apoe*$^{-/-}$ mice. (G) Immunostaining for adipocyte marker Perilipin A (PLIN) and tdT on periaortic tissue of *Cd34*-CreER^{T2};R26-tdT;*Apoe*$^{-/-}$ mice with TAM induction. (H) Flow cytometric analysis showing the percentage of tdT^{+} cells and CD45^{+} cells in single lived nucleated aortic cells isolated from *Cd34*-CreER^{T2};R26-tdT;*Apoe*$^{-/-}$ mice. For D-G, immunofluorescence-negative controls (NC) are shown in the corner. scale bar: 100 μm. Cell sample in each flow cytometric analysis were pooled cells of dissolved aortas from six *Cd34*-CreER^{T2};R26-tdT;*Apoe*$^{-/-}$ mice after TAM treatment.

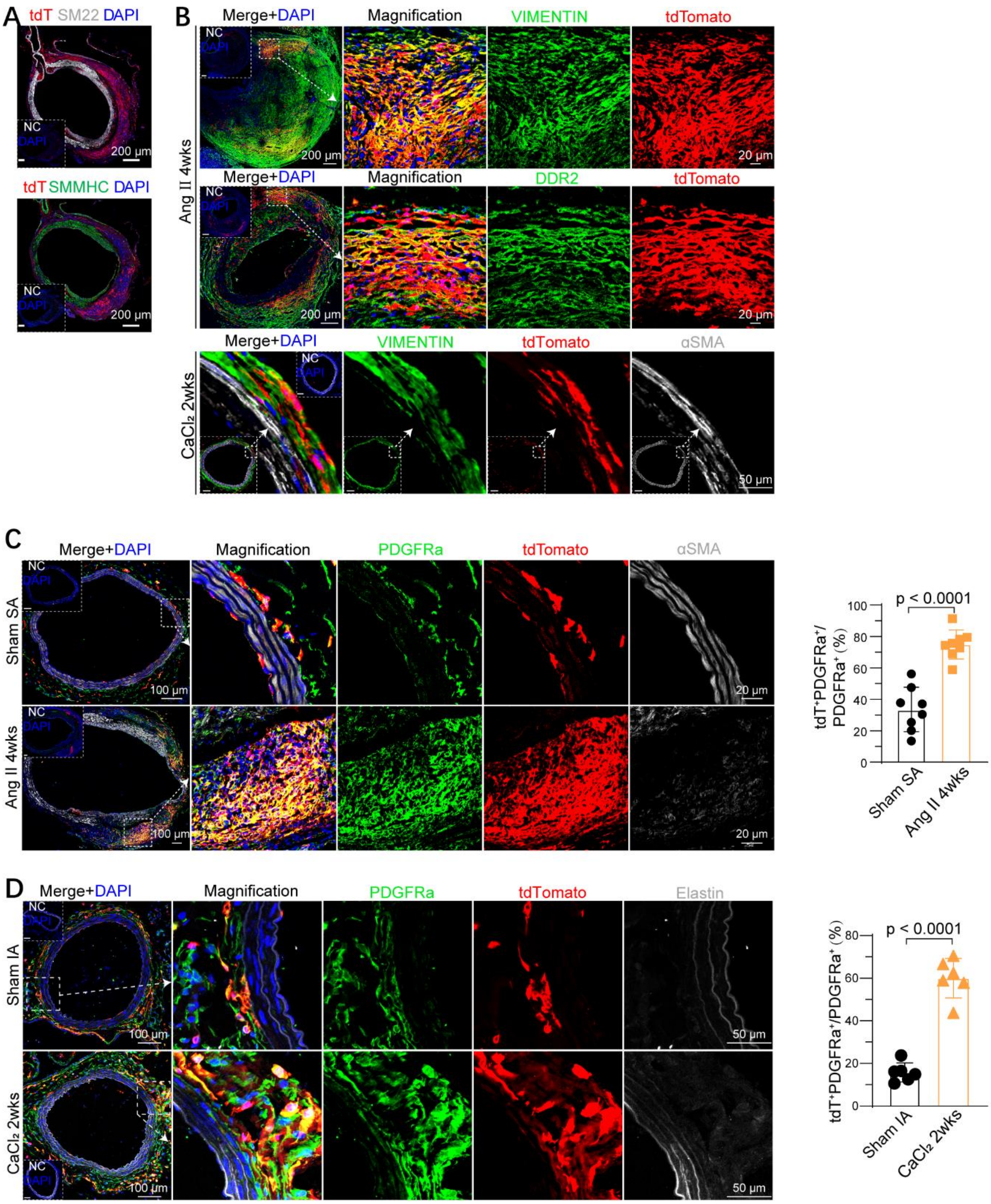

Figure S5 (Related to Figure 2). CD34$^+$ cells contributed to fibroblasts in the progression of AAA. (A) Immunofluorescence (IF) staining for tdTomato (tdT) and SM22 (upper) or SMMHC (bottom) on aneurysmal suprarenal aortic sections in the Ang II 4 wks group. (B) IF staining for tdTomato and Vimentin (upper) or DDR2 (bottom) on aneurysmal aortic sections in the indicated group. The boxed regions are magnified on the right with split channels. (C) IF staining for tdTomato, PDGFRa and αSMA on normal and aneurysmal suprarenal aortic sections in the Sham SA and Ang II 4wks group. The box regions are magnified on the right with split channels. Fluorescent quantification of the percentage of PDGFRa$^+$tdT$^+$ cells in PDGFRa$^+$ cells were displayed on the right (n=8 mice/group). (D) IF staining for tdTomato, PDGFRa and elastin on infrarenal aortic sections from *Cd34*-CreER^{T2};R26-tdT mice treated with $CaCl_2$ ($CaCl_2$ 2wks) or sham solution (Sham IA). The box regions are magnified on the right with split channels. Fluorescent quantification of the percentage of PDGFRa$^+$tdT$^+$ cells in

PDGFRa+ cells were displayed on the right (n=6 mice/group). *Cd34*-CreER^{T2};R26-tdT;*Apoe*$^{-/-}$ mice were used to model suprarenal abdominal aortic aneurysm induced by Ang II for 4 weeks. *Cd34*-CreER^{T2};R26-tdT mice were used for 2-week $CaCl_2$-incubated infrarenal aortic aneurysm. For C and D, data were first tested and passed normality test (Shapiro-Wilk test), and unpaired 2-tailed t tests (with homogeneity of variances tested) were performed.

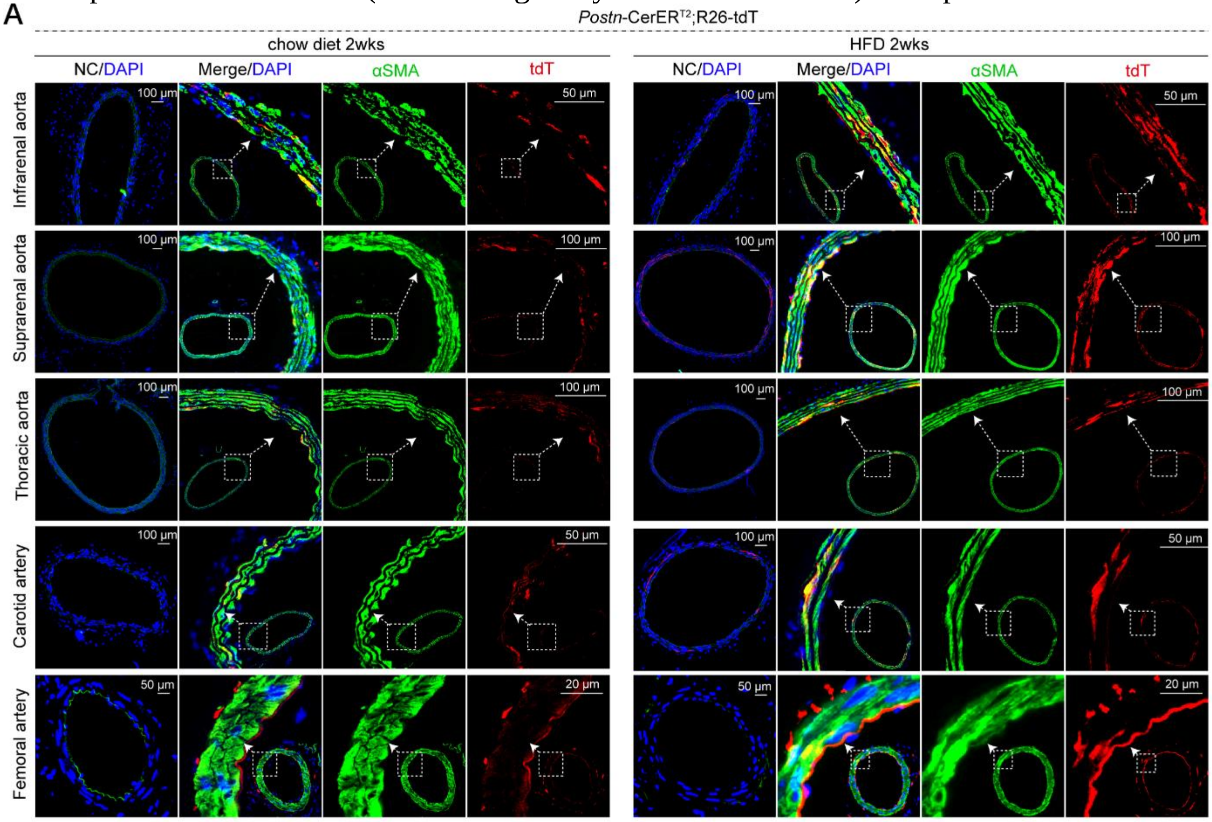

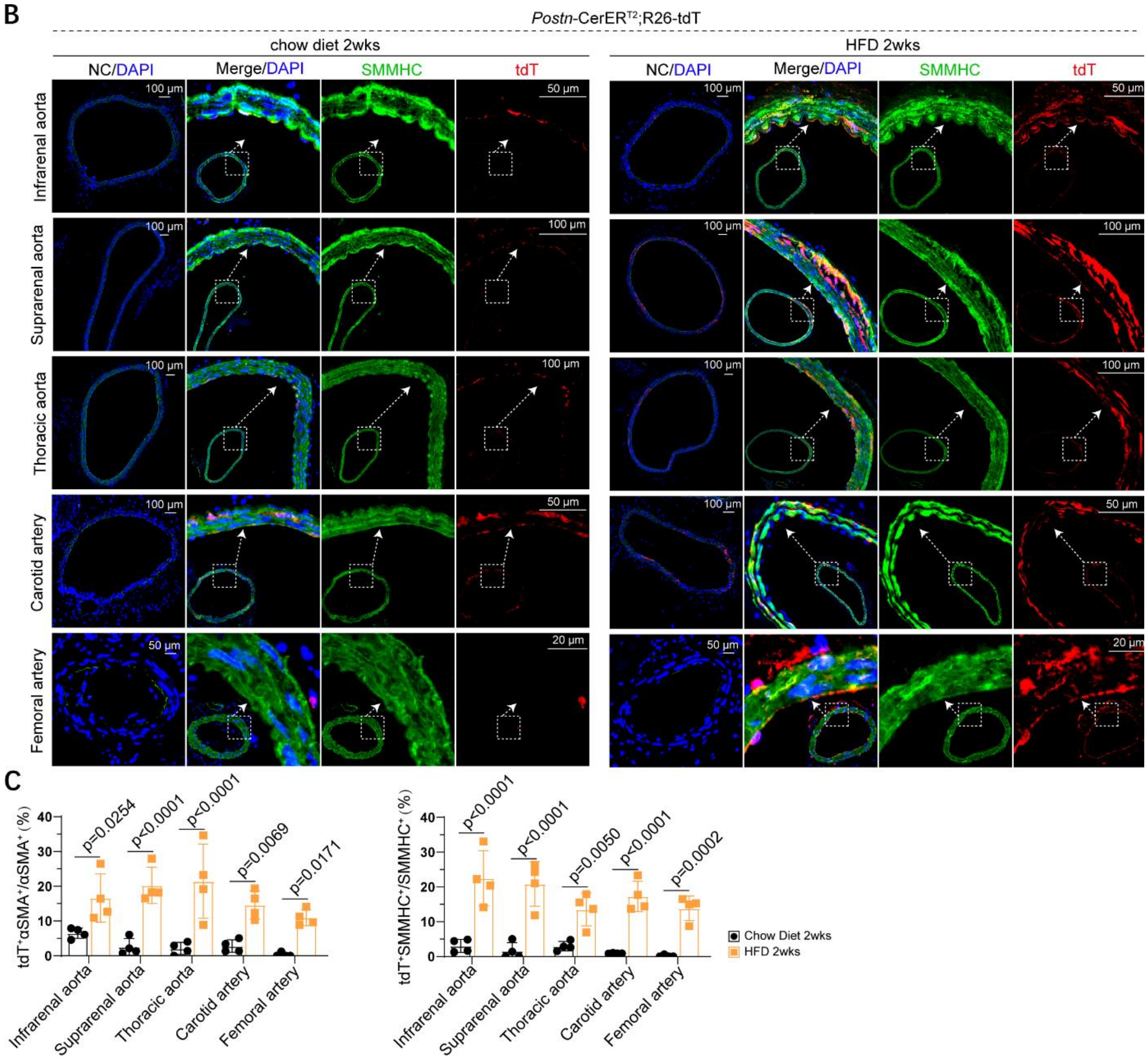

Figure S6 (Related to Figure2). Periostin$^+$ cellular lineage tracing in multiple arteries under physiological and HFD condition. (A, B) IF staining for αSMA (A) or SMMHC (B) and tdTomato (tdT) on frozen sections of infrarenal abdominal aorta, suprarenal abdominal aorta, thoracic aorta, carotid artery and femoral artery from 8-week-old *Postn*-CreER^{T2};R26-tdT mice fed with chow diet and HFD for 2weeks during TAM induction. (C) Quantification of the percentage of tdT$^+$αSMA$^+$ cells in αSMA$^+$ cells, and tdT$^+$SMHHC$^+$ in SMMHC$^+$ cells in the indicated group (n=4 mice/group). Data are expressed as mean±SD and are tested by 2-way ANOVA test.

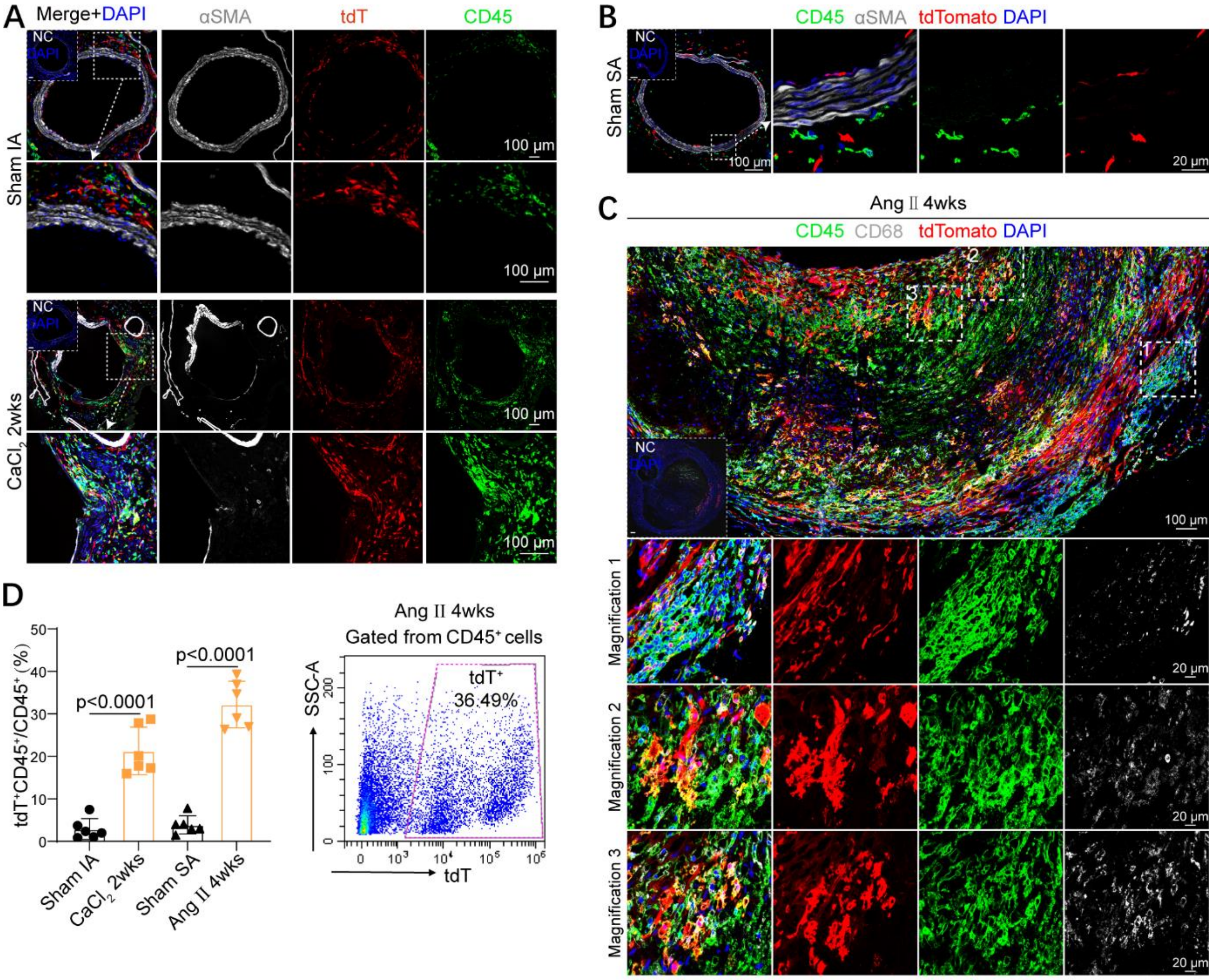

Figure S7 (Related to Figure2). Contributions of $CD34^+$ cells to immune cells in Ang II and $CaCl_2$-induced AAAs. (A) IF staining for tdT, CD45 and αSMA on infrarenal aortic sections from the indicated group, with magnification in the boxed region. (B) IF staining for tdT, CD45 and αSMA on suprarenal aortic section. (C) Immunostaining for tdTomato, CD45, and macrophage marker CD68 on abdominal aneurysmal aortic sections. The boxed regions are magnified on the bottom with split channels. (D) Fluorescent and flow cytometric quantification of the percentages of tdT^+ cells in $CD31^+$ endothelial cells. Aortic cells used in the flow cytometric analysis were pooled from six *Cd34*-CreER^{T2};R26-tdT; *Apoe*$^{-/-}$ mice infused with Ang II for 4 weeks. Data were first tested and passed normality test (Shapiro-Wilk test), followed by unpaired 2-tailed t tests.

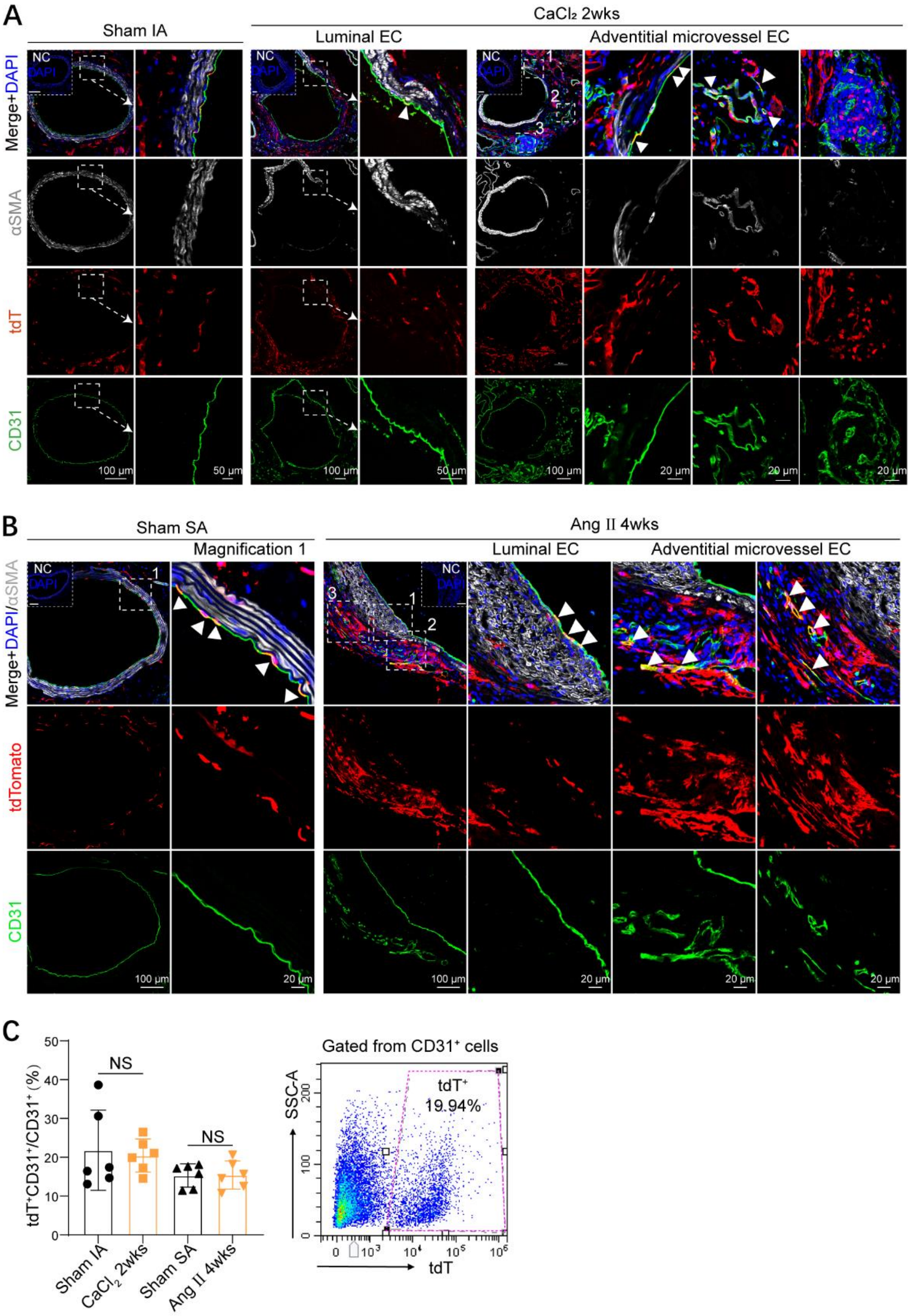

Figure S8 (Related to Figure2). Contributions of CD34$^+$ cells to ECs in Ang II and $CaCl_2$-induced AAAs. (A, B) IF staining for tdTomato (tdT), αSMA, and endothelial cell marker CD31 on aortic sections from the indicated group, with split-channel magnified views of the boxed regions. Arrow heads indicate tdT$^+$CD31$^+$ ECs. (C) Fluorescent and flow cytometric quantification of the percentages of tdT$^+$CD31$^+$ cells in CD31$^+$ endothelial cells (n=6 mice/group). Data were tested by unpaired 2-tailed t test.

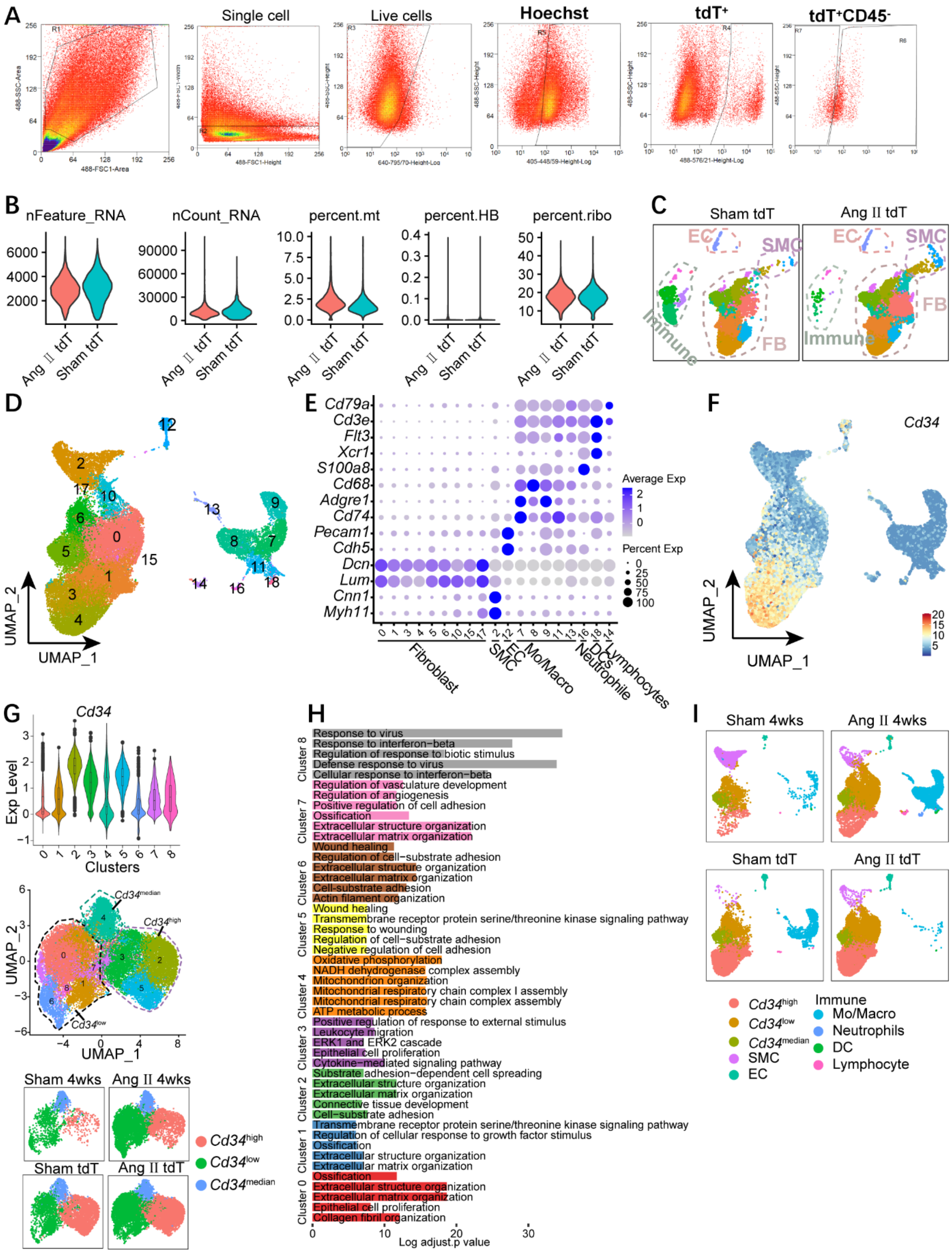

Figure S9 (Related to Figure 2). Integrated single-cell RNA sequencing analysis of aortic aneurysmal cells. (A) Representative gating strategy for sorting tdTomato (tdT)$^{+}$CD45^{-} cells digested from aneurysmal aortas of *Cd34*-CreER^{T2};R26-tdT *Apoe*$^{-/-}$ mice infused with saline (Sham tdT) or Ang II (Ang II tdT) for 4 weeks after TAM injection. (B) Violin plots showing the distributions for number of genes (nFeature_RNA and nCount_RNA) detected in all cells, and the percentages of mitochondrial genes (percent mt), haemoglobin genes (percent HB) and ribosomal genes (percent ribo) from Sham tdT and Ang II tdT datasets after filtering. Cells

expressing <400 or >7500 genes were filtered out to exclude noncell or cell aggregates, respectively. Cells with >10% mitochondrial gene percentage and with < 5% ribosomal gene percentage were also filtered out to exclude cells at a compromised state. (C) Split view of UMAP plot of 17,178 cells from Sham tdT group (6541cells) and Ang II tdT group (10637 cells), colored by cell type. (D) UMAP plot of integrated scRNA-seq datasets of tdTomato^{+}CD45^{-} aortic cells harvested from Sham tdT and Ang II tdT groups, as well as of whole abdominal aortic cells isolated from *Apoe*$^{-/-}$ mice infused with saline (Sham 4wks) or Ang II (Ang II 4wks) for 4 weeks, colored by clusters. (E) Dotplots showing marker genes expressions for fibroblasts, endothelial cells (ECs), smooth muscle cells (SMCs), monocytes/macrophages (Mo/Macro), neutrophils, dendritic cells (DCs), and lymphocytes in all cell clusters. (F) Feature-plot showing *Cd34* gene expression in all aortic cells. (G) Nine fibroblast clusters, which were classified into *Cd34*^{high}, *Cd34*^{median} and *Cd34*^{low} fibroblasts according to the expression level of *Cd34* gene. Violin plot of the expression level of Cd34 gene in each fibroblast sub-cluster (upper). Exp level, expression level. Total UMAP plot of fibroblasts (middle) and split view of UMAP plots of *Cd34*^{high}, *Cd34*^{median} and *Cd34*^{low} fibroblasts in indicated groups (bottom). (H) Bar plots showing representative gene ontology biological functions enriched in 9 fibroblast sub-clusters. (I) Split view of UMAP plot showing all aortic cells in each dataset, colored by cell types.

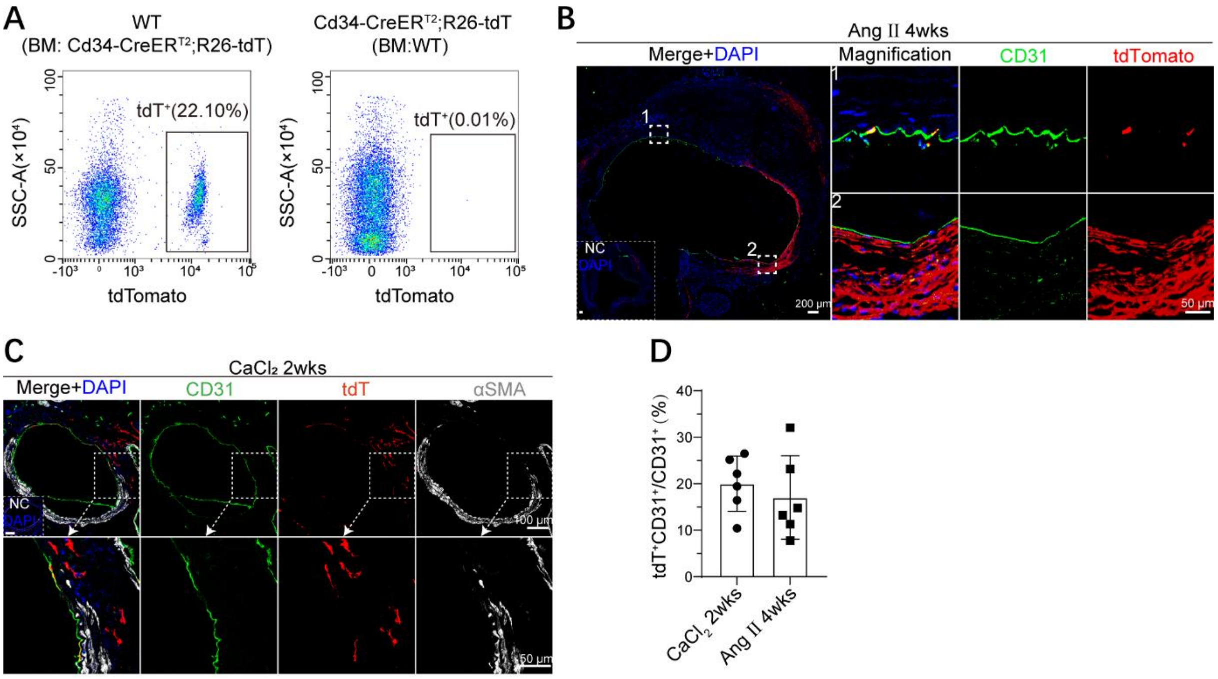

Figure S10 (Related to Figure 3). Limited contribution of non-bone marrow CD34^{+} cells to endothelial regeneration in AAA. (A) Flow cytometric analysis of tdTomato of BM cells from the indicated chimeric mice after TAM induction. (B) Immunostaining for tdTomato and CD31 of suprarenal aneurysmal aortic sections from Ang II-infused chimeric BMT$^{Apoe-/-\rightarrow Cd34}$ mice (BM: *Apoe*$^{-/-}$). (C) Immunostaining for tdTomato and CD31 of infrarenal aneurysmal aortic sections from $CaCl_2$-treated chimeric BMT$^{WT\rightarrow Cd34}$ mice (BM: WT). Scale bar, 100 μm and 50 μm in the magnification regions. (D) Quantification of the percentage of tdT^{+}CD31^{+} cells in CD31^{+} endothelial cells (n=6 mice/group).

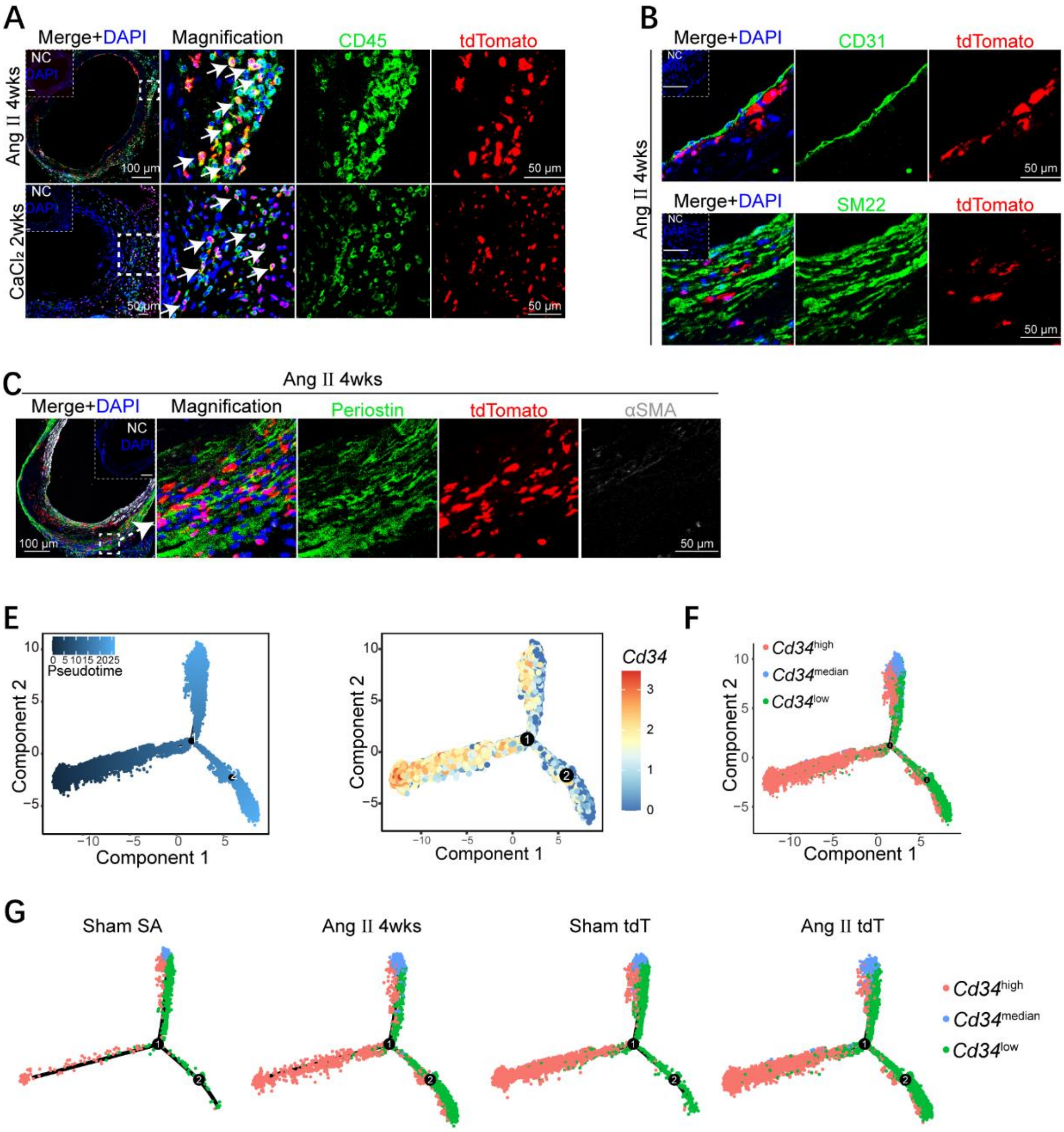

Figure S11 (Related to Figure 3). BM-derived CD34^{+} cells generated immune cells during AAA progression. (A) Immunostaining for tdTomato and CD45 of aneurysmal aortic sections from the indicated group. (B) Immunostaining for tdTomato and endothelial cell marker CD31 (upper) or SMC markers SM22 (bottom) on aneurysmal suprarenal aortic sections of chimeric BMT$^{Cd34 \rightarrow Apoe-/-}$ mice (BM: *Cd34*). (C) Immunostaining for Periostin, tdTomato, and αSMA on aneurysmal suprarenal aortic sections of chimeric BMT$^{Cd34 \rightarrow Apoe-/-}$ mice (BM: *Cd34*). (E) Pseudotime trajectory analysis of all fibroblasts of 4 groups, color by pseudotime (left), and relative expression level of *Cd34* among pseudotime trajectory (right). (F) Pseudotime trajectory analysis of all fibroblasts, color by cell clusters. (G) Split view of trajectory analysis of each group, color by cell clusters. Please refer to **Figure** 2J for the detailed description for experimental groups (Sham tdT, Ang II tdT, Sham 4wks, Ang II 4wks).

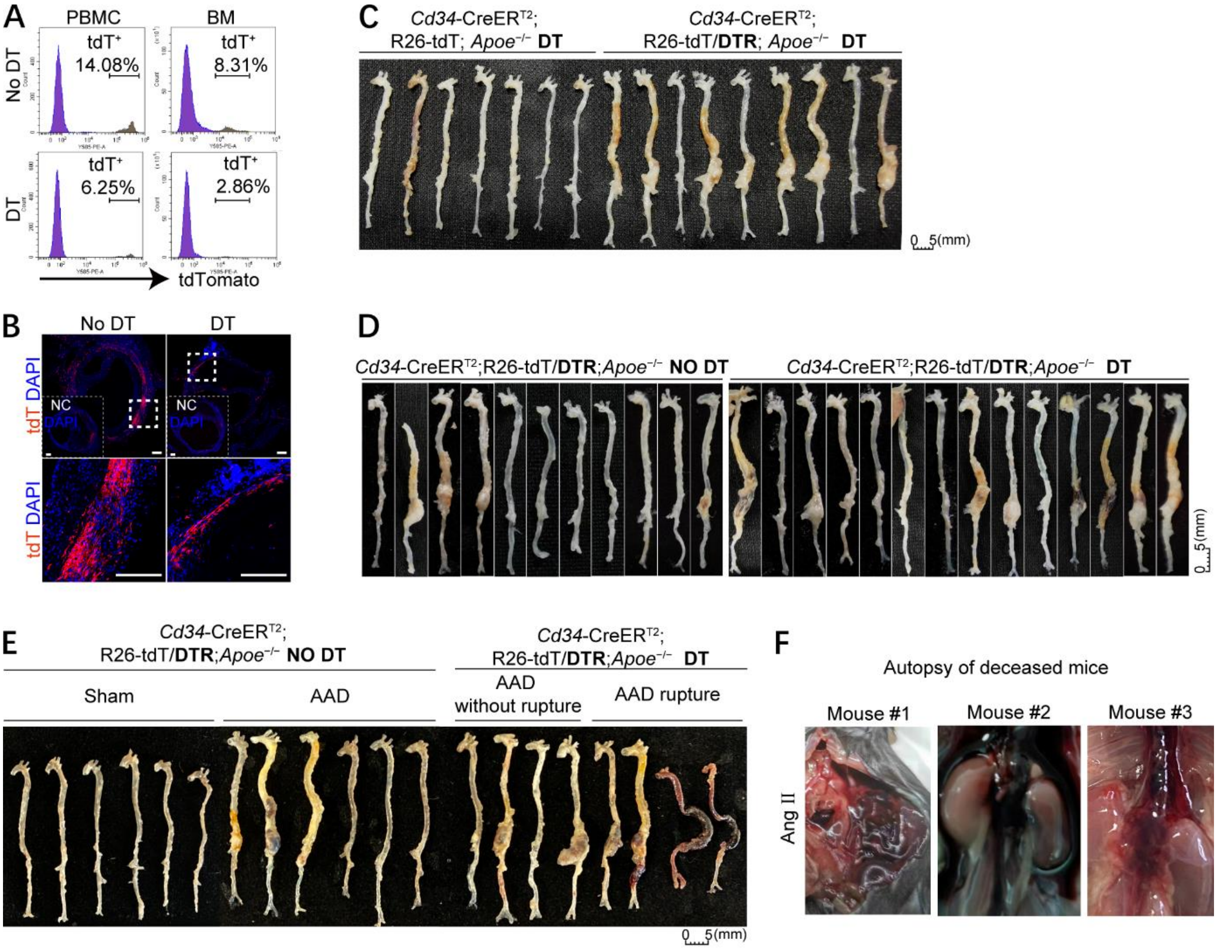

Figure S12 (Related Figure 4). Verification of efficiency of cell deletion and cause of deceased mice in *Cd34*-CreER^{T2};R26-tdTomato/DTR;*Apoe*$^{-/-}$ mice (DTR mice). (A) Flow cytometric analysis for tdTomato-expressing (tdT^{+}) cells in bone marrow (BM) cells and peripheral blood monocytes (PBMCs) from tamoxifen (TAM)-induced DTR mice with (DT) or without (No DT) diphtheria toxin (DT) treatment. (B) Immunostaining for tdTomato (tdT) of aneurysmal suprarenal aortic sections from DTR mice within indicated groups. Quantification of the effeciency of CD34^{+} cells deletion was shown in **Figure** 4L. (C) Gross images of the whole aortas harvested from *Cd34*-CreER^{T2};R26-tdT;*Apoe*$^{-/-}$ mice and DTR mice treated with DT. (D) Gross images of the whole aortas harvested from DTR mice treated with DT or without DT. (E) Gross images of the whole aortas harvested from mice in the indicted groups for in-vivo ultrasound monitor and quantification of maximal diameters. (F) Anatomical images showed a substantial volume of intra-abdominal blood in deceased mice, with aorta dissected from surrounding tissue.

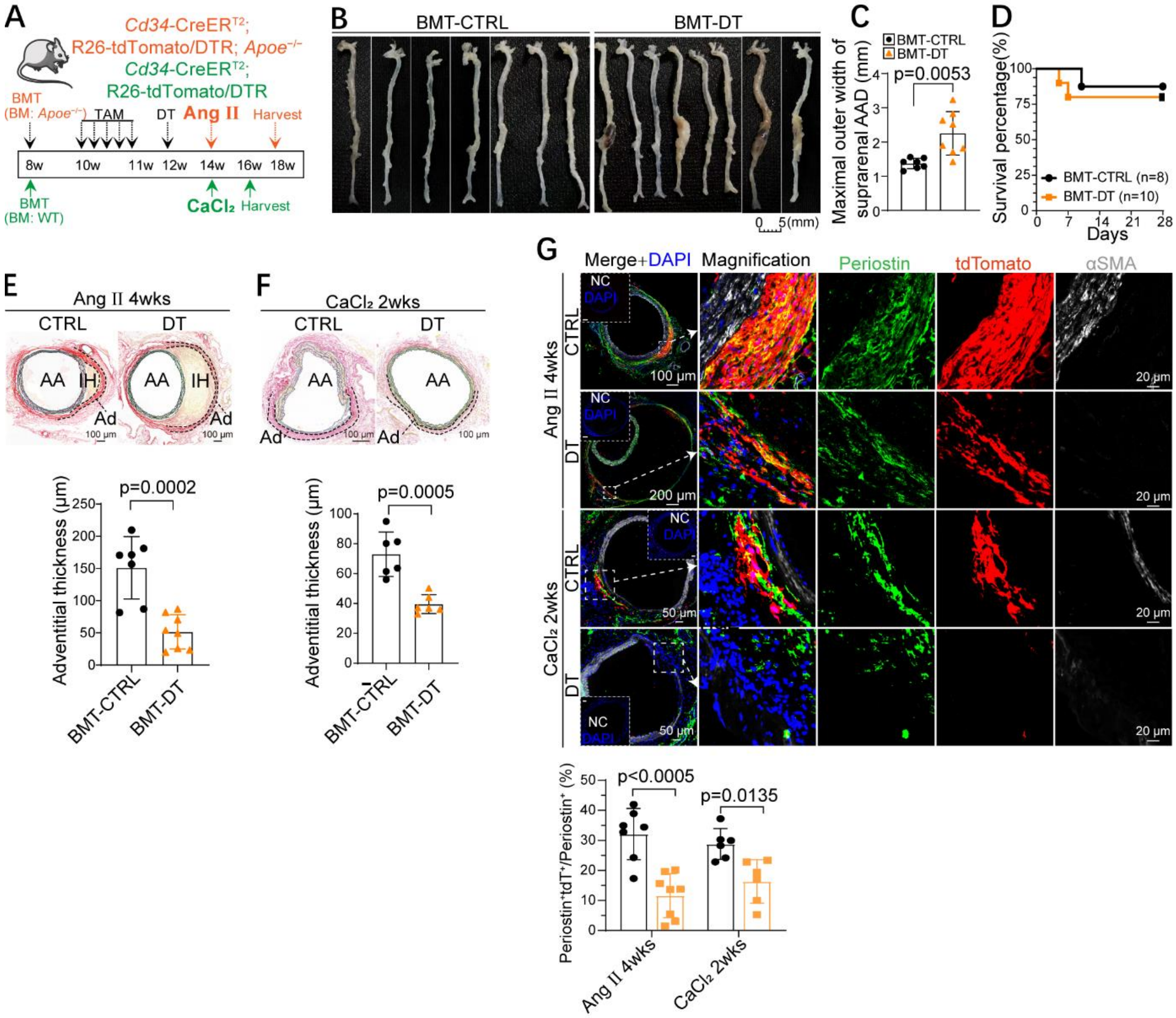

Figure S13 (related to figure 4) Deletion of non-BM CD34⁺ cells potentiated AAA development and attenuated the adventitial fibrous collar. (A) Sketch of the experimental design for non-BM CD34⁺ cell deletion in *Cd34*-CreER^{T2};R26-tdTomato/DTR;*Apoe*$^{-/-}$ or *Cd34*-CreER^{T2};R26-tdTomato/DTR mice respectively using BM transplantation (BMT), DT injection, and Ang II induction (orange) or $CaCl_2$ incubation (green), respectively. (B) Gross images of the whole aortas harvested from DTR mice after BMT with (BMT-DT) or without (BMT-CTRL) non-BM CD34⁺ cell deletion. (C) Quantification of the MED of suprarenal aortic aneurysms displayed in the indicated group. (D) Survival curve showing the percentages of survival mice treated with Ang II induction for 4 weeks in the indicated group. Data were tested by log-rank (Mantel-Cox) test. (E) Representative images of PSR-VB staining showing aneurysmal suprarenal aortic sections from BMT mice with (BMT-DT) or without (BMT-CTRL) non-BM CD34⁺ cell deletion after Ang II induction for 4 weeks (n=7-8 mice per group). Quantification of adventitial thickness shown in the bottom. (F) Representative images of PSR-VB staining showing aneurysmal infrarenal aortic sections from BMT mice with (BMT-DT) or without (BMT-CTRL) non-BM CD34⁺ cell deletion after $CaCl_2$ stimulation for 2 weeks (n=6 mice per group). Quantification of adventitial thickness shown in the bottom. (G) IF staining for Periostin, tdTomato and aSMA on aneurysmal aortic sections of the indicated groups. The box regions are magnified on the right. Quantification of the percentage of Periostin⁺tdT⁺ cells in Periostin⁺ cells shown in the bottom (n=6-8mice/group). For C, data were presented as mean ± SD and were first tested by Shapiro-Wilk test for normality, and then unpaired t test with Welch's correction was performed. For E and F, data were first tested by Shapiro-Wilk test for normality and unpaired t test was performed. For G, two way ANOVA analysis was performed.

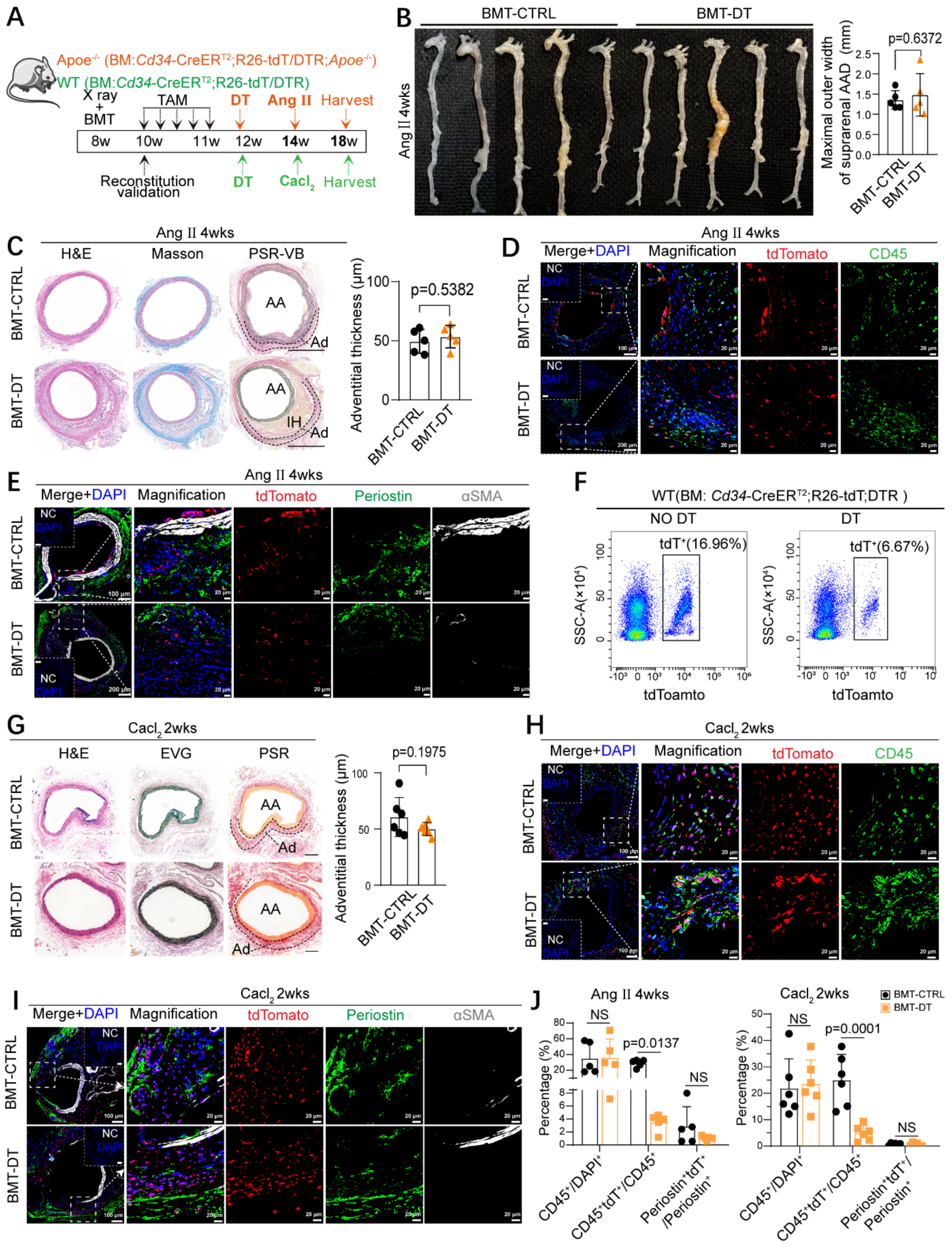

Figure S14 (Related Figure 4). Deletion of BM CD34$^+$ cells did not affect the thickness of adventitial fibrous collar. (A) Schematic diagram showing the experimental design for depletion of bone marrow derived CD34$^+$ cells and Ang II-induced suprarenal AAA in *Cd34*-CreER^{T2};R26-tdTomato/DTR;*Apoe*$^{-/-}$ mice. (B) Left, gross images of the whole aortas harvested from each mouse within indicated groups. Scale bar: 3 mm. Right, quantification of the maximal external diameters (MED) of suprarenal aortic aneurysms in the indicated group (n=5 mice/group). (C) Left, representative images of H&E, Masson and PSR-VB staining

showing aneurysmal suprarenal aortic sections of CD34+ cell deletion group (BMT-DT) and non-deletion group (BMT-CTRL) after Ang II induction for 4 weeks (n=5 mice per group). Scale bar: 200 µm. Quantification of adventitial thickness shown in the right. Adventitial thickness was assessed and quantified by PSR-VB staining for the thickness of the red fibrotic area marked by dotted line in the adventitial layer. (D) Immunofluorescence (IF) staining for CD45 and tdTomato on aneurysmal aortic sections in the indicated group. The box regions are magnified on the right with split channels. (E) IF staining for Periostin, tdTomato and αSMA on aneurysmal aortic sections in the indicated group. The box regions are magnified on the right with split channels. (F) Schematic diagram showing the experimental design for $CaCl_2$ incubation model and bone marrow derived CD34+ depletion in *Cd34*-CreER^{T2};R26-tdTomato/DTR mice. (G) Flow cytometric analysis of tdTomato of BM cells from the chimeric BMT$^{(BM: CD34)}$ mice with or without DT injection after TAM induction. (H) Left, representative images of H&E, EVG and PSR staining showing aneurysmal infrarenal aortic sections of CD34+ cell deletion group (BMT-DT) and non-deletion group (BMT-CTRL) after $CaCl_2$ induction for 2 weeks (n=6 mice per group). Scale bar: 100 µm. Quantification of adventitial thickness shown in the right. (I) IF staining for Periostin (left) and CD45 (right), tdTomato and αSMA on aneurysmal aortic sections in the indicated group. The box regions are magnified on the right with split channels. (J) Quantification of the percentage of CD45+ cells in DAPI+ cells, CD45+tdT+ cells in CD45+ cells and Periostin+tdT+ cells in Periostin+ cells in the indicated group. For B, C, G and J, data represent mean±SD. Data were first tested by Shapiro-Wilk test for normality and unpaired t test was performed for B and C. Unpaired t test with Welch's correction was performed for G. Data was tested by two-way ANOVA analysis in J.

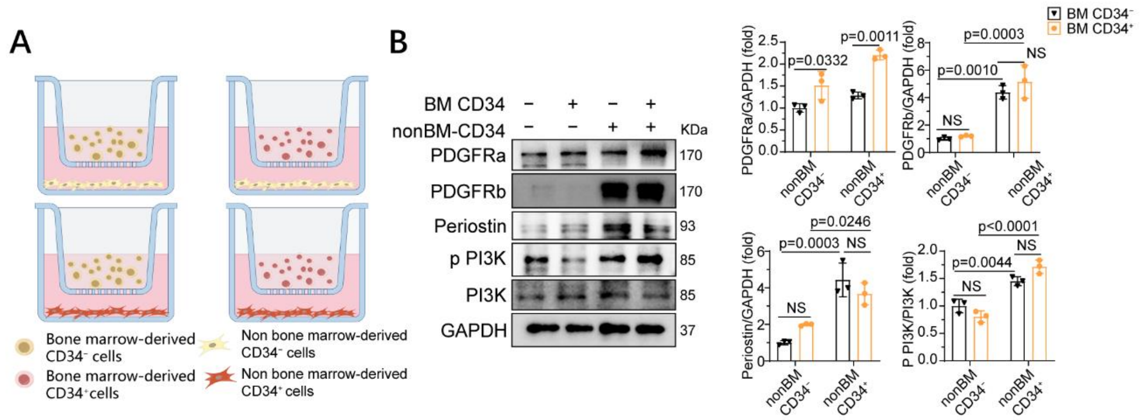

Figure S15 (Related to Figure 4). Co-culture experiments for BM cells and aortic adventitial fibroblasts. (A) Schematic procedure for co-culture assay. The non bone marrow-derived CD34- and CD34+ cells were respectively co-cultured with bone marrow-derived CD34- and CD34+ cells for 48h. (B) Western blot illustrating PDGFRa, PDGFRb, Periostin, pPI3K and PI3K protein level of non bone marrow-derived CD34- and CD34+ cells. GAPDH served as a loading control. Quantification (right) for the western blots of PDGFRa, PDGFRb, Periostin and pPI3K proteins in indicated cells. The results present one of three independent experiments.

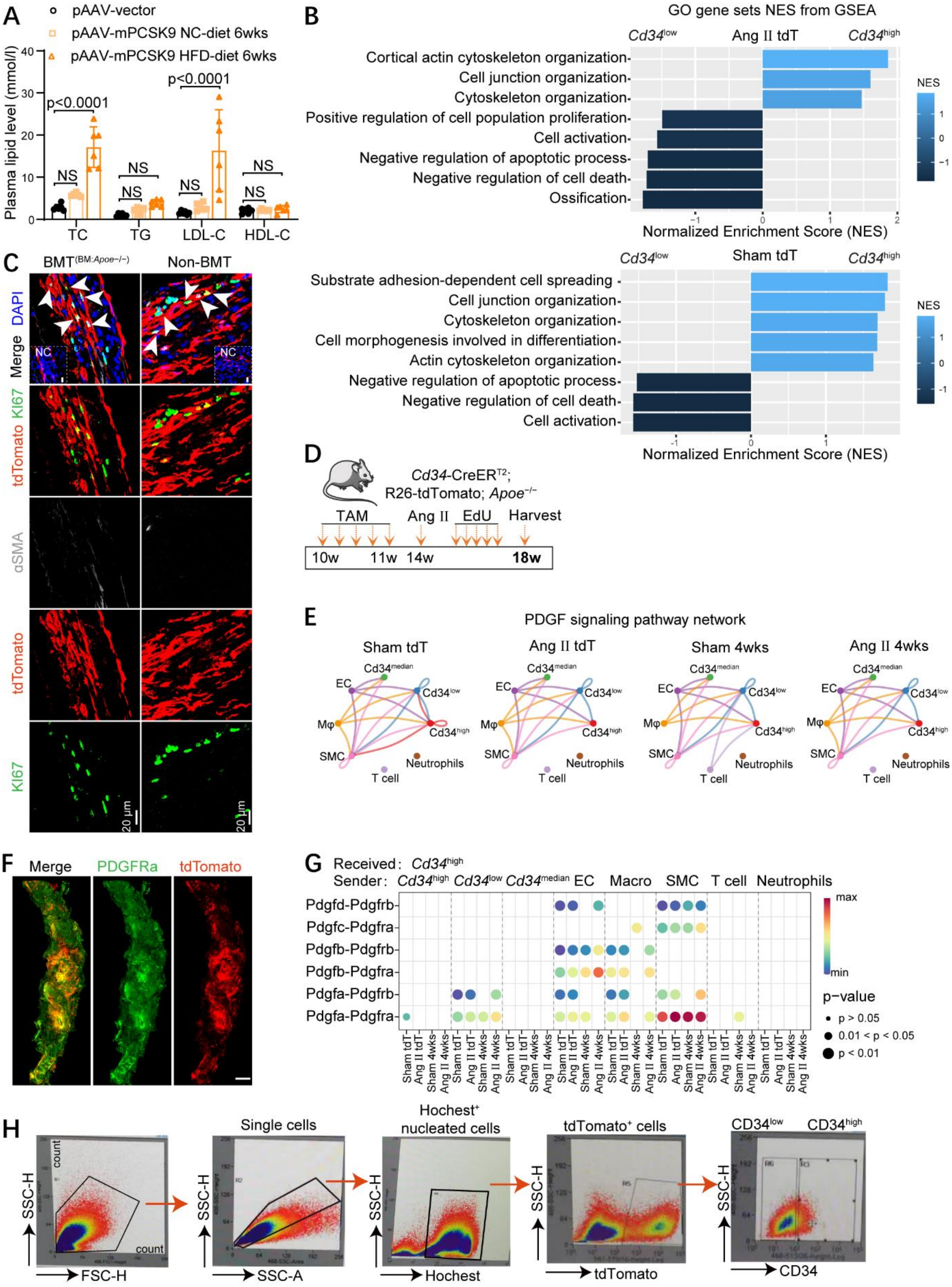

Figure S16 (Related Figure 5). PDGF signaling mediates CD34^{+} cell activation. (A) Plasma concentration of total cholesterol (TC), triglyceride (TG), low-density lipoprotein cholesterol (LDL-C), and high-density lipoprotein cholesterol (HDL-C) in *Cd34*-Dre;*Postn*-CreER^{T2}; Dou-tdT-DTR mice injected with AAV (adeno-associated virus)-vector or AAV-PCSK9 (proprotein convertase subtilisin/kexin type 9), and fed a high-fat (HFD) or normal chow (NC) diet for 4 weeks, respectively. Data represent mean±SD, and were tested by 2-way ANOVA test

(Dunnett's multiple comparisons test). (B) GSEA analysis and bar plots showing enriched GO pathways in *Cd34*^{high} and *Cd34*^{low} aortic cells in Sham tdT or Ang II tdT group. (C) Immunofluorescence (IF) staining for cellular proliferation marker KI67, tdTomato and αSMA on aneurysmal abdominal aortic sections from non-BMT (right) and BMT$^{BM:Apoe-/-}$ mice (left) after 4-wks Ang II induction. Scale bar: 50 µm. Arrow heads indicate tdT^{+}KI67^{+} fibroblasts. (D) Schematic diagram showing the experimental design for EdU (5-Ethynyl-2'-deoxyuridine) injection to label proliferating cells during AAAs progression. (E) Chord diagram of PDGF signaling network among all cell types in indicated groups. Please refer to Figure 2J for the detailed description for experimental groups (Sham tdT, Ang II tdT, Sham 4wks, Ang II 4wks). (F) 3-demesional reconstruction of IF staining for tdTomato and PDGFRa in aneurysmal abdominal aortas of *Cd34*-CreER^{T2};R26-tdT;*Apoe*$^{-/-}$ mice after Ang II induction for 4 wks. Scale bar, 1 mm. (G) Comparison of the ligand-receptor pairs of PDGF signaling among all four groups (Sham tdT, Ang II tdT, Sham 4wks, and Ang II 4wks, as described in Figure 2J) between *Cd34*^{high} cells (as the receiver cells) and other aortic cells (as signal-sending cells). (H) Representative gating strategy for sorting tdTomato^{+}*Cd34*^{high} and tdTomato^{+}*Cd34*^{low} subpopulations isolated from aortic adventitia of *Postn*-CreER^{T2};R26-tdT mice after TAM and Ang II induction.

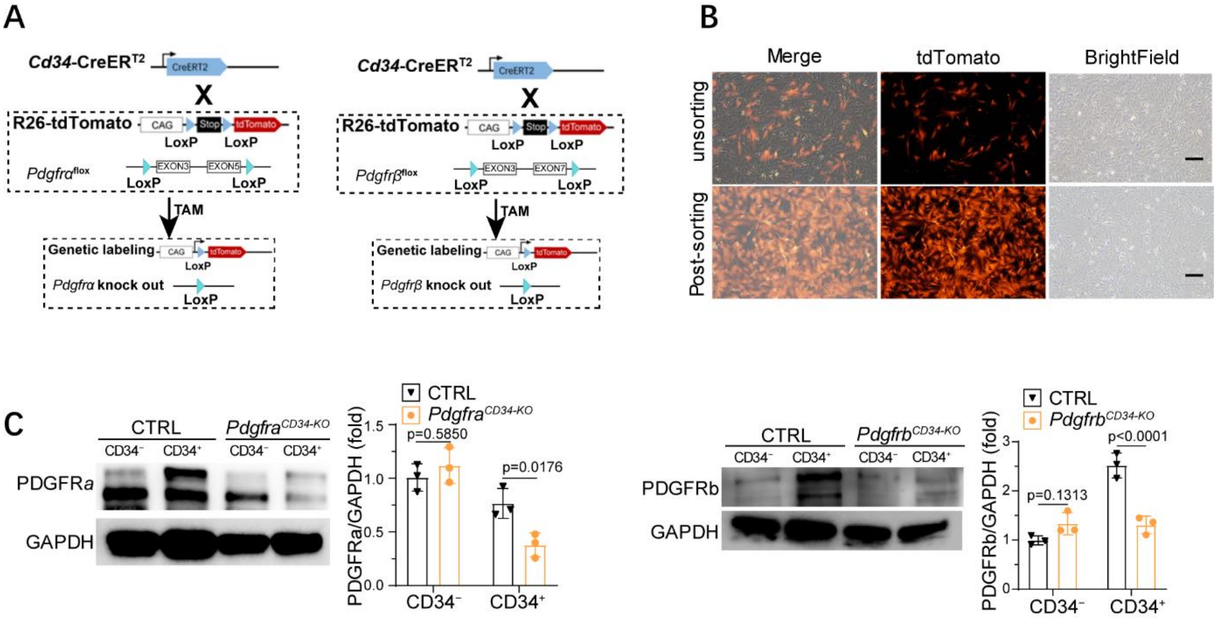

Figure S17 (Related to Figure 6). Establishment of the CD34^{+} cell specific *Pdgfra/Pdgfrb* knockout lineage tracing mice. (A) Schematic image showing the generation of CD34^{+} cell specific *Pdgfra* (*Pdgfra*$^{-/-}$) or *Pdgfrb* ((*Pdgfrb*$^{-/-}$) knockout lineage tracing mouse strain. (B) Representative image showing CD34^{+} aortic adventitia cells from *Pdgfrb*$^{-/-}$ mice before (unsorting) or after (post-sorting) flow cytometric sorting. (C) Representative western blot of PDGFRa and PDGFRb expression in aortic adventitial cells (CD34^{+} or CD34^{-}) isolated from control (CTRL) and *Pdgfra*$^{-/-}$ (left) or *Pdgfrb*$^{-/-}$ (right) mice, respectively, with quantifiaction of the indicated protein abundance normalized to GAPDH in the right. The results present one of three independent experiments.

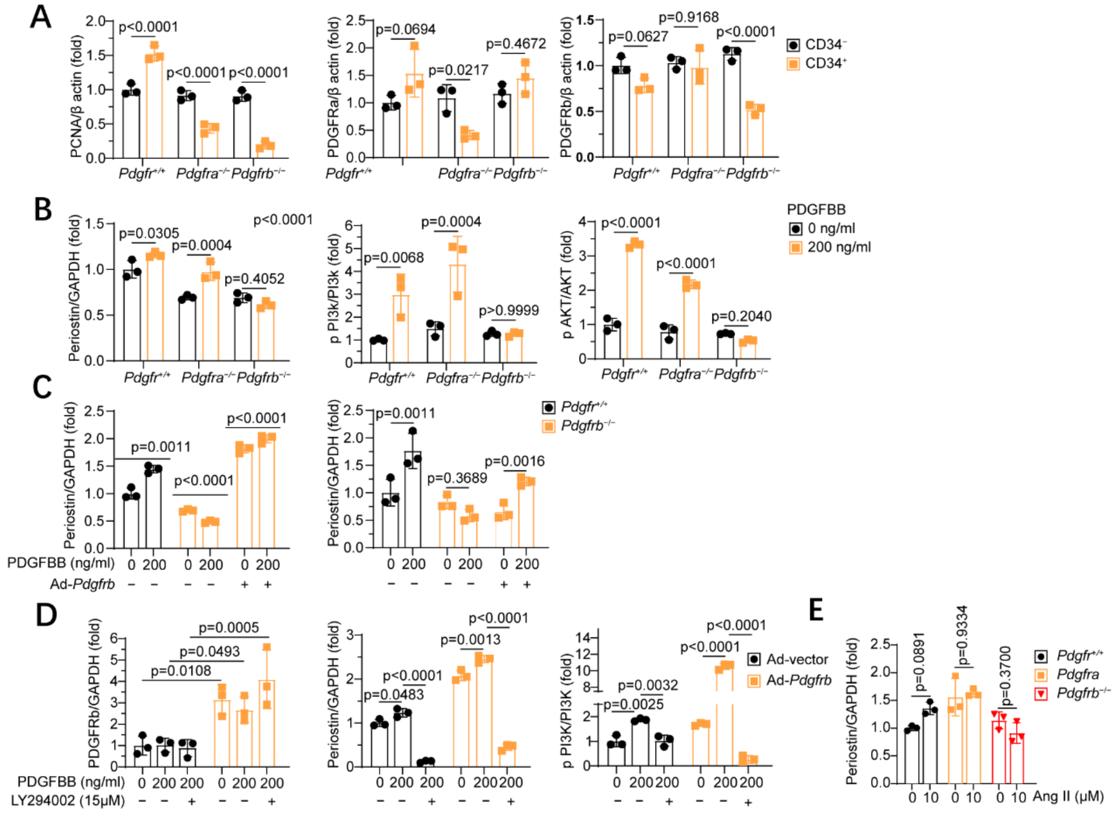

Figure S18 (Related to Figure 7). Quantification of the protein levels in Figure 7. (A) Quantification of the PCNA, PDGFRa and PDGFRb, normalized to β actin in the indicated groups as shown in **Figure** 7B. (B) Quantification for the western blots of protein expressions in indicated cells after PDGFBB stimulation for 12 hours as shown in **Figure** 7H. (C) Quantification for the western blots of Periostin expression in indicated cells with (Ad-*Pdgfrb*) or without PDGFRb overexpression for 48 hours, followed by PDGFBB (200ng/ml) or PBS treatment for 12 hours as shown in **Figure** 7I. (D) Quantification for the western blots of Periostin, total and phosphorylated PI3K protein expressions in *Pdgfrb*$^{-/-}$ cells infected with Ad-vector and Ad-*Pdgfrb* and treated with PDGFBB (200ng/ml) and/or LY294002 (15 μM) as indicated for 12 hours as shown in **Figure** 7L. (E) Quantification for the western blots of Periostin protein expression in indicated cells upon Ang II (10 μM) or PBS stimulation for 12 hours as shown in **Figure** 7M. For A-E, the results present one of three independent experiments, and data are expressed as mean±SD and are tested by 2-way ANOVA test.

.

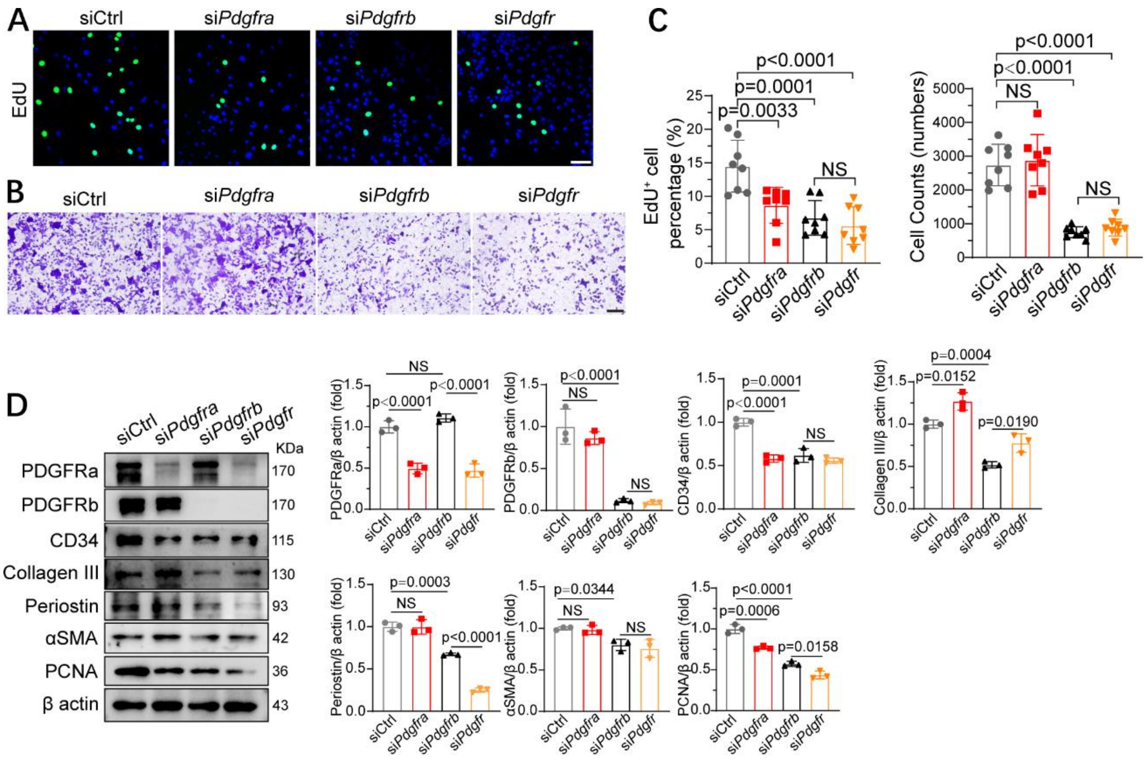

Figure S19 (Related to Figure 7). Knockdown of *Pdgfrb* rather than *Pdgfra* significantly inhibits migration and transdifferentiation of CD34⁺ cells. (A) EdU assay were performed in aortic adventitial CD34⁺ cells transfected with indicated siRNAs, followed by PDGFBB stimulation. scale bar:50 µm. (B) Representative transwell image showing cellular migration capacity of the indicated cells upon PDGFBB treatment. Scale bar: 100 µm. (C) Dotplot (left) displaying the percentages of EdU⁺ cell in primary adventitial cells across different groups (n=8 independent experiments of 5 fields each per group). Quantification (right) of migration cell counts in indicated groups (n=8 independent experiments per group). (D) Western blot showing the expression of cell proliferation marker PCNA and of CD34, cell proliferation marker PCNA, the specific knockdown efficiency of PDGFRa and PDGFRb, and fibrosis-related proteins in in the indicated groups. β actin served as a loading control. Quantification (right) for the western blots of CD34, PCNA and fibrosis-related proteins in response to si*Pdgfra* or/and si*Pdgfrb* treatment in CD34⁺ cells. The results present one of three independent experiments.

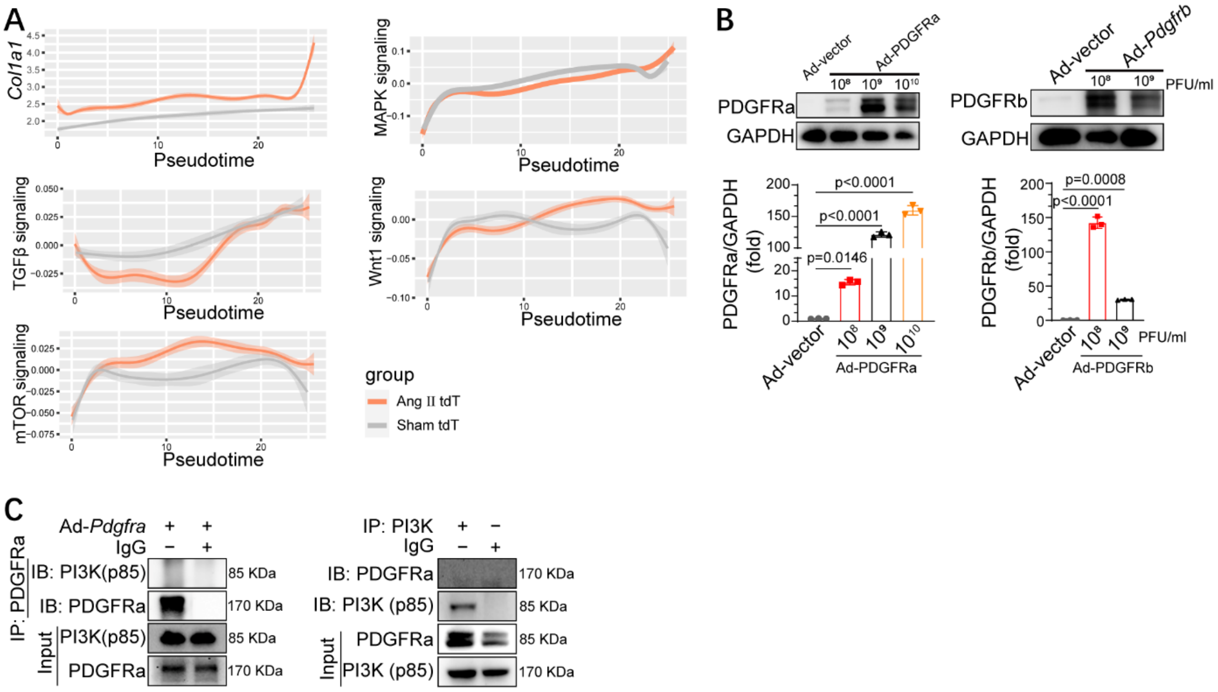

Figure S20 (Related to Figure 7). Pseudotime analysis screening the possible downstream signaling pathways. (A) Curve diagrams displaying the relative expression of indicated proteins or signaling pathways along pseudotime differentiation trajectory. Ang II tdT and Sham tdT indicate tdT^{+}CD45^{-} cells isolated from abdominal aortas of *Cd34*-CreER^{T2};R26-tdT;*Apoe*$^{-/-}$ mice infused with Ang II and saline for 4 weeks, respectively. (B) Representative western blot image showing PDGFRa (Left) and PDGFRb (Right) expression levels in CD34^{+} cells infected with adenovirus expressing *Pdgfra* (Ad-*Pdgfra*) and *Pdgfrb* (Ad-*Pdgfrb*) and Ad-vector control, with quantification of the indicated protein levels normalized to GAPDH. (C) Left, coimmunoprecipitation (Co-IP) analysis of the interaction of PDGFRa and PI3K in CD34^{+} cells infected with adenovirus expressing *Pdgfra* (Ad-*Pdgfra*) and treated with PDGFBB (200ng/ml) for 12 hours. Protein complex was pull-down using anti-IgG or anti-PDGFRa antibody, and detected by antibodies against PI3K (p85) and PDGFRa, respectively. Right, reciprocal co-IP analysis used anti-PI3K antibody to detect endogenous interaction between PI3K and PDGFRa.

Table S1: The clinical information and biochemical characteristics of TAA and AAA patients and corresponding controls. Data are shown as mean±standard deviation.

	Non-dilated AA	AAA	Non-dilated TA	TAA
N(male/female)	6 (6/0)	16 (14/2)	12 (7/5)	21 (14/7)
Age (Years)	42.67±20.72	50.13±13.73	52.92±15.90	53.14±13.89
Body mass index (kg/m^2)	25.10±2.42	24.27±3.86	22.69±1.45	23.26±2.41
Maximal diameters (mm)	/	61.79±13.89	/	52.95±16.62
**Extent of aortic aneurysm**				
Ascending aorta	/	3 (18.75%)	/	14 (66.67%)
Aortic arch	/	0 (0.00)	/	4 (19.05%)
Descending aorta	/	3 (18.75%)	/	4 (19.05%)
abdominal aorta	/	16 (100%)	/	1 (4.76%)
carotid artery	/	0 (0.00)	/	4 (19.05%)
Subclavian artery	/	0 (0.00)	/	4 (19.05%)
Superior mesenteric artery	/	0 (0.00)	/	2 (9.52%)
Iliac arteries	/	2 (12.50%)	/	3 (14.29%)
**Risk factors**				
Smoking	2 (33.33%)	6 (37.50%)	0 (0.00)	3 (14.29%)
Hypertension	2 (33.33%)	4 (25.00%)	1 (8.33%)	3 (14.29%)
Diabetes	0 (0.00)	0 (0.00)	2 (16.67)	0 (0.00)
**Patients history**				
Marfan syndrome	0 (0.00)	2 (12.50%)	0 (0.00)	0 (0.00)
Myocardial ischemia	0 (0.00)	0 (0.00)	1 (8.33%)	1 (4.76%)
Valvulopathy	0 (0.00)	1 (6.25%)	3 (25.00%)	6 (28.57%)
CAD	0 (0.00)	3 (18.75%)	1 (8.33%)	2 (9.52%)
Stroke	3 (50.00%)	1 (6.25%)	0 (0.00)	0 (0.00)

Table S2. Primer Sequences

Gene	Sequence (5'-3')	Comments
Cd34-CreER^{T2}	GTAGGATAGCAGATGGTCTGGACT<br>CTGTTAATACTCACGCAGCAGACT<br>TGTGCCTCAAATCTATTATTTTGC	Genotyping
R26-tdTomato	CTCTGCTGCCTCCTGGCTTCT<br>CGAGGCGGATCACAAGCAATA<br>TCAATGGGCGGGGGTCGTT	Genotyping
R26-DTR	GCGAAGAGTTTGTCCTCAACC<br>AAAGTCGCTCTGAGTTGTTAT<br>GAGCGGGAGAAATGGATATG	Genotyping
Postn-CreER^{T2}	AGGCATTCAGTCTTTCTTACCCT<br>TCAGTTCCTACCCCACAGGA<br>AGGCACACAAGACTACTTCAA<br>TGCGAACCTCATCACTCGTT	Genotyping
Cd34-Dre	CAACAGGGAGGGGATAAGCC<br>AGCCTCAACTTTGAGCTTTGC<br>AGAGGCAGCCAAGATGACAC<br>AGAGATAGGGAACCAGGCCA	Genotyping
Dou-tdT-DTR	TCAGATTCTTTTATAGGGGACACA<br>TAAAGGCCACTCAATGCTCACTAA<br>ATGAAGCTGCTGCCGTCGG<br>TCAGTGGGAATTAGTCATGCCCAA	Genotyping
Pdgfra-Flox	CAGACATTACTCCAAAGTTAGGCACC<br>TCTTTCCTCACTGCCACCCTCT	Genotyping
Pdgfrb-Flox	GTGATGGACACATGCCAGGTACTA<br>GAGGGGCACATTCTTGCCTGTCTG	Genotyping
Apoe$^{-/-}$	GCCTAGCCGAGGGAGAGCCG<br>TGTGACTTGGGAGCTCTGCAGC<br>GCCGCCCCGACTGCATCT	Genotyping
18s	CGGAAAATAGCCTTCGCCATCAC<br>ATCACTCGCTCCACCTCATCCT	qPCR
Cd34	AGGACAGCAGTAAGACCACACC<br>GTGTGGAGTTCCAGAGCCTGAA	qPCR
Postn	CAGCAAACCACTTTCACCGACC<br>AGAAGGCGTTGGTCCATGCTCA	qPCR
Fbn1	GCTGTGAATGCGACATGGGCTT<br>TCTCACACTCGCAACGGAAGAG	qPCR
Tcf21	CGTCCAGCTACATCGCTCACTT<br>CAGGTCATTCTCTGGTTTGCCG	qPCR
Pdgfra	GCAGTTGCCTTACGACTCCAGA<br>GGTTTGAGCATCTTCACAGCCAC	qPCR
Pdgfrb	GTGGTCCTTACCGTCATCTCTC<br>GTGGAGTCGTAAGGCAACTGCA	qPCR
si*Pdgfra*-sense	CAGCGAGUUUAAUGUUUAUTT	siRNA
si*Pdgfra*-anti-sense	AUAAACAUUAAACUCGCUGTT	

**si*Pdgfrb*-sense**	GAGCAAGGAUGAAUCUAUATT	siRNA
**si*Pdgfrb*-anti-sense**	UAUAGAUUCAUCCUUGCUCTT	
**rAAV8-D377Y-mPCSK9**	TTACATGAAGGGTCTGGCAGC<br>CATAGCGTAAAAGGAGCAACA	Plasmids construction
**Ad-*Pdgfra***	CGCAAATGGGCGGTAGGCGTG<br>CTGCATTCTAGTTGTGGTTTGTC	Plasmids construction
**Ad-*Pdgfrb***	CGCAAATGGGCGGTAGGCGTG<br>CTGCATTCTAGTTGTGGTTTGTC	Plasmids construction
